# Supplementary figures and images for: Evolution of host-microbe cell adherence by receptor domain shuffling
Source: eLife. 2022 Jan 25;11:e73330. doi: 10.7554/eLife.73330 (PMC8860441; doi:10.7554/eLife.73330)

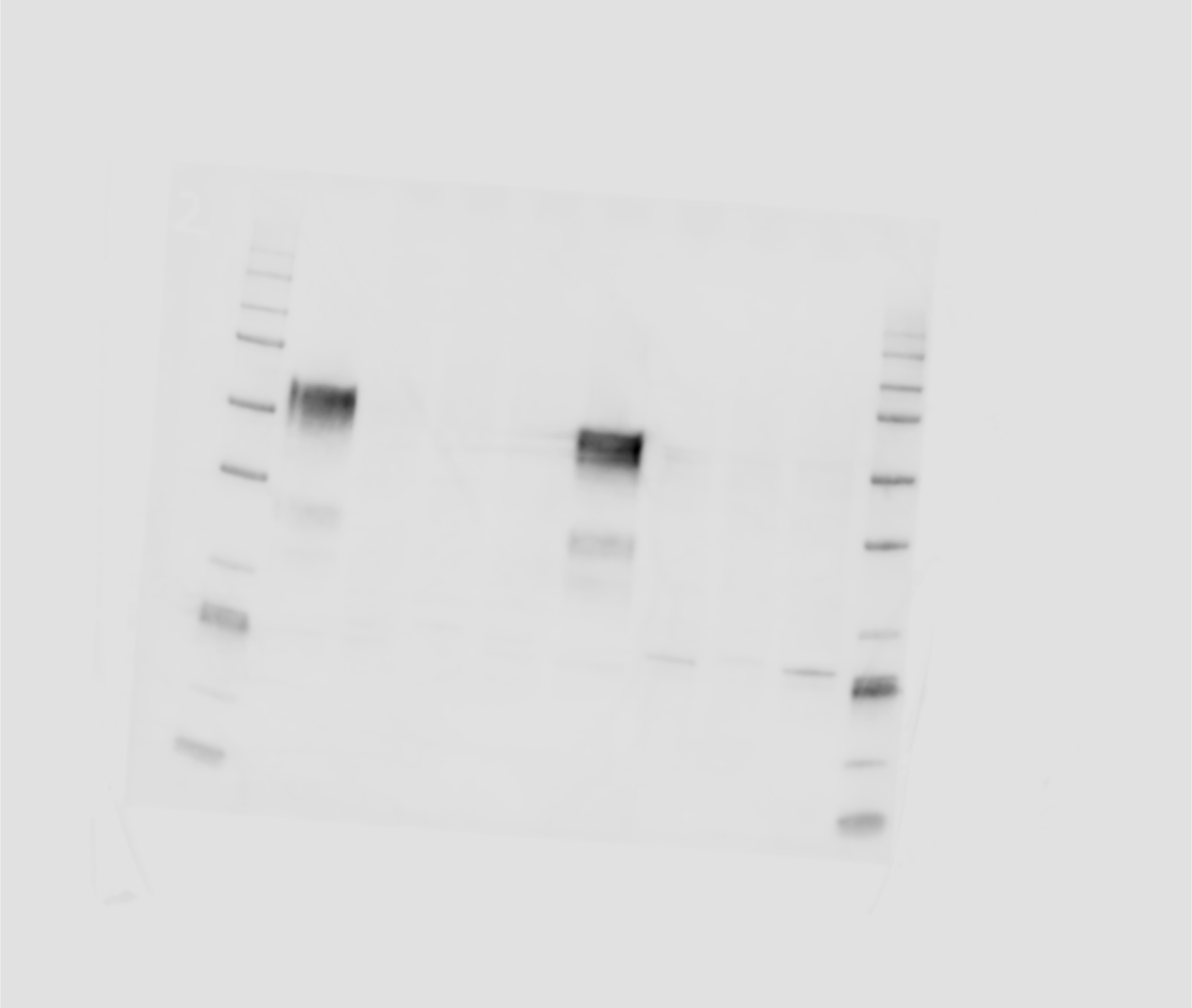

Supplement: Figure 3—source data 1. [file elife-73330-fig3-data1.zip › Figure 3 - Source data 1/western_images/Hpylori/Baboon_Orangutan.png]

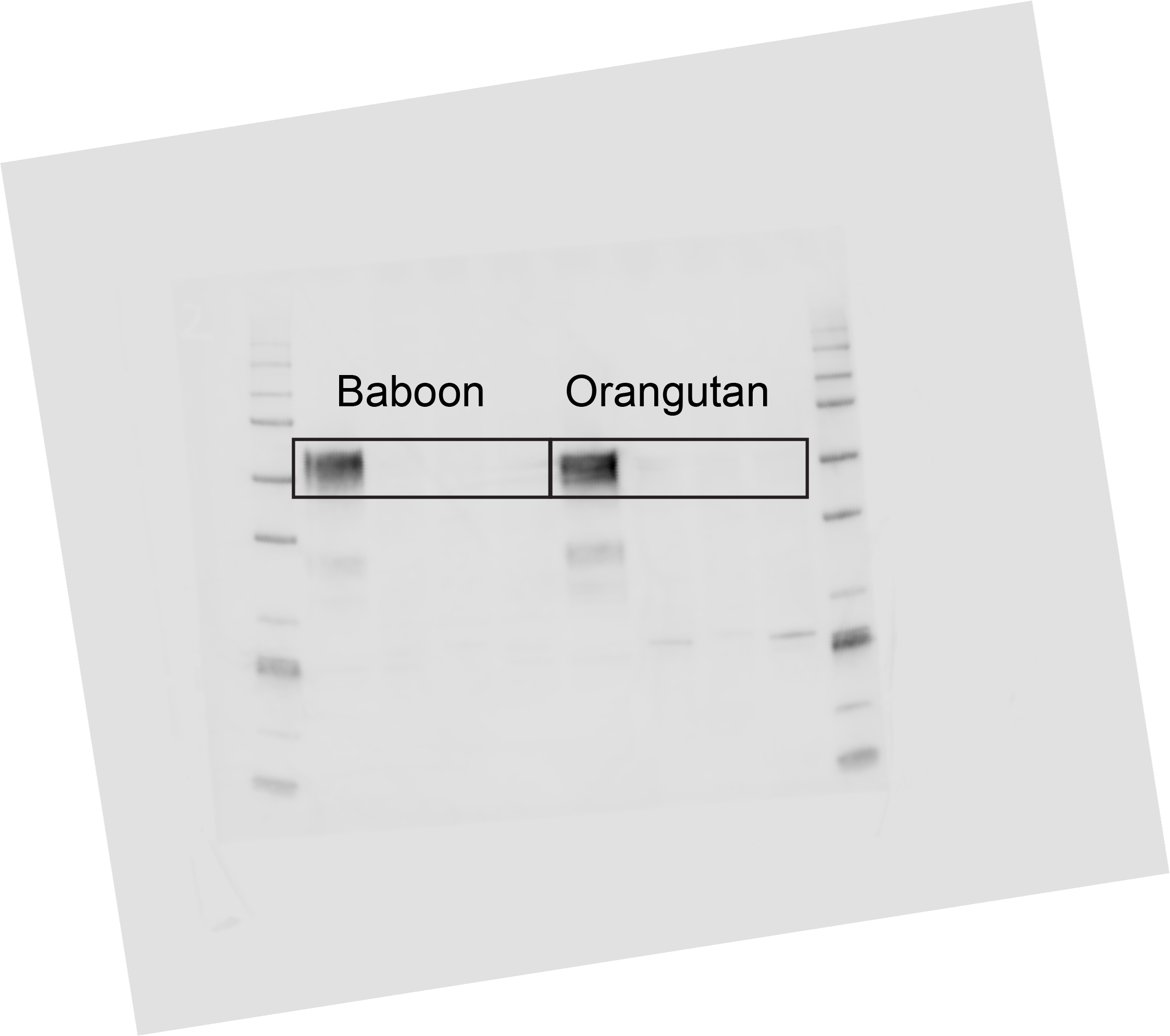

Supplement: Figure 3—source data 1. [file elife-73330-fig3-data1.zip › Figure 3 - Source data 1/western_images/Hpylori/Baboon_Orangutan_label.png]

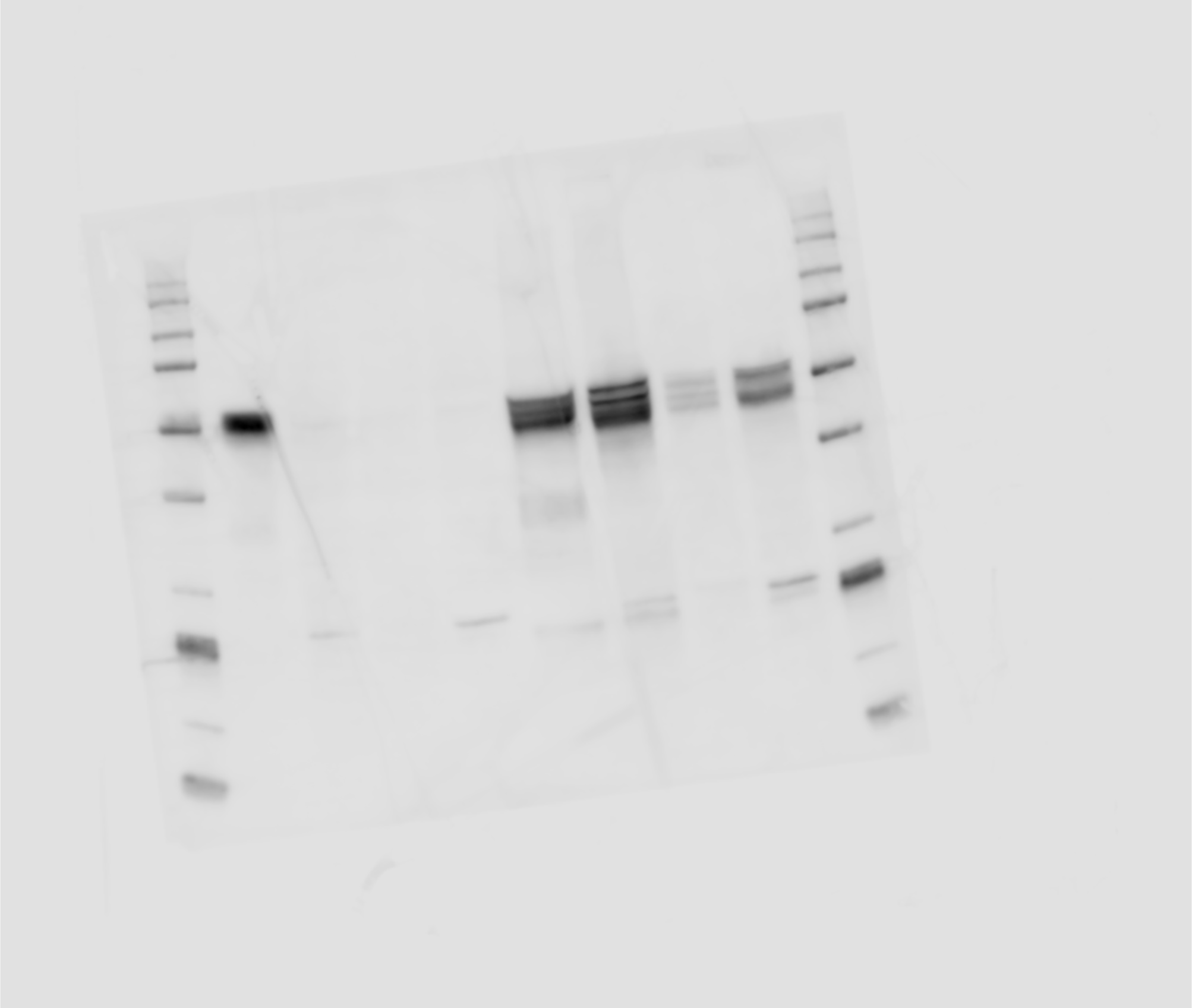

Supplement: Figure 3—source data 1. [file elife-73330-fig3-data1.zip › Figure 3 - Source data 1/western_images/Hpylori/Bonobo_Gorilla_Hp.png]

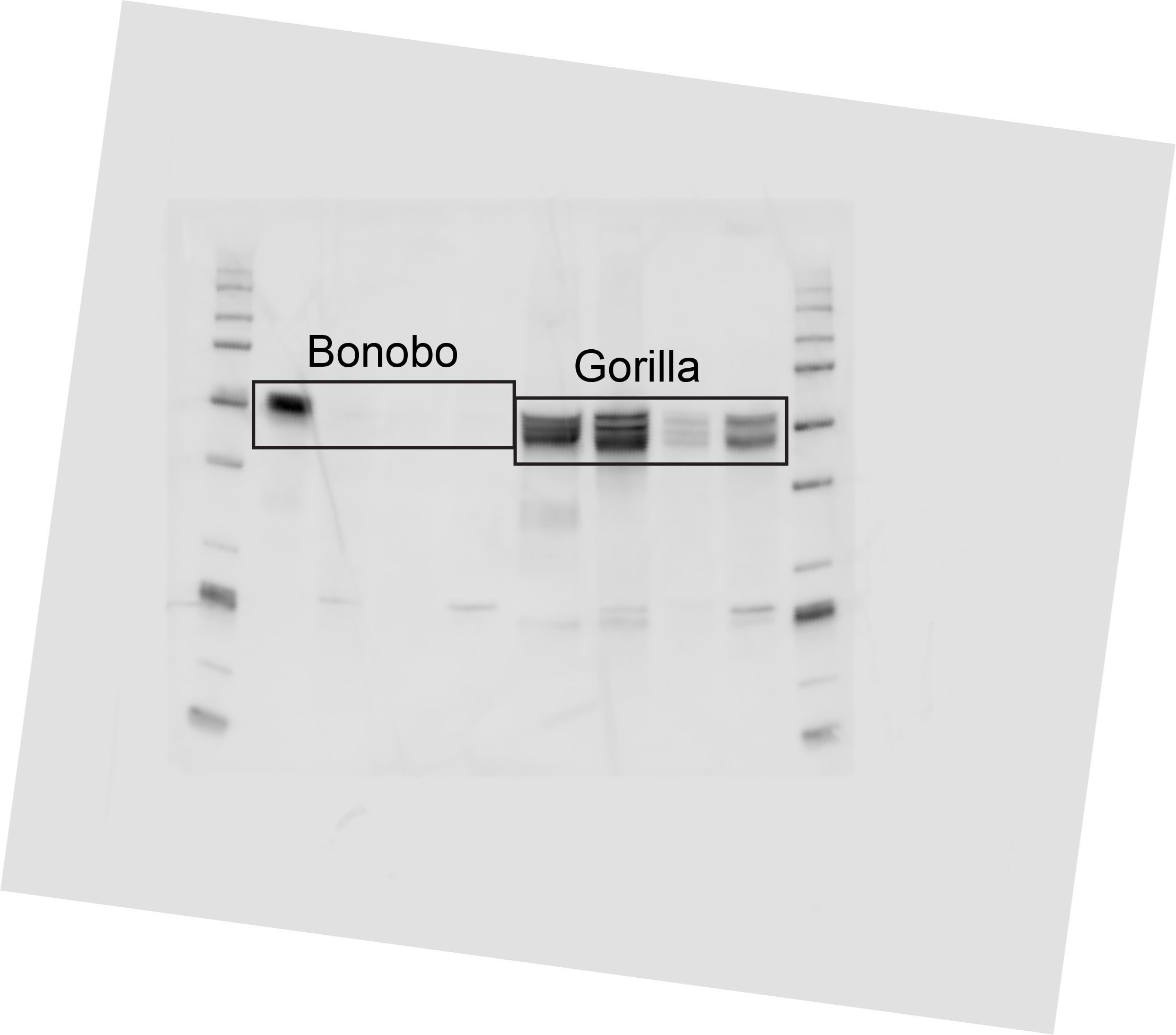

Supplement: Figure 3—source data 1. [file elife-73330-fig3-data1.zip › Figure 3 - Source data 1/western_images/Hpylori/Bonobo_Gorilla_Hp_label.png]

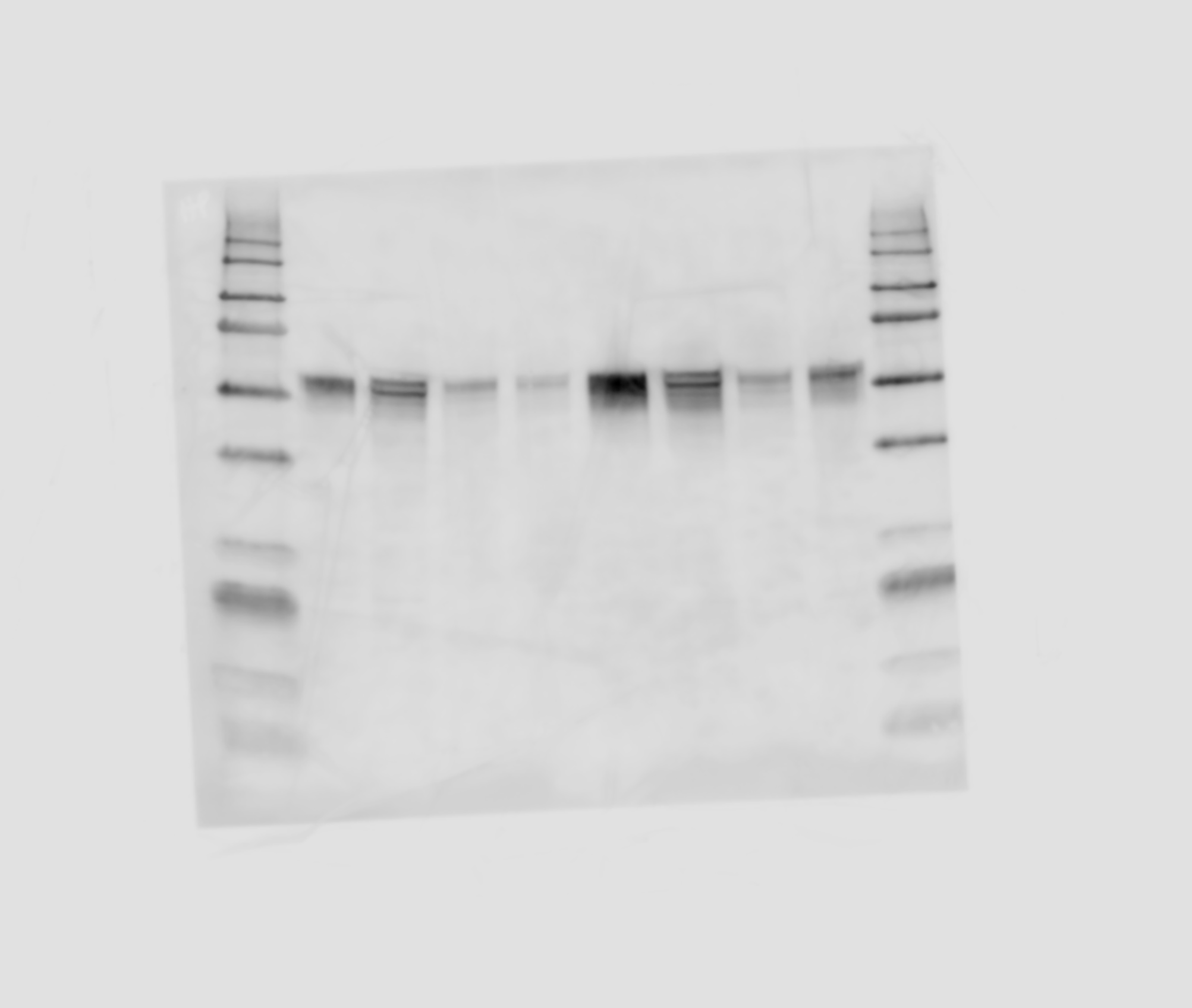

Supplement: Figure 3—source data 1. [file elife-73330-fig3-data1.zip › Figure 3 - Source data 1/western_images/Hpylori/Human_Chimpanzee.png]

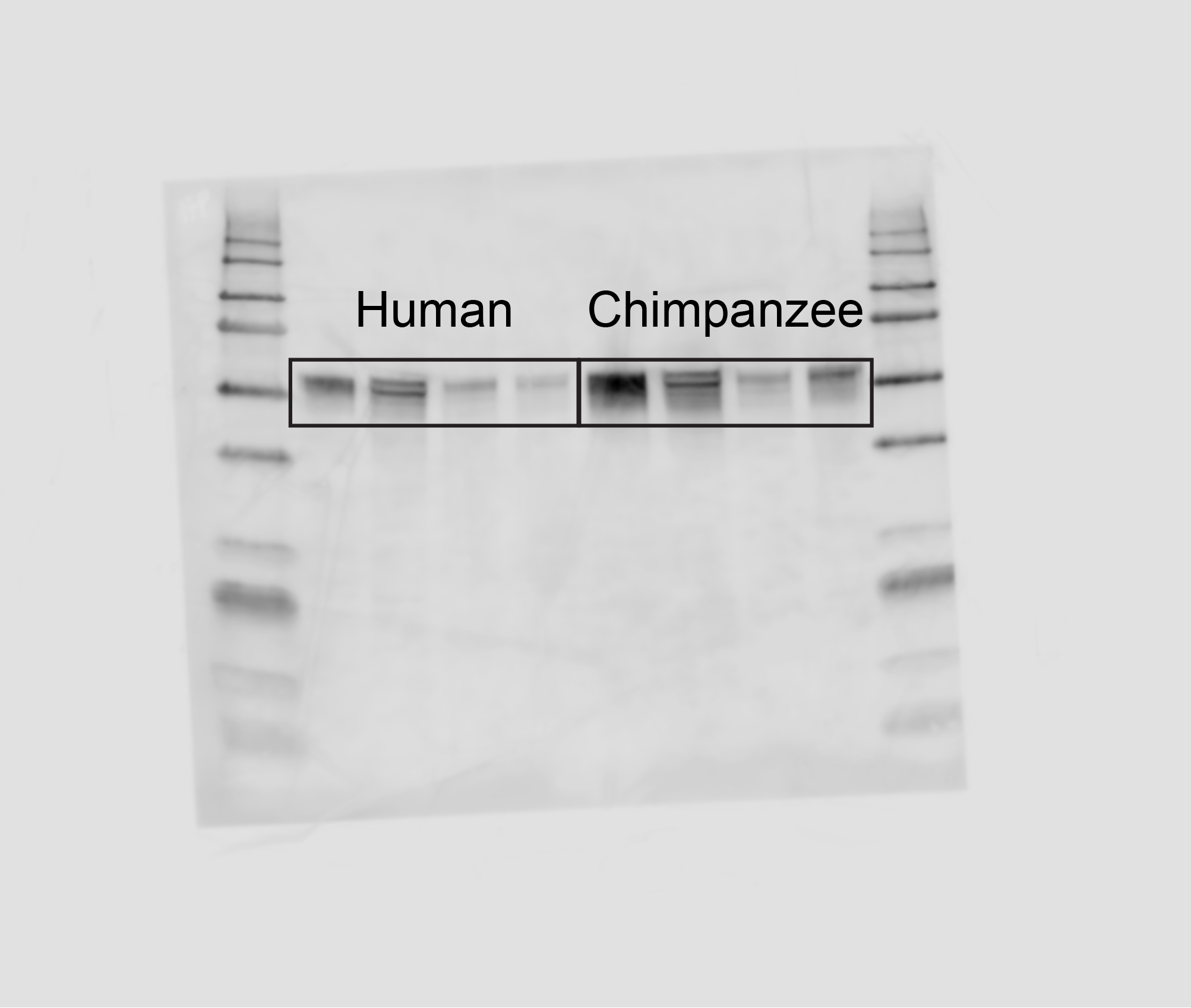

Supplement: Figure 3—source data 1. [file elife-73330-fig3-data1.zip › Figure 3 - Source data 1/western_images/Hpylori/Human_Chimpanzee_label.png]

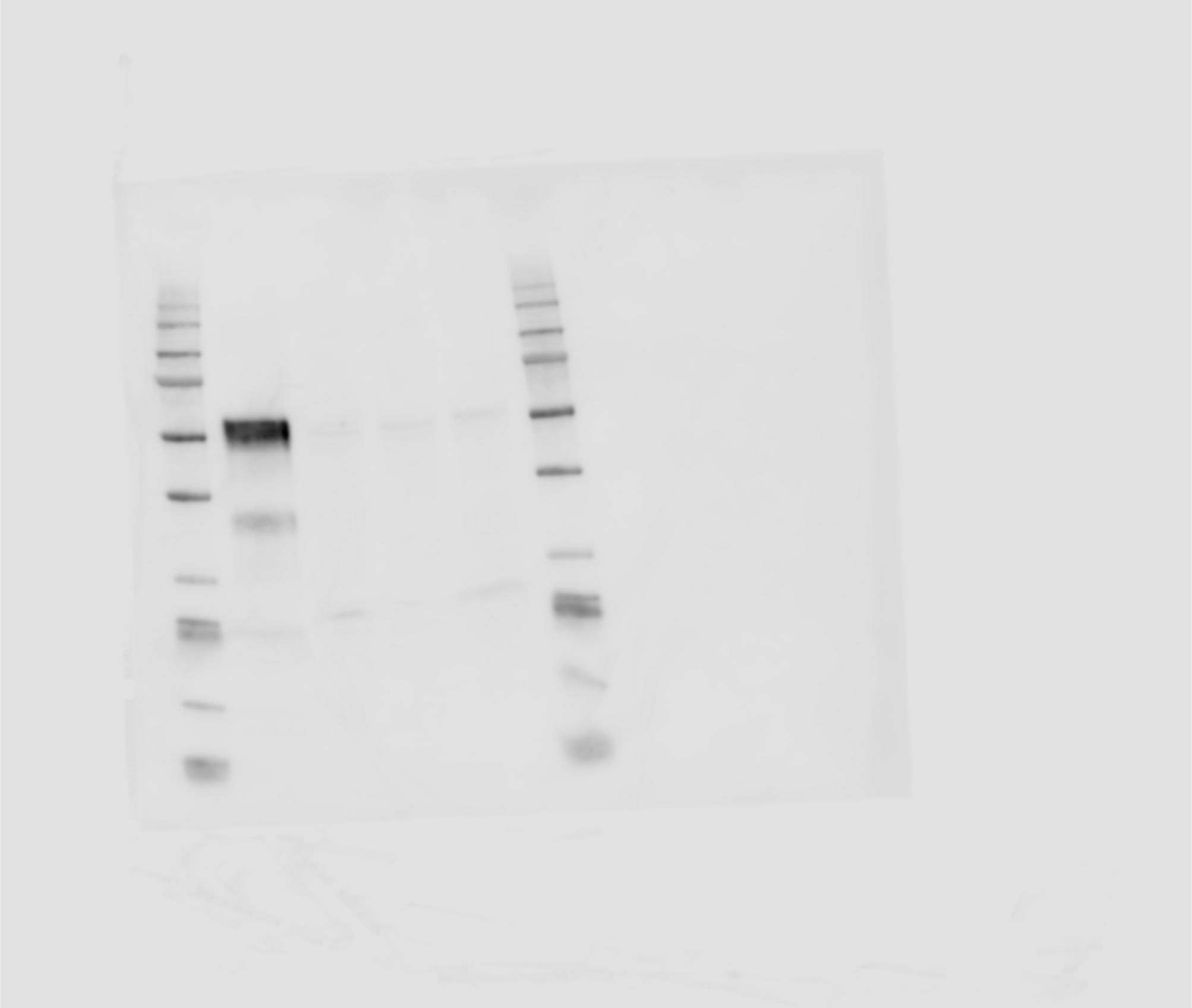

Supplement: Figure 3—source data 1. [file elife-73330-fig3-data1.zip › Figure 3 - Source data 1/western_images/Hpylori/SquirrelMonkey.png]

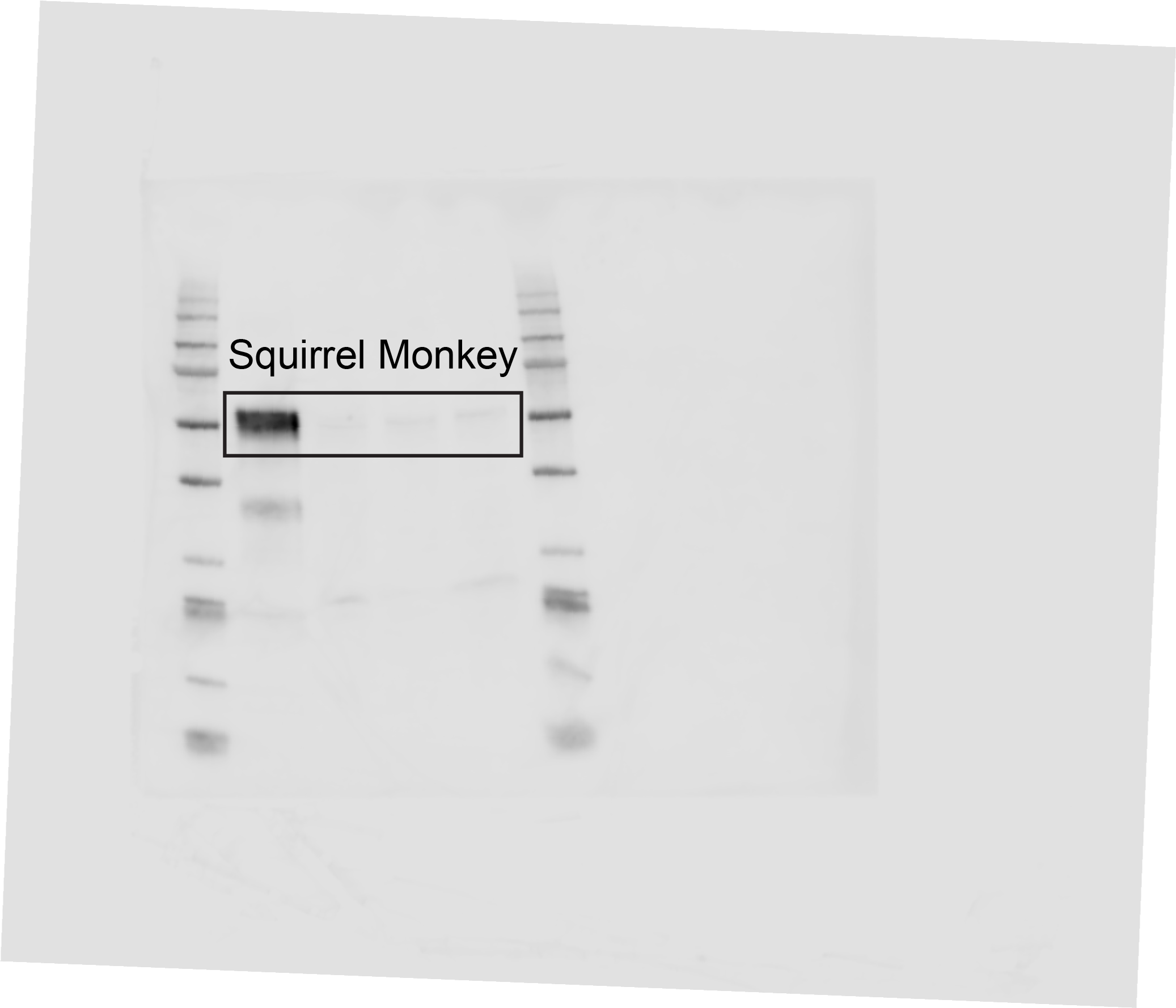

Supplement: Figure 3—source data 1. [file elife-73330-fig3-data1.zip › Figure 3 - Source data 1/western_images/Hpylori/SquirrelMonkey_label.png]

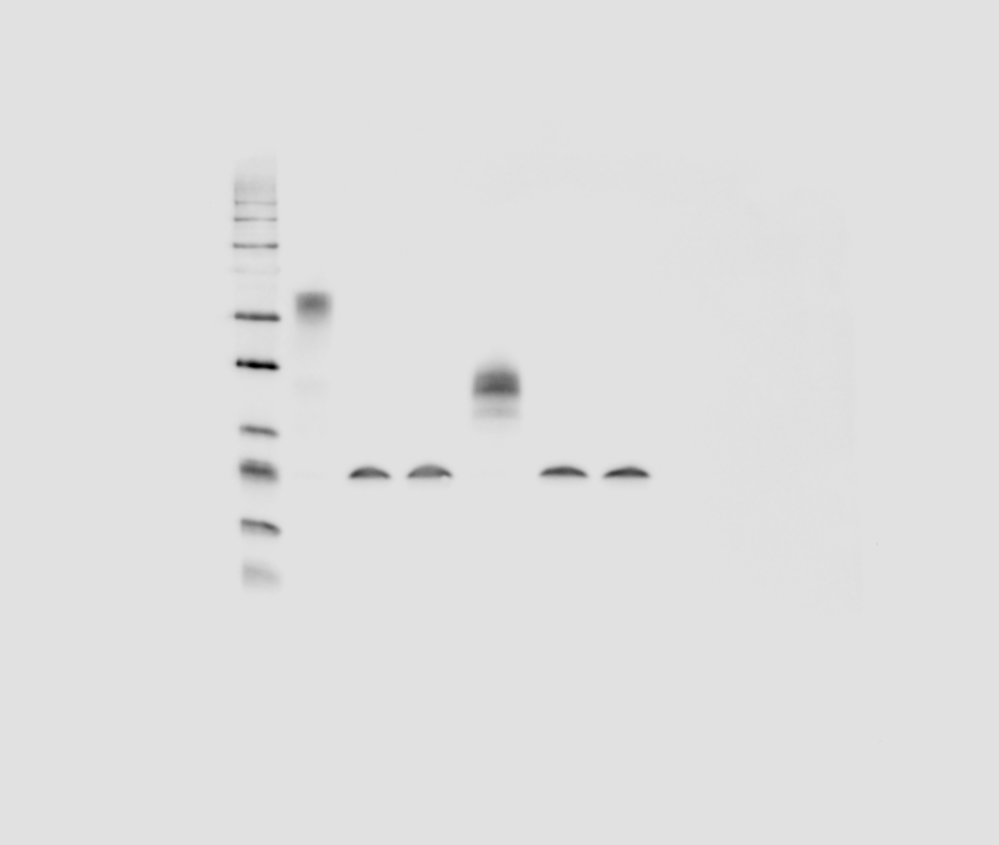

Supplement: Figure 3—source data 1. [file elife-73330-fig3-data1.zip › Figure 3 - Source data 1/western_images/Opa/Baboon.png]

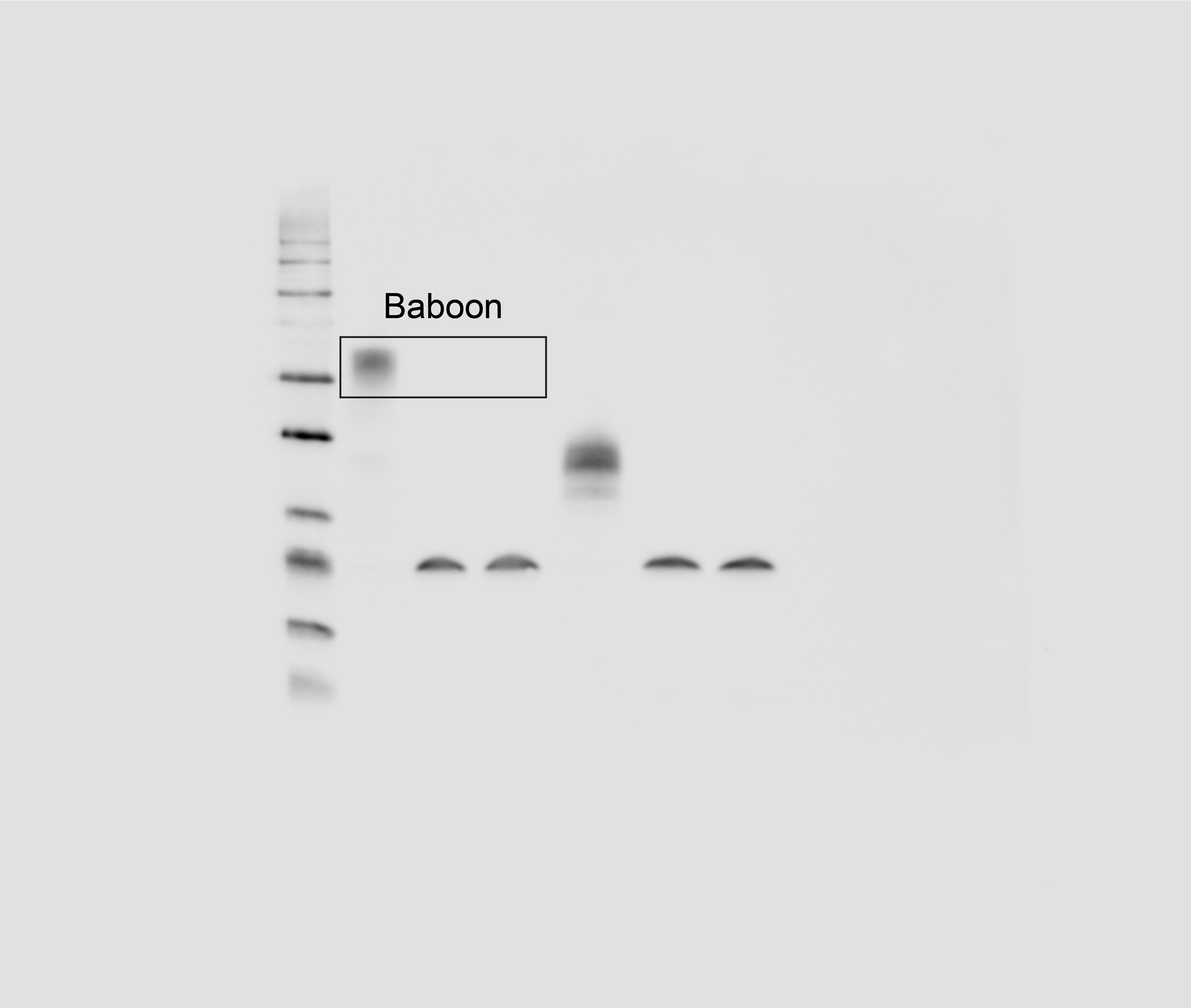

Supplement: Figure 3—source data 1. [file elife-73330-fig3-data1.zip › Figure 3 - Source data 1/western_images/Opa/Baboon_label.png]

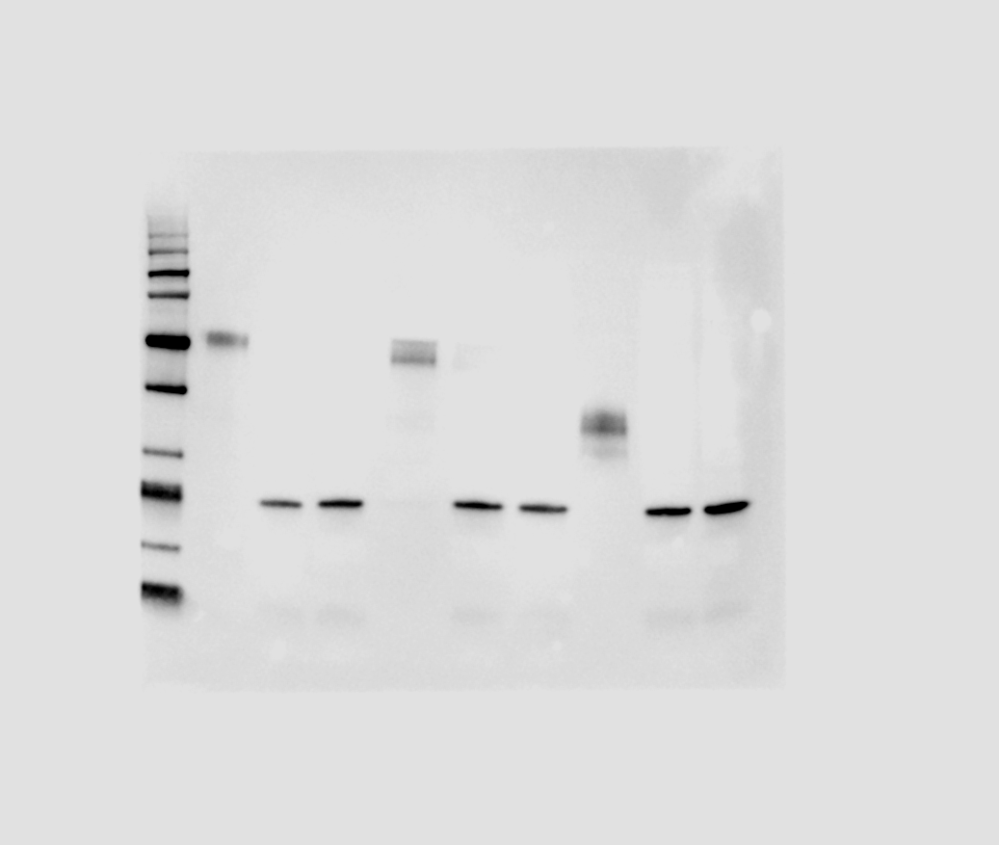

Supplement: Figure 3—source data 1. [file elife-73330-fig3-data1.zip › Figure 3 - Source data 1/western_images/Opa/Bonobo.png]

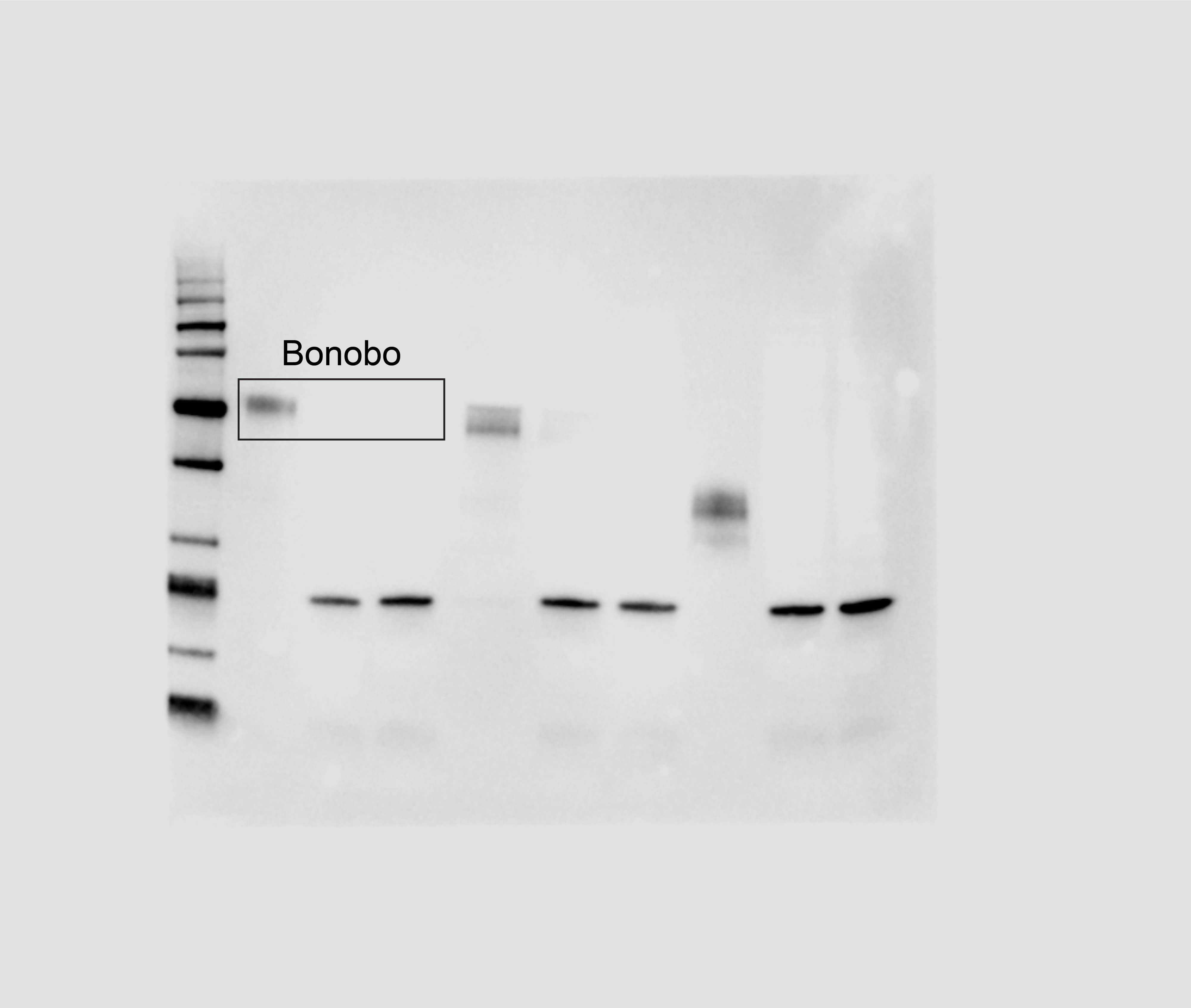

Supplement: Figure 3—source data 1. [file elife-73330-fig3-data1.zip › Figure 3 - Source data 1/western_images/Opa/Bonobo_label.png]

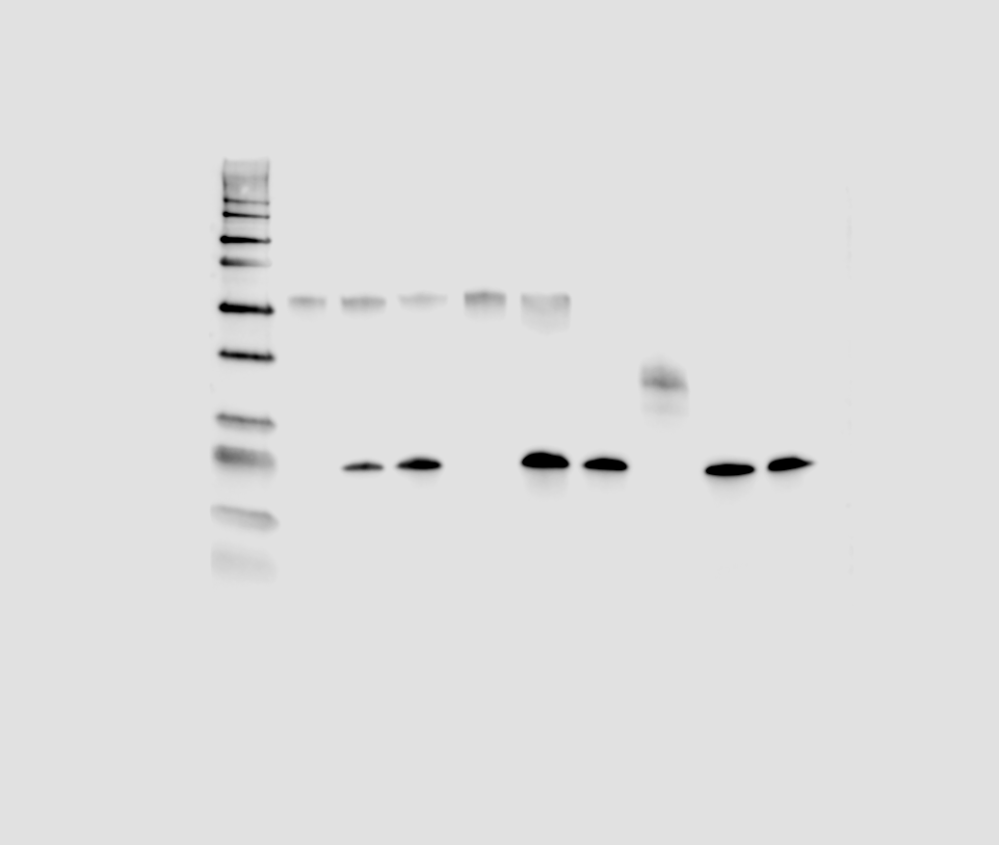

Supplement: Figure 3—source data 1. [file elife-73330-fig3-data1.zip › Figure 3 - Source data 1/western_images/Opa/Chimpanzee.png]

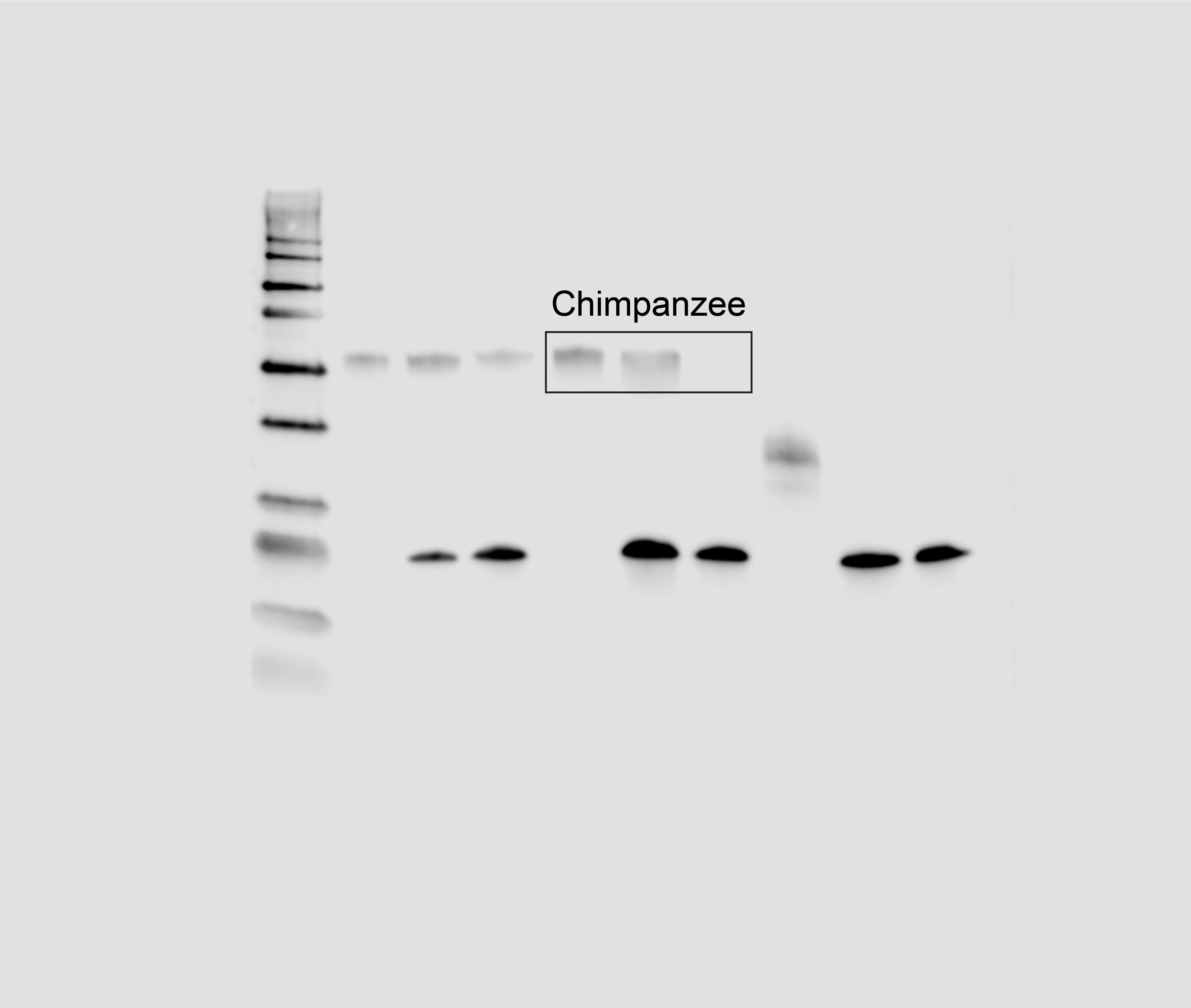

Supplement: Figure 3—source data 1. [file elife-73330-fig3-data1.zip › Figure 3 - Source data 1/western_images/Opa/Chimpanzee_label.png]

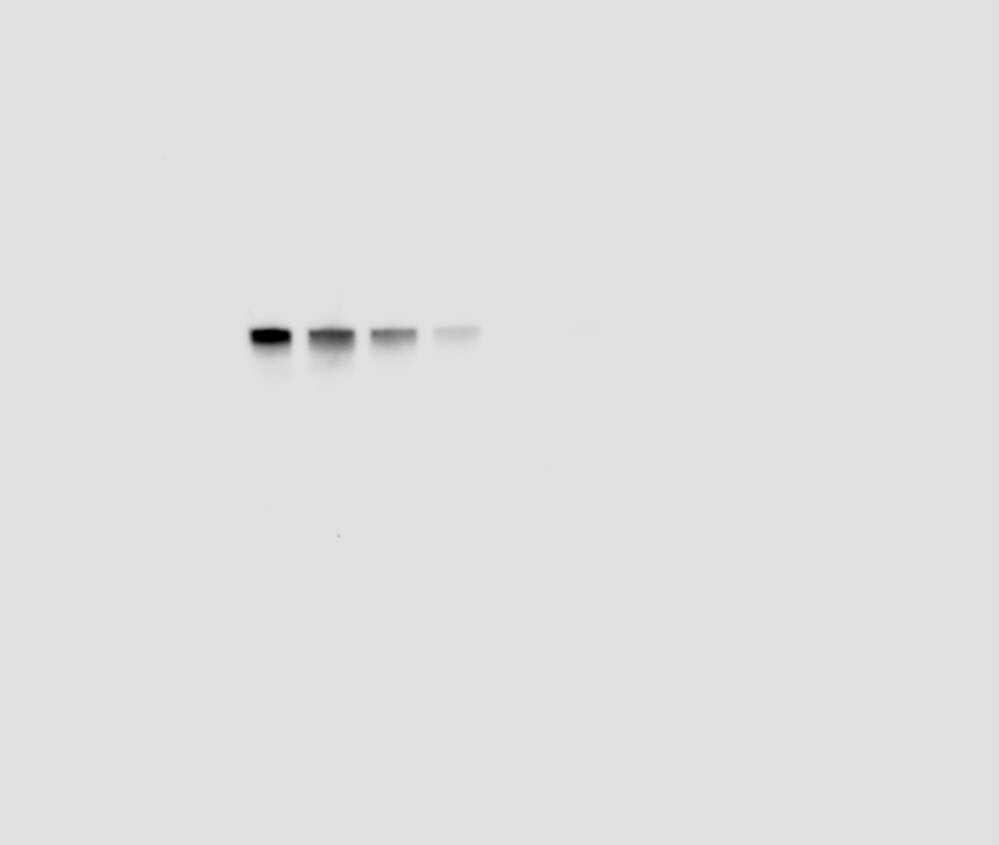

Supplement: Figure 3—source data 1. [file elife-73330-fig3-data1.zip › Figure 3 - Source data 1/western_images/Opa/Human.jpg]

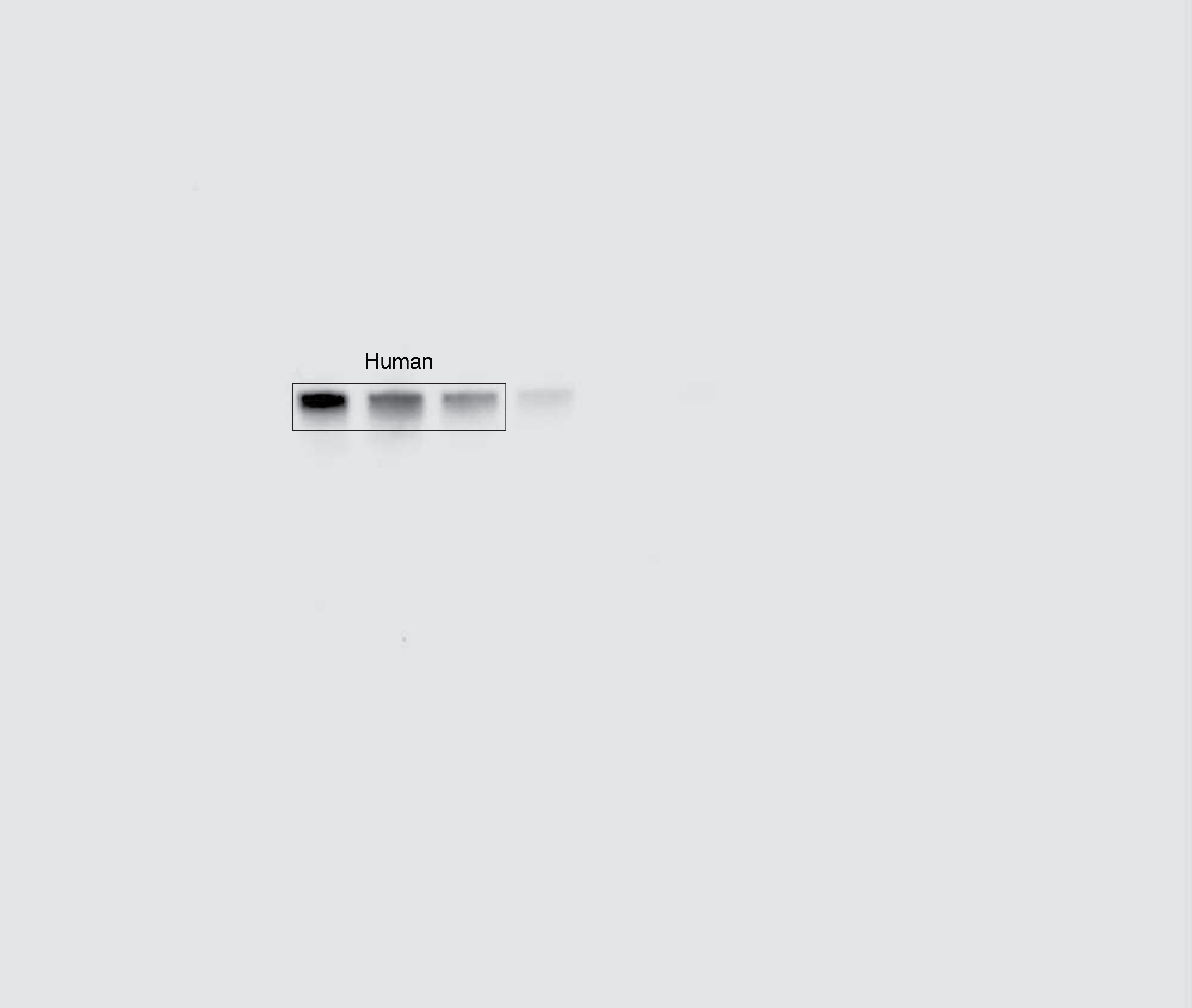

Supplement: Figure 3—source data 1. [file elife-73330-fig3-data1.zip › Figure 3 - Source data 1/western_images/Opa/Human_label.png]

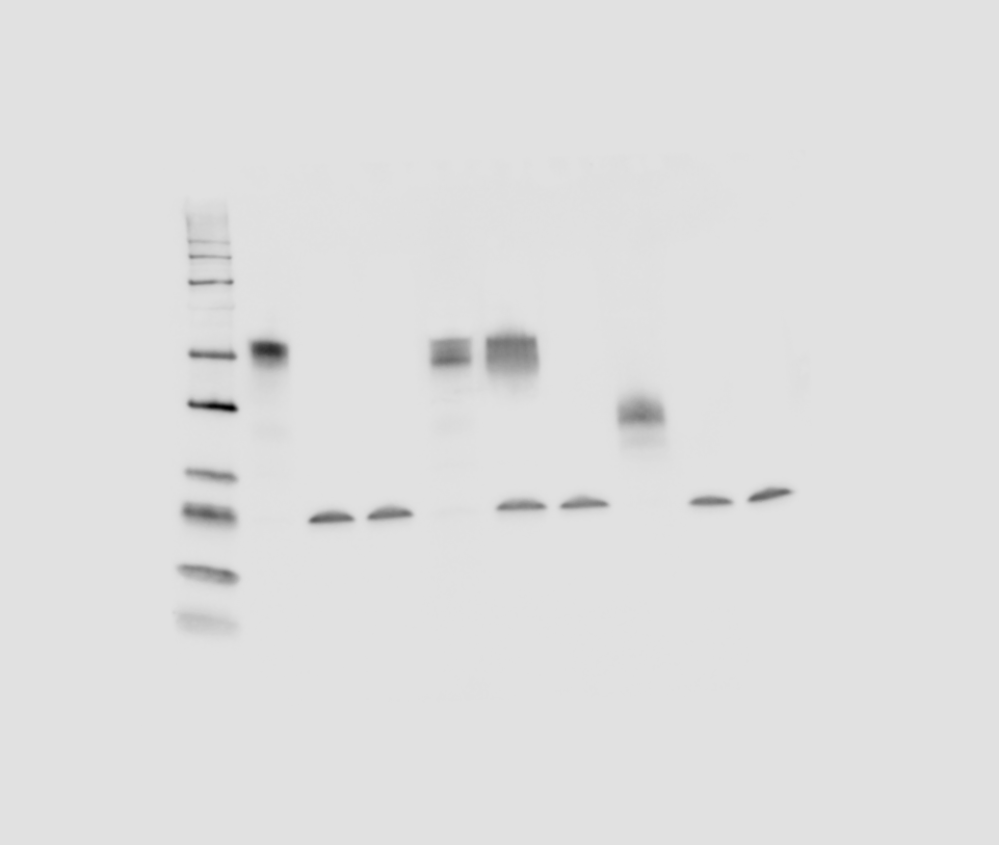

Supplement: Figure 3—source data 1. [file elife-73330-fig3-data1.zip › Figure 3 - Source data 1/western_images/Opa/Orangutan_Gorilla.png]

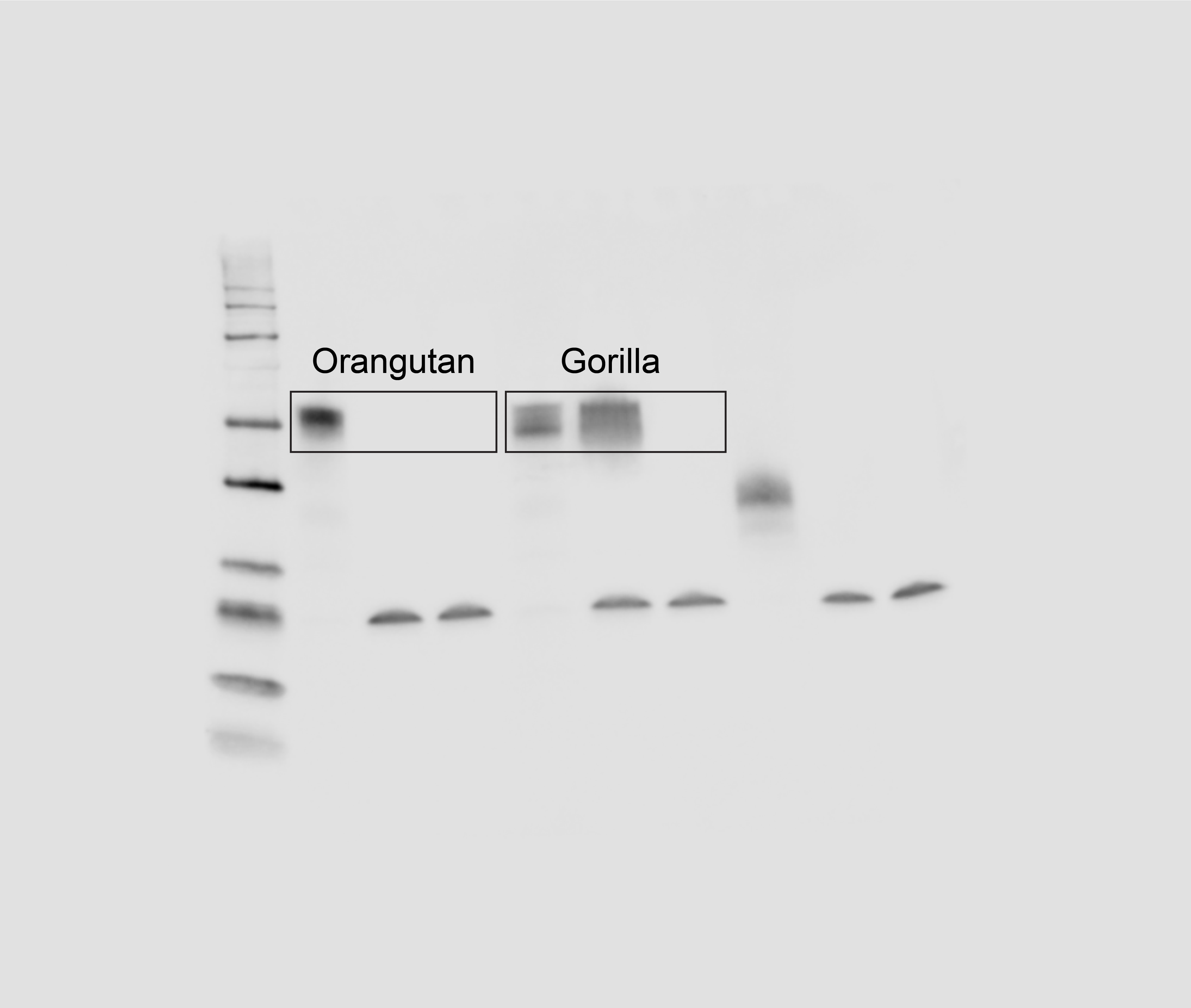

Supplement: Figure 3—source data 1. [file elife-73330-fig3-data1.zip › Figure 3 - Source data 1/western_images/Opa/Orangutan_Gorilla_label.png]

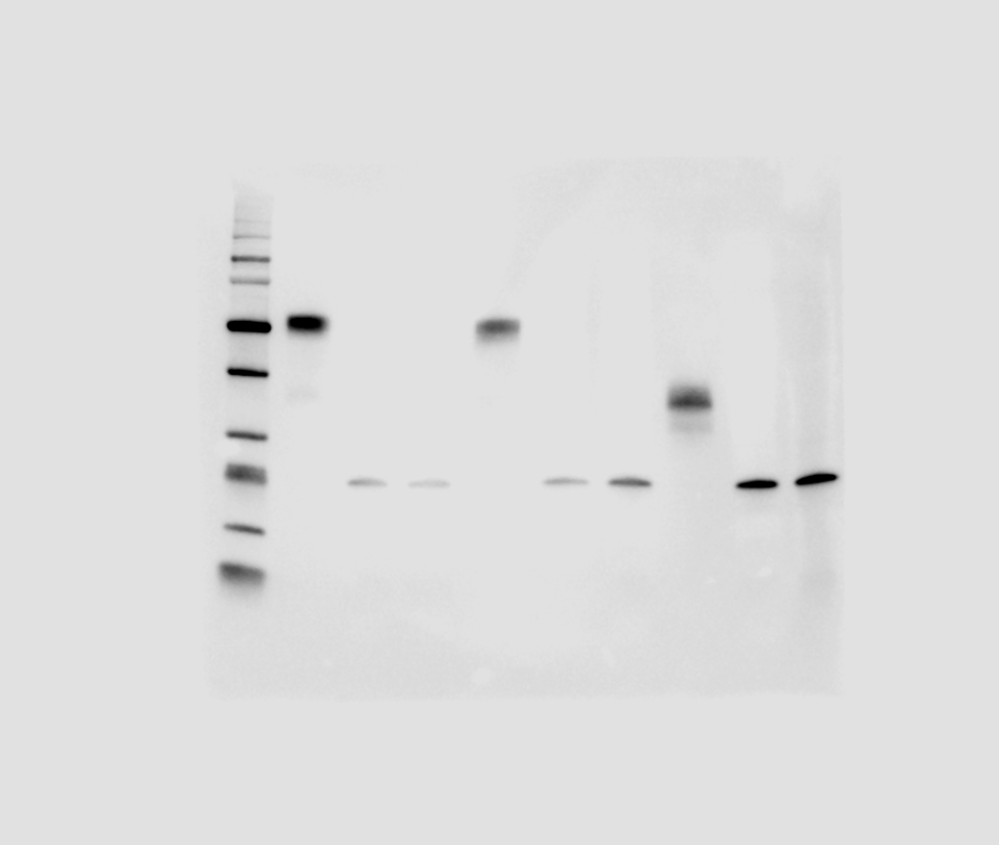

Supplement: Figure 3—source data 1. [file elife-73330-fig3-data1.zip › Figure 3 - Source data 1/western_images/Opa/SquirrelMonkey.png]

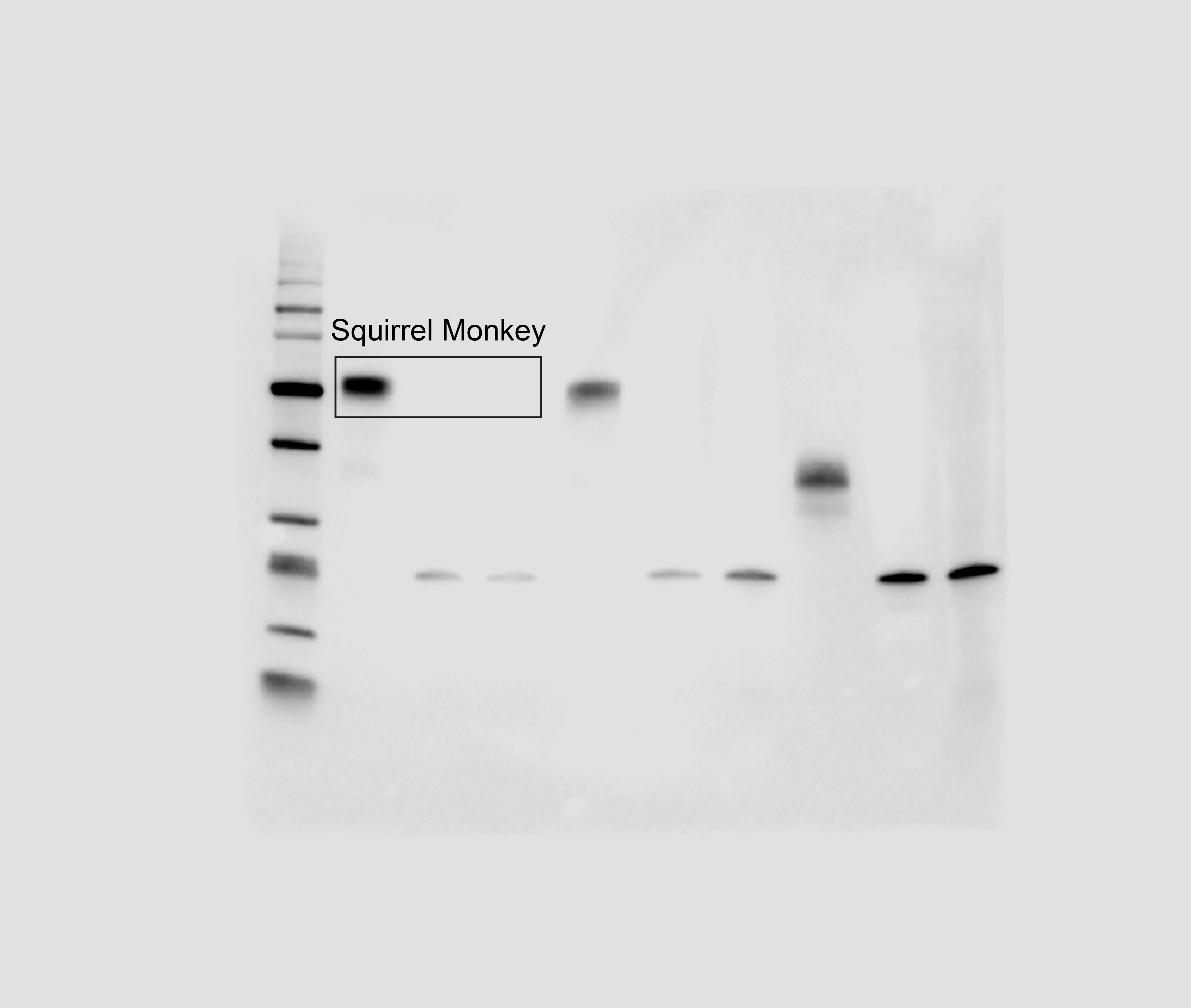

Supplement: Figure 3—source data 1. [file elife-73330-fig3-data1.zip › Figure 3 - Source data 1/western_images/Opa/SquirrelMonkey_label.png]

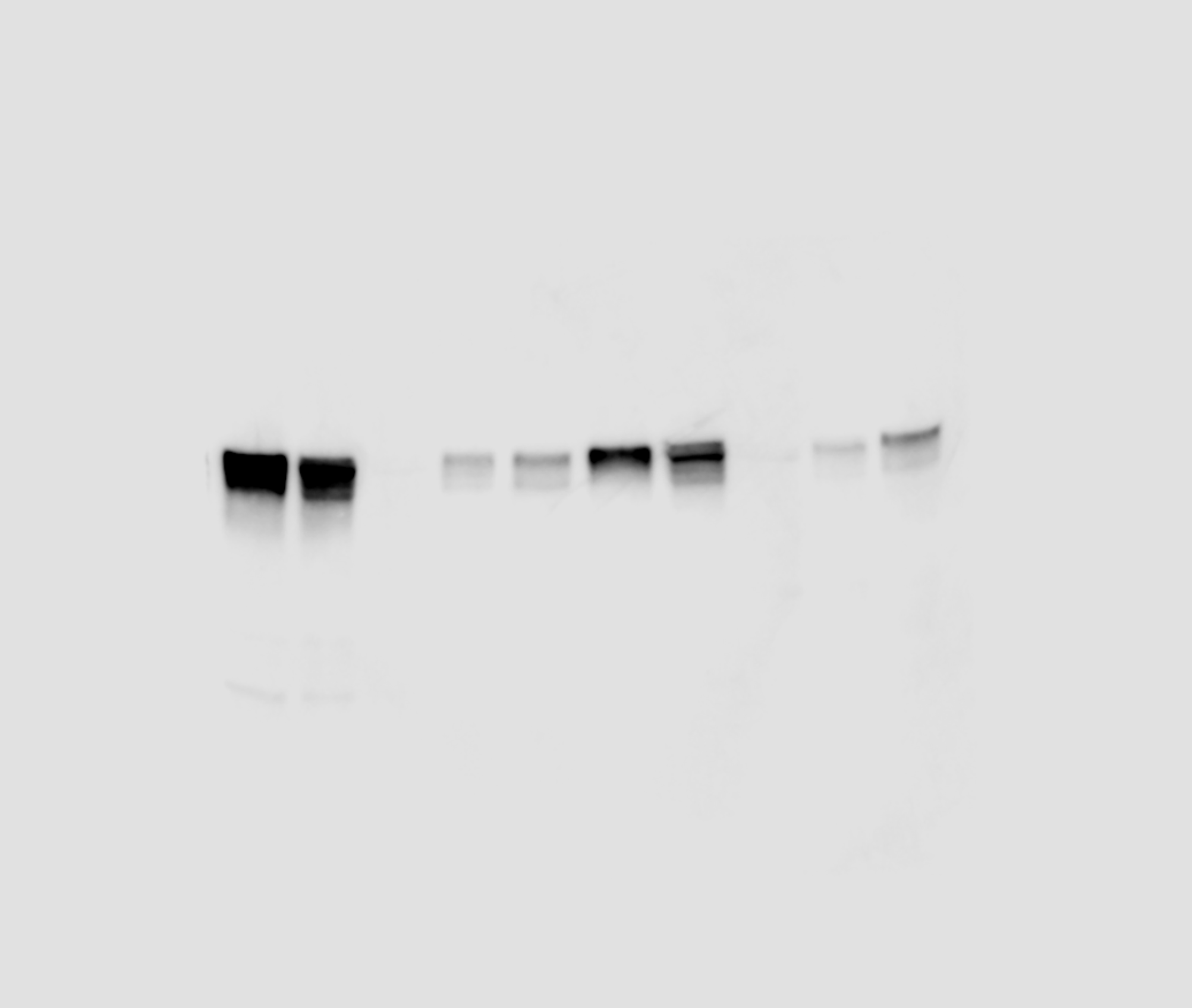

Supplement: Figure 3—figure supplement 1—source data 1. [file elife-73330-fig3-figsupp1-data1.zip › Figure 3 -figure supplement 1 - source data 1/Chimp_Gorilla.png]

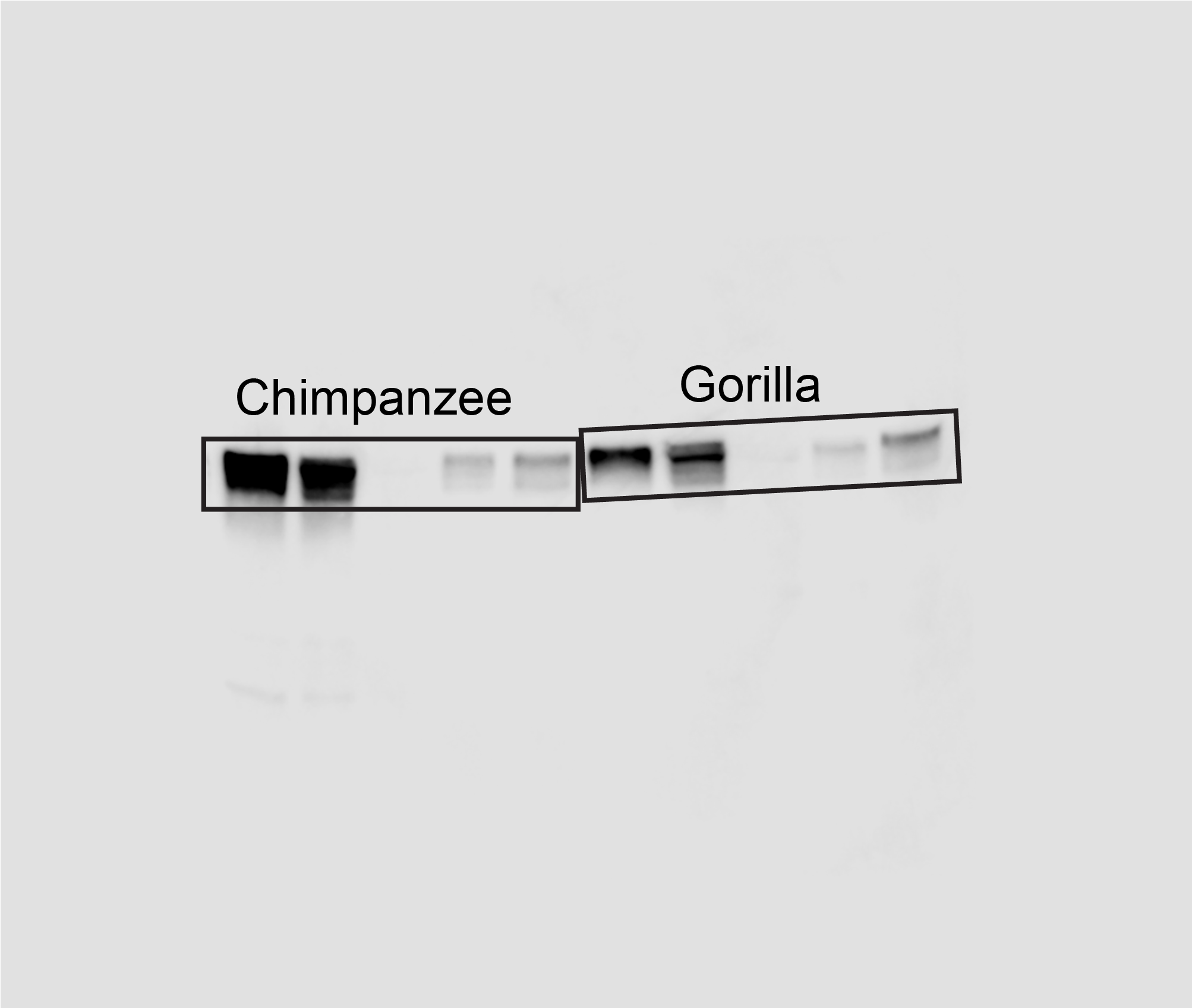

Supplement: Figure 3—figure supplement 1—source data 1. [file elife-73330-fig3-figsupp1-data1.zip › Figure 3 -figure supplement 1 - source data 1/Chimp_Gorilla_label.png]

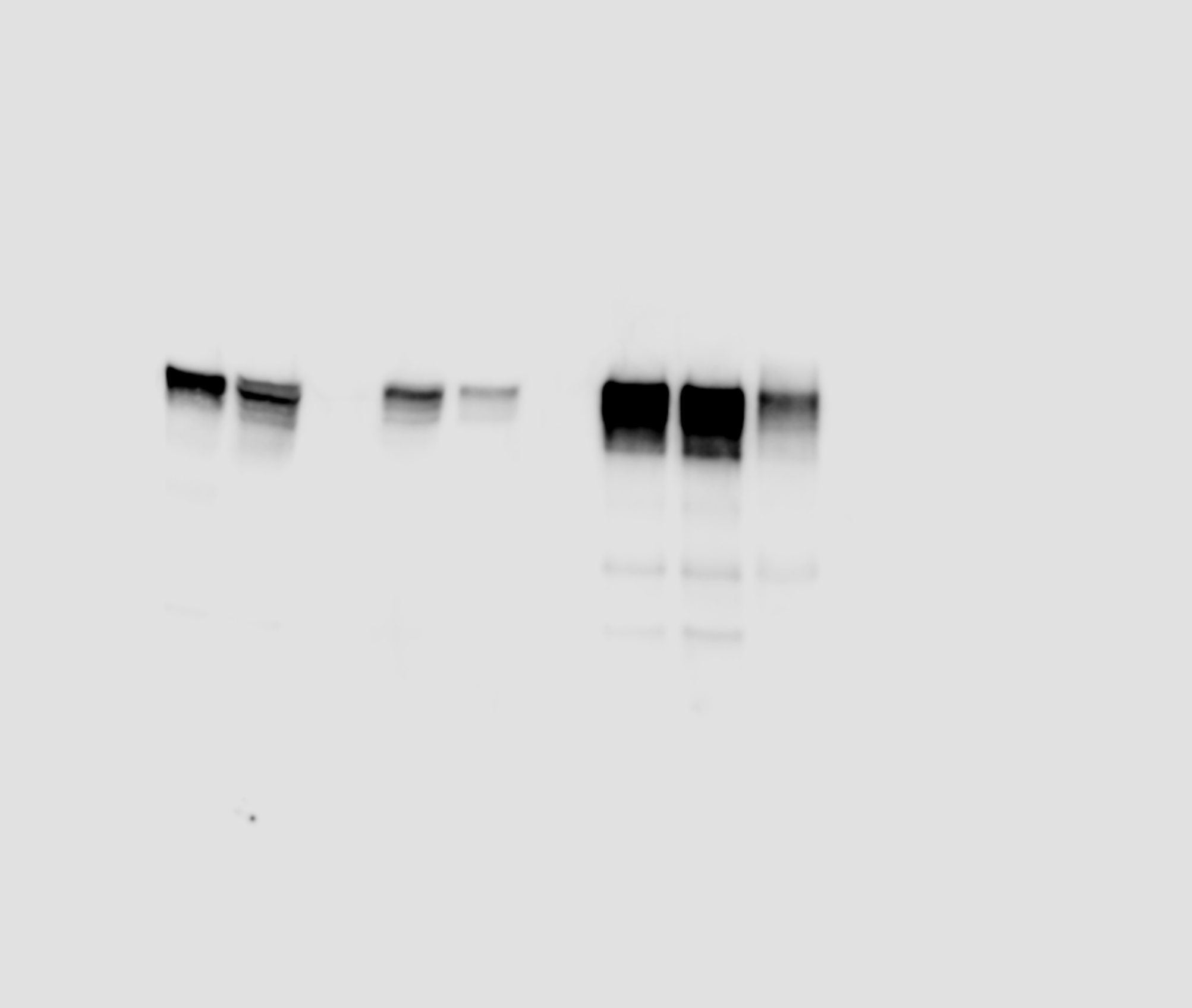

Supplement: Figure 3—figure supplement 1—source data 1. [file elife-73330-fig3-figsupp1-data1.zip › Figure 3 -figure supplement 1 - source data 1/Human.png]

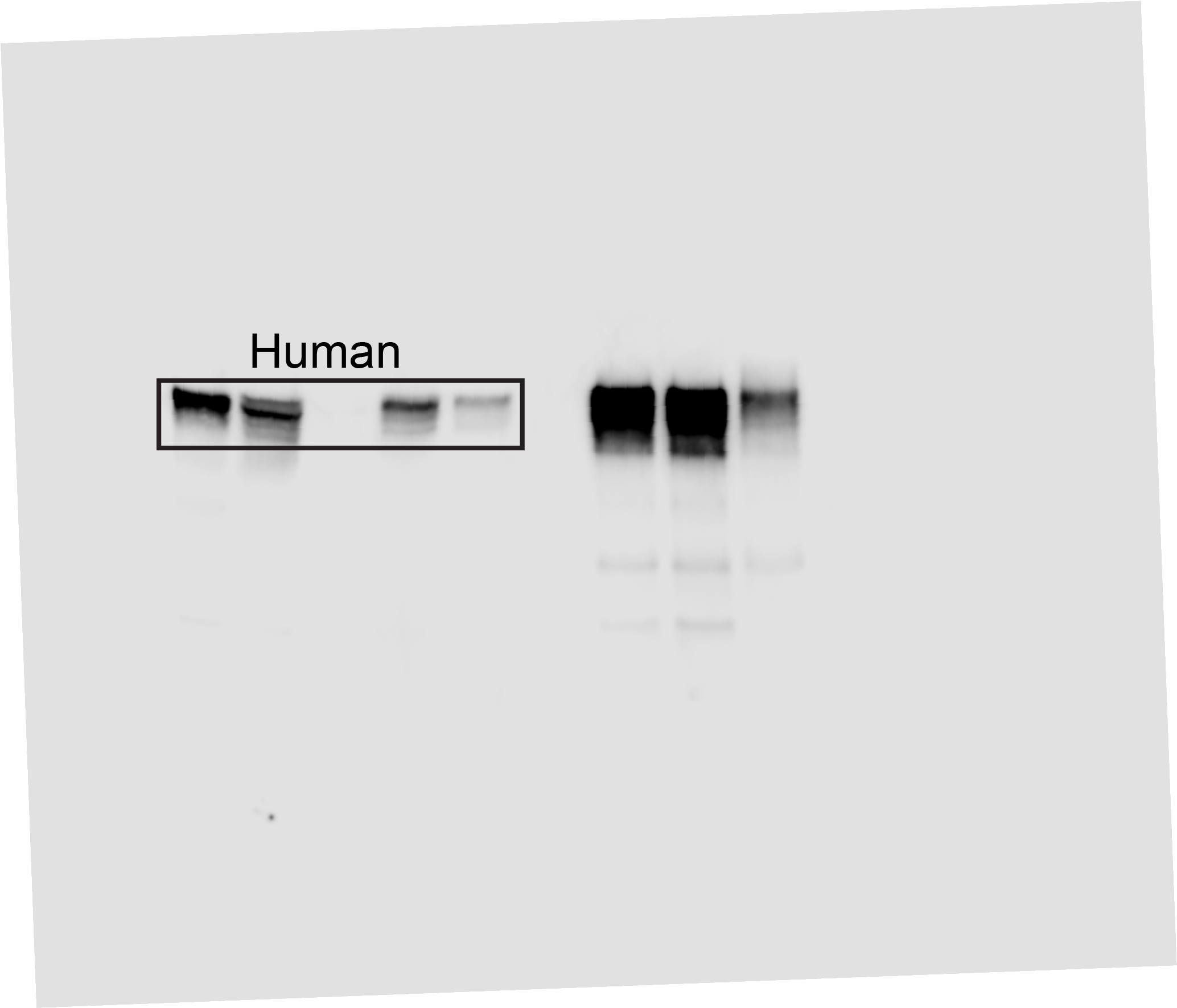

Supplement: Figure 3—figure supplement 1—source data 1. [file elife-73330-fig3-figsupp1-data1.zip › Figure 3 -figure supplement 1 - source data 1/Human_label.png]

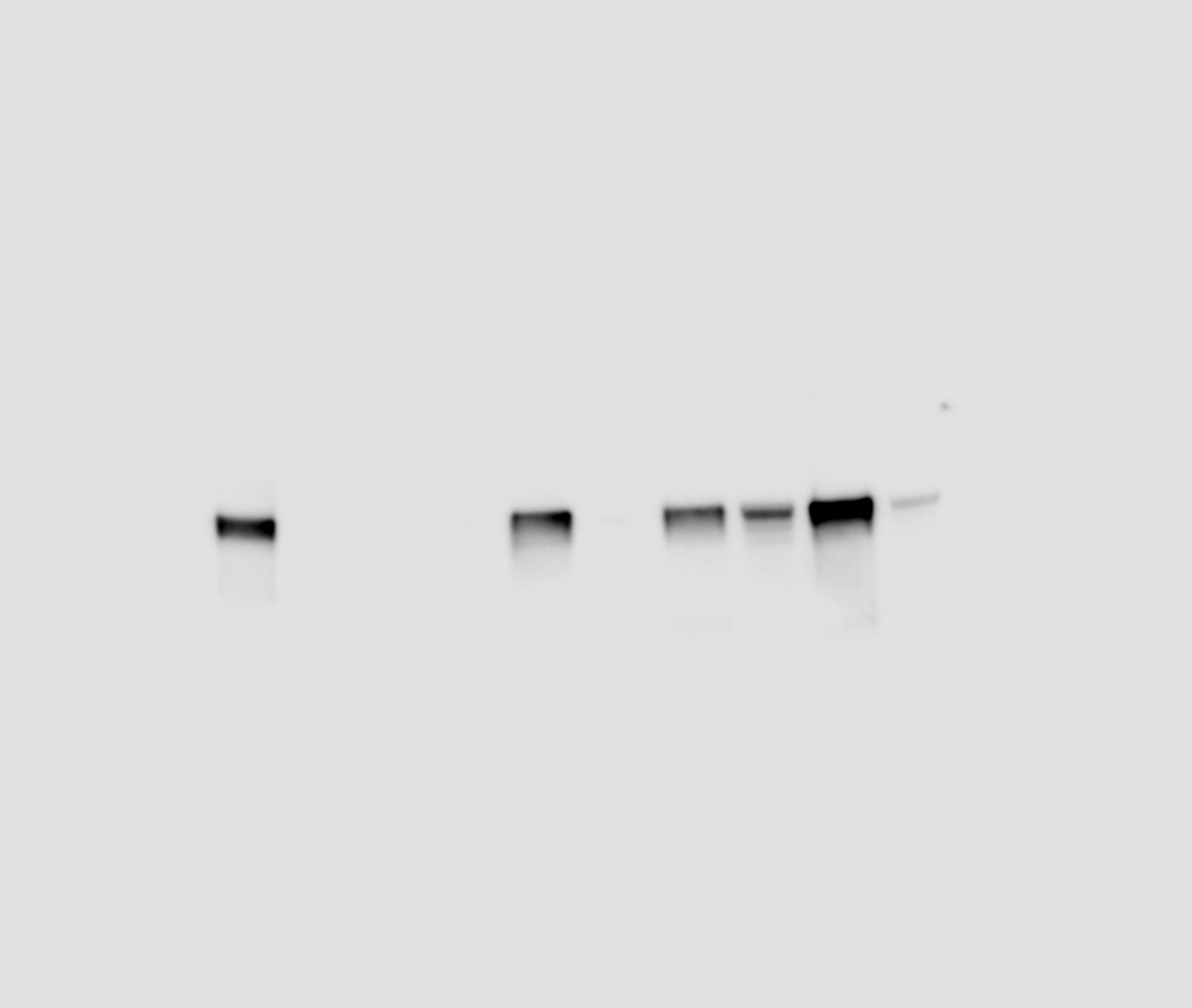

Supplement: Figure 5—source data 1. [file elife-73330-fig5-data1.zip › Figure 5 - Source data 1/Hpylori/BonoboL44Q.png]

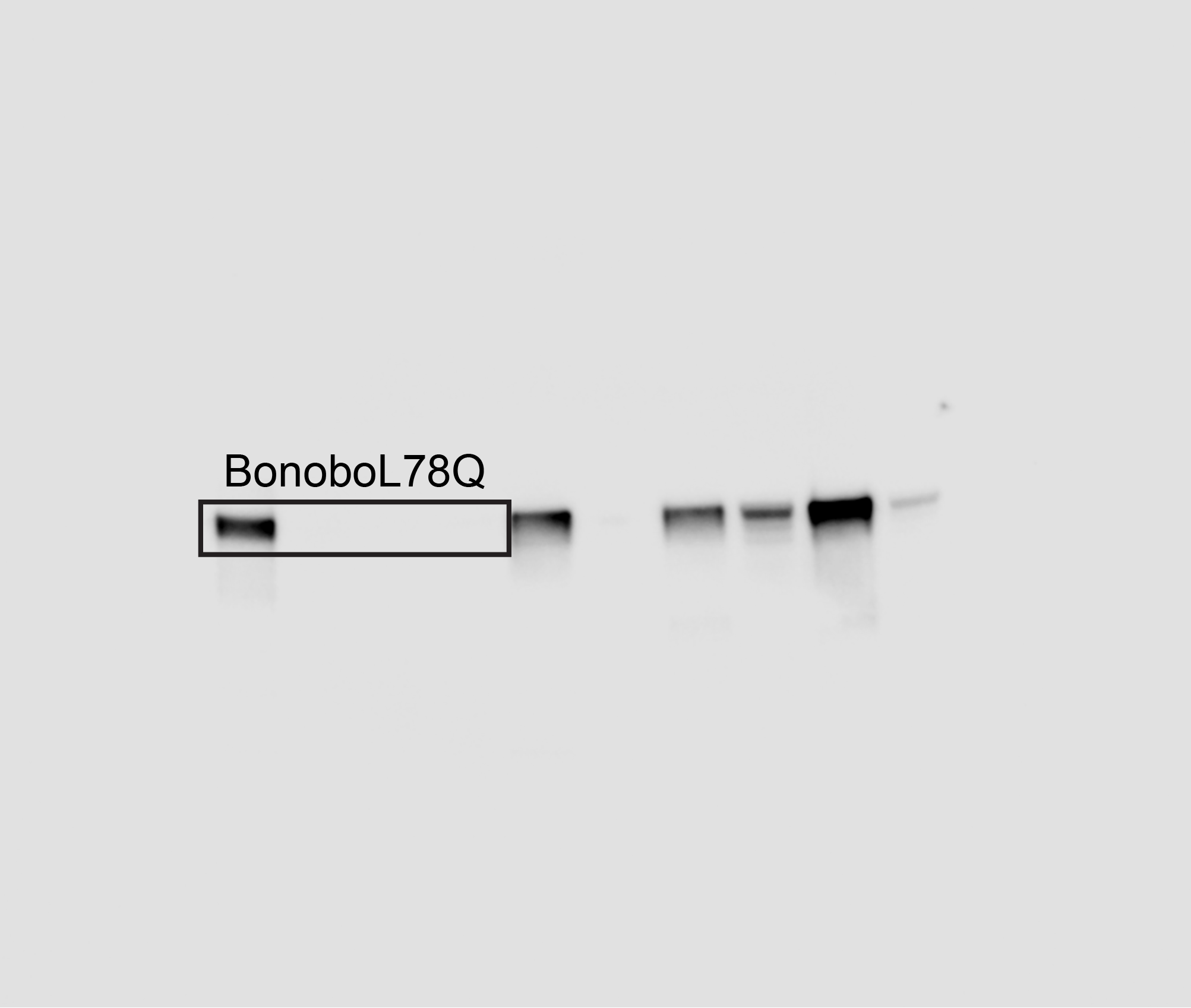

Supplement: Figure 5—source data 1. [file elife-73330-fig5-data1.zip › Figure 5 - Source data 1/Hpylori/BonoboL44Q_label.png]

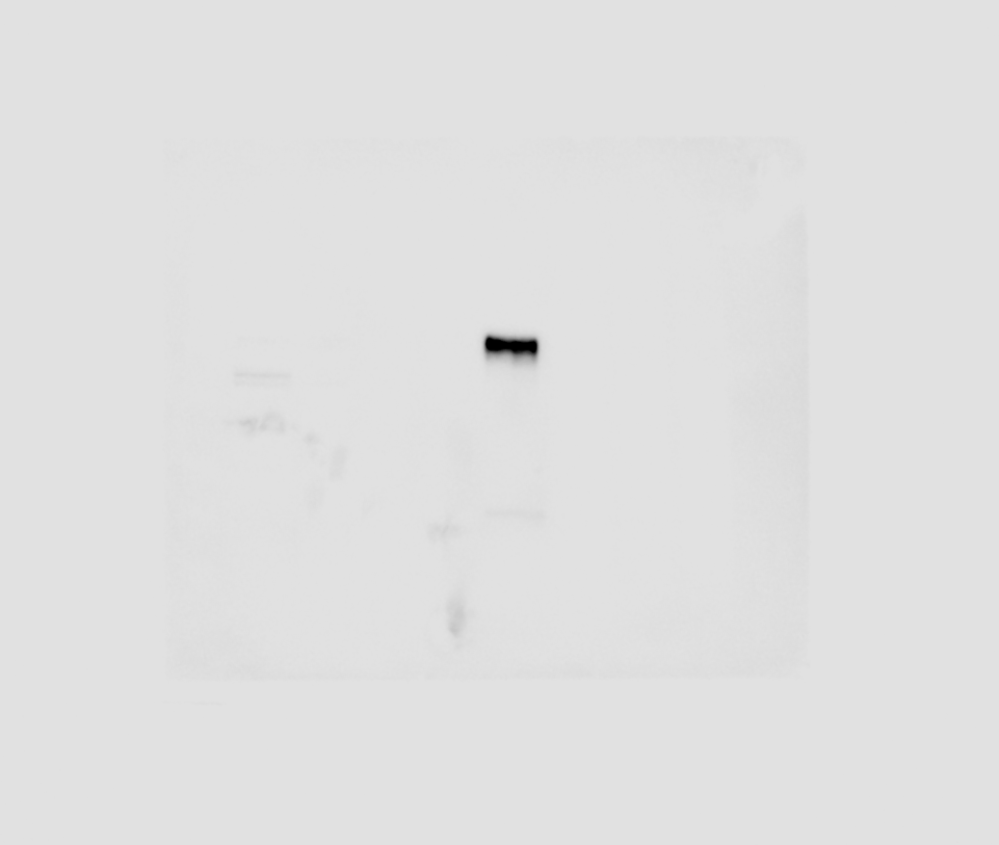

Supplement: Figure 5—source data 1. [file elife-73330-fig5-data1.zip › Figure 5 - Source data 1/Hpylori/BonoboQ51G.png]

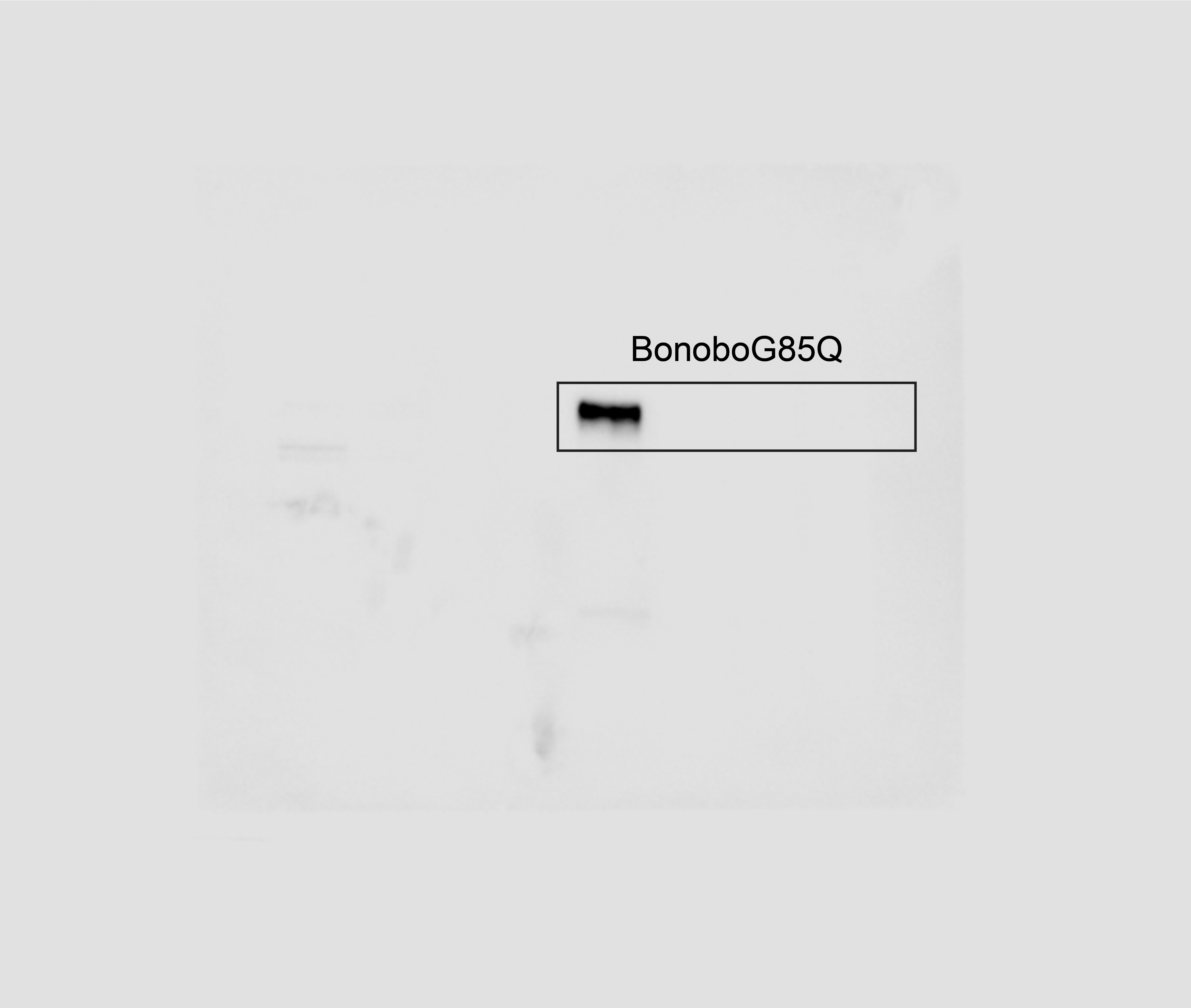

Supplement: Figure 5—source data 1. [file elife-73330-fig5-data1.zip › Figure 5 - Source data 1/Hpylori/BonoboQ51G_label.png]

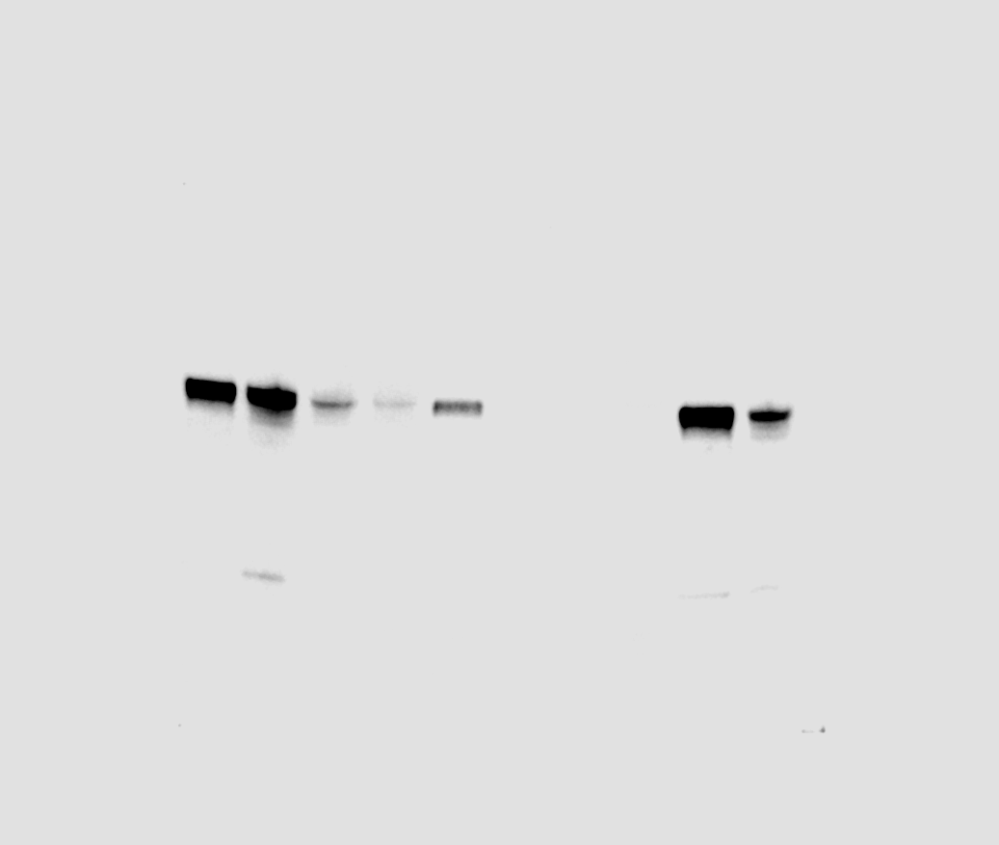

Supplement: Figure 5—source data 1. [file elife-73330-fig5-data1.zip › Figure 5 - Source data 1/Hpylori/BonoboQLF.png]

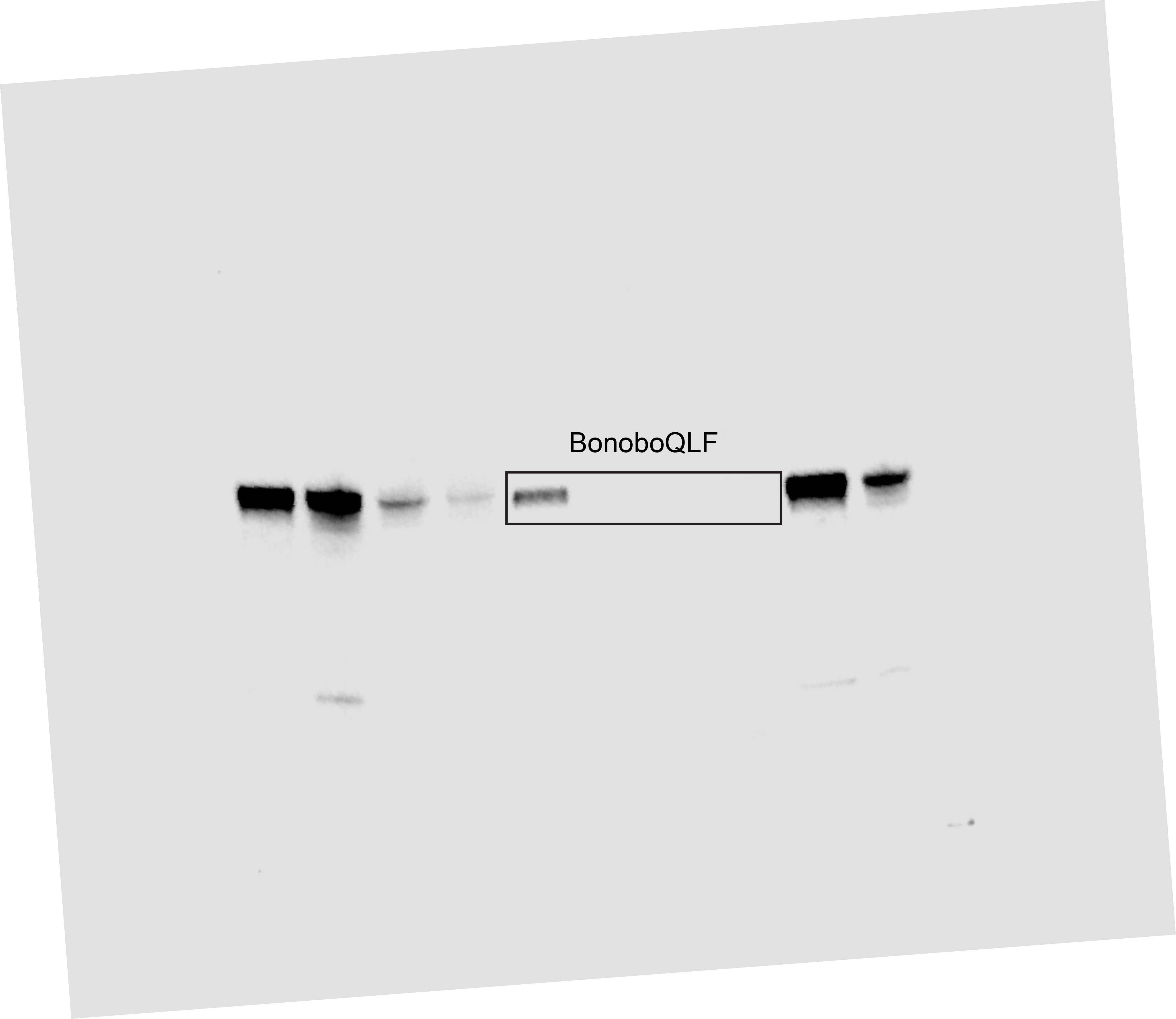

Supplement: Figure 5—source data 1. [file elife-73330-fig5-data1.zip › Figure 5 - Source data 1/Hpylori/BonoboQLF_label.png]

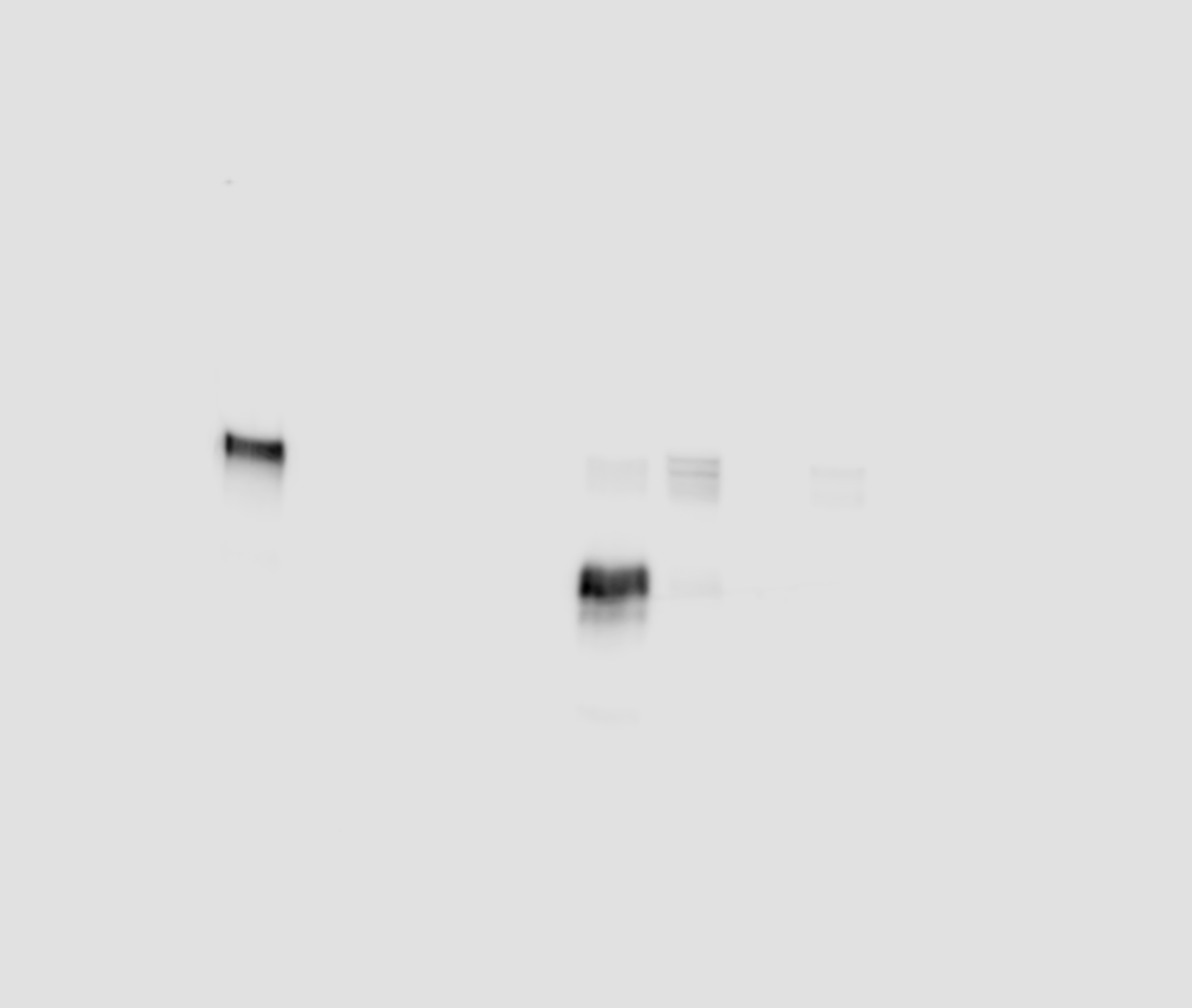

Supplement: Figure 5—source data 1. [file elife-73330-fig5-data1.zip › Figure 5 - Source data 1/Hpylori/BonoboREF.png]

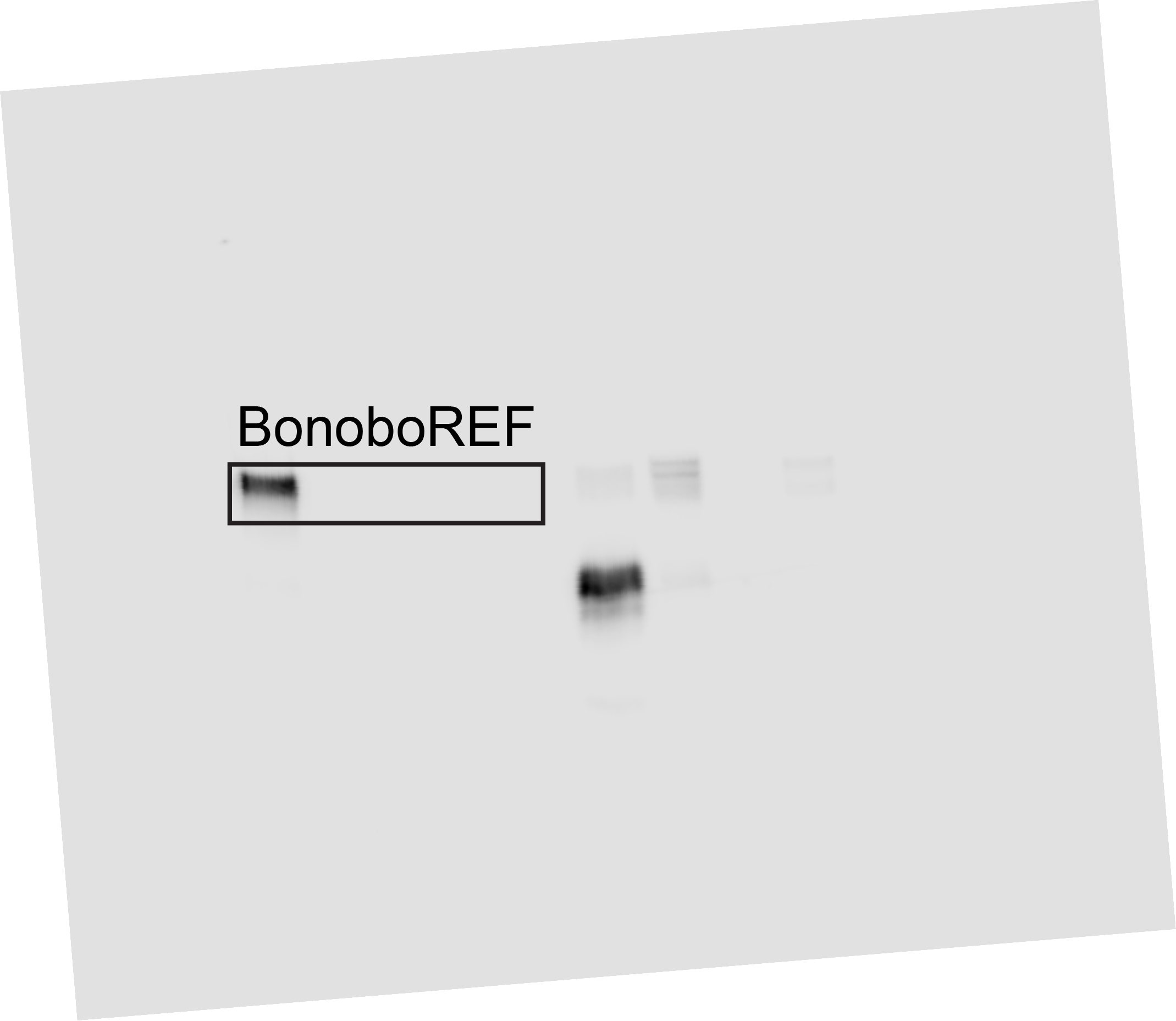

Supplement: Figure 5—source data 1. [file elife-73330-fig5-data1.zip › Figure 5 - Source data 1/Hpylori/BonoboREF_label.png]

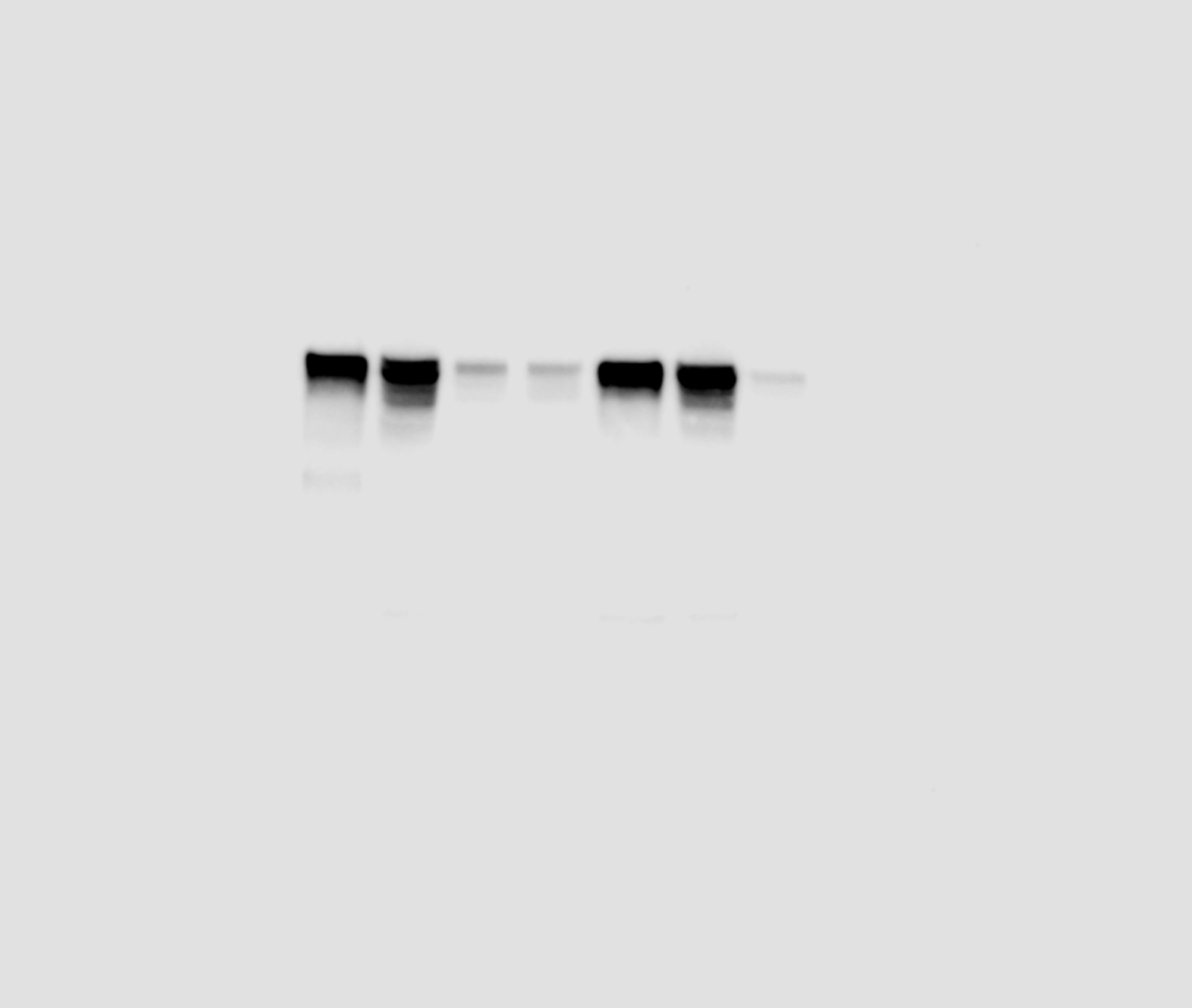

Supplement: Figure 5—source data 1. [file elife-73330-fig5-data1.zip › Figure 5 - Source data 1/Hpylori/HumanG51Q.png]

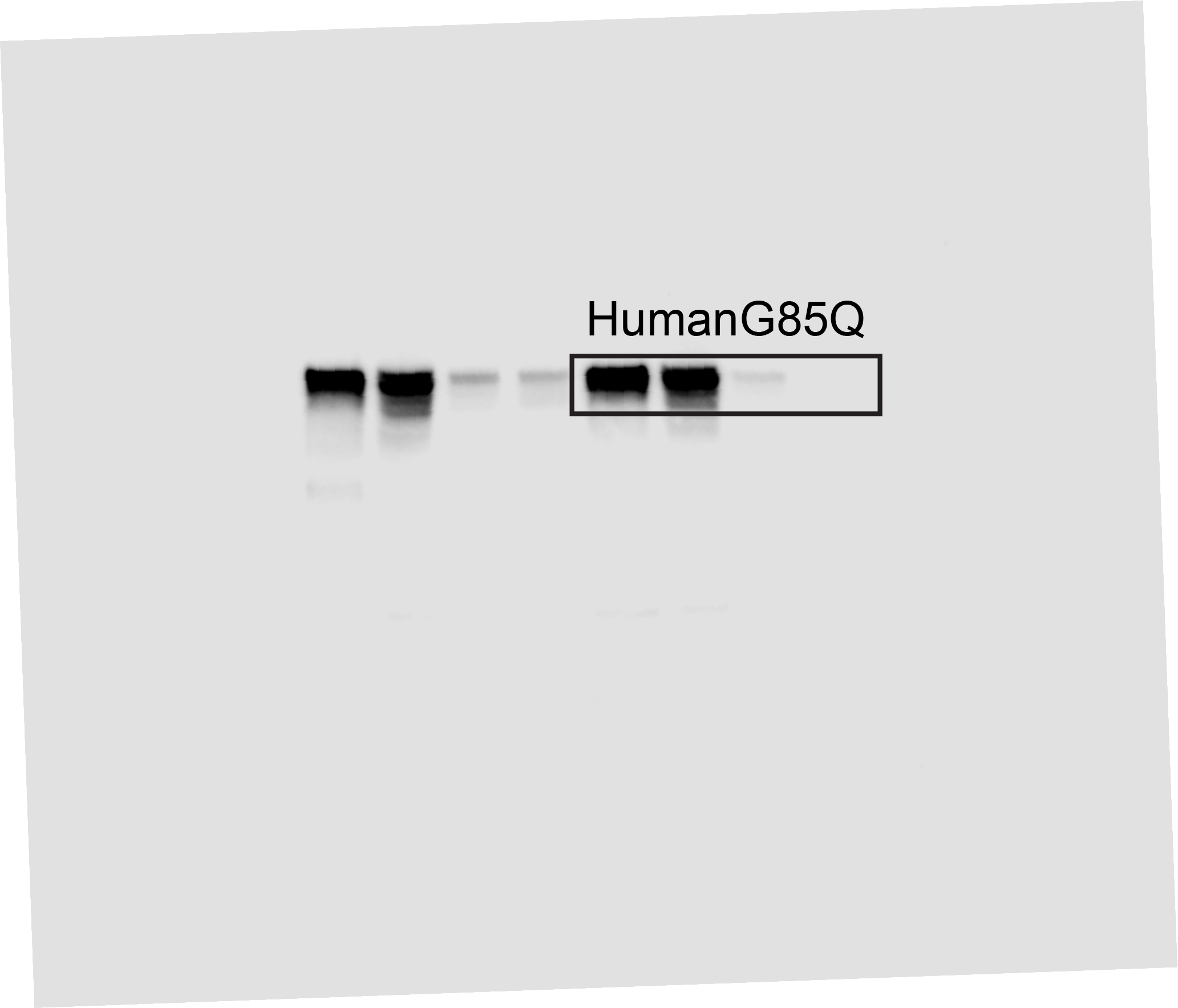

Supplement: Figure 5—source data 1. [file elife-73330-fig5-data1.zip › Figure 5 - Source data 1/Hpylori/HumanG51Q_label.png]

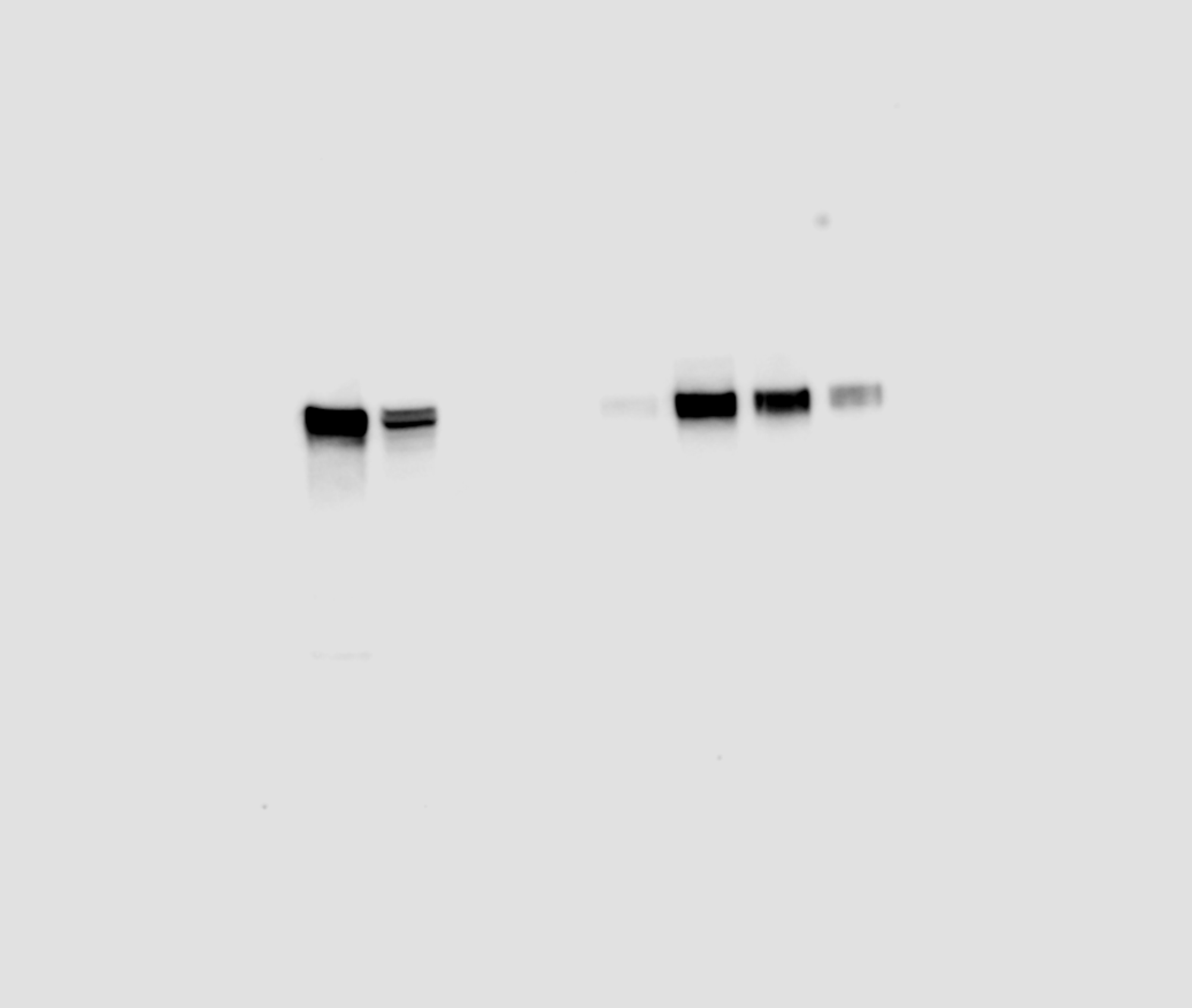

Supplement: Figure 5—source data 1. [file elife-73330-fig5-data1.zip › Figure 5 - Source data 1/Hpylori/HumanNHI.png]

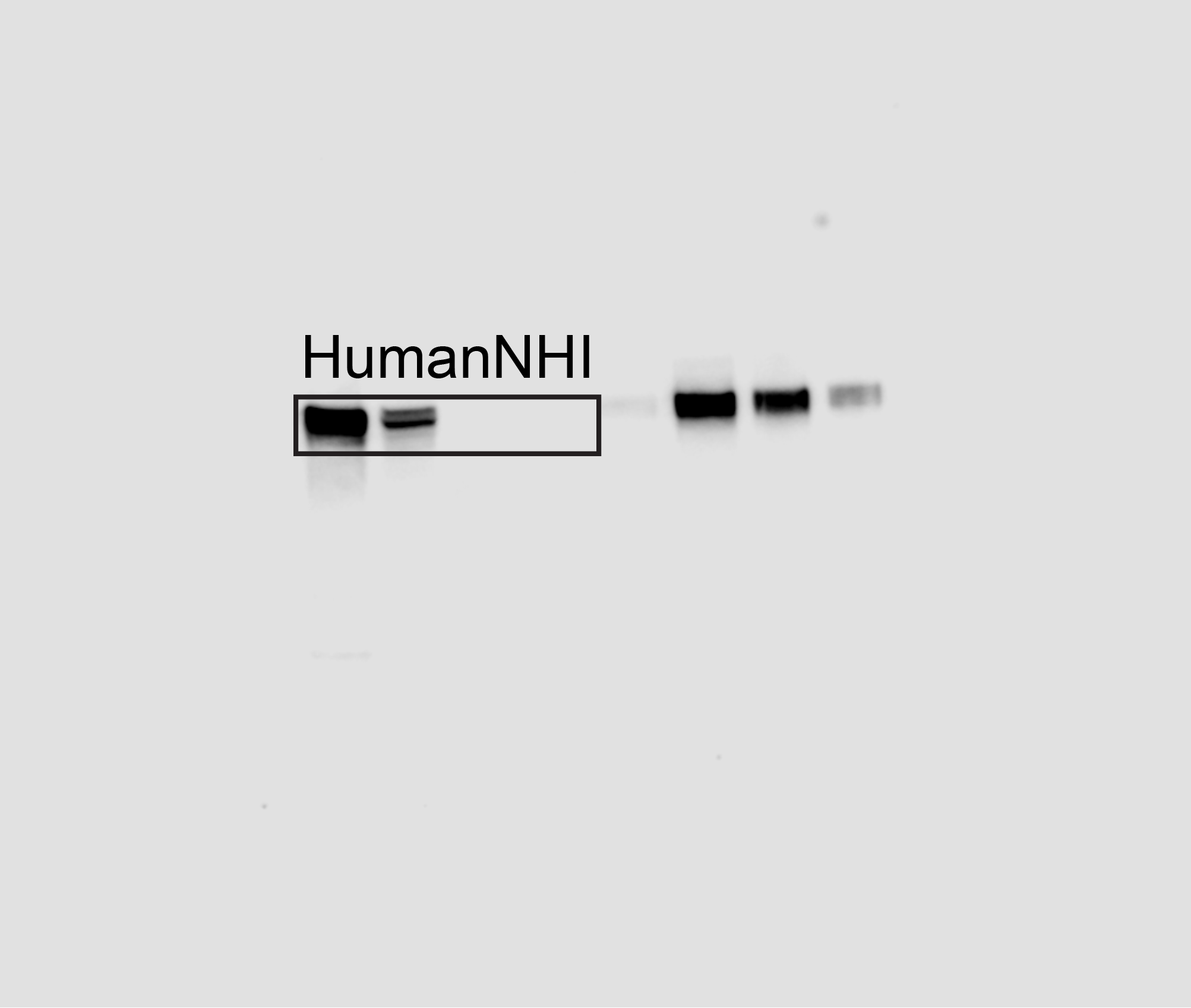

Supplement: Figure 5—source data 1. [file elife-73330-fig5-data1.zip › Figure 5 - Source data 1/Hpylori/HumanNHI_label.png]

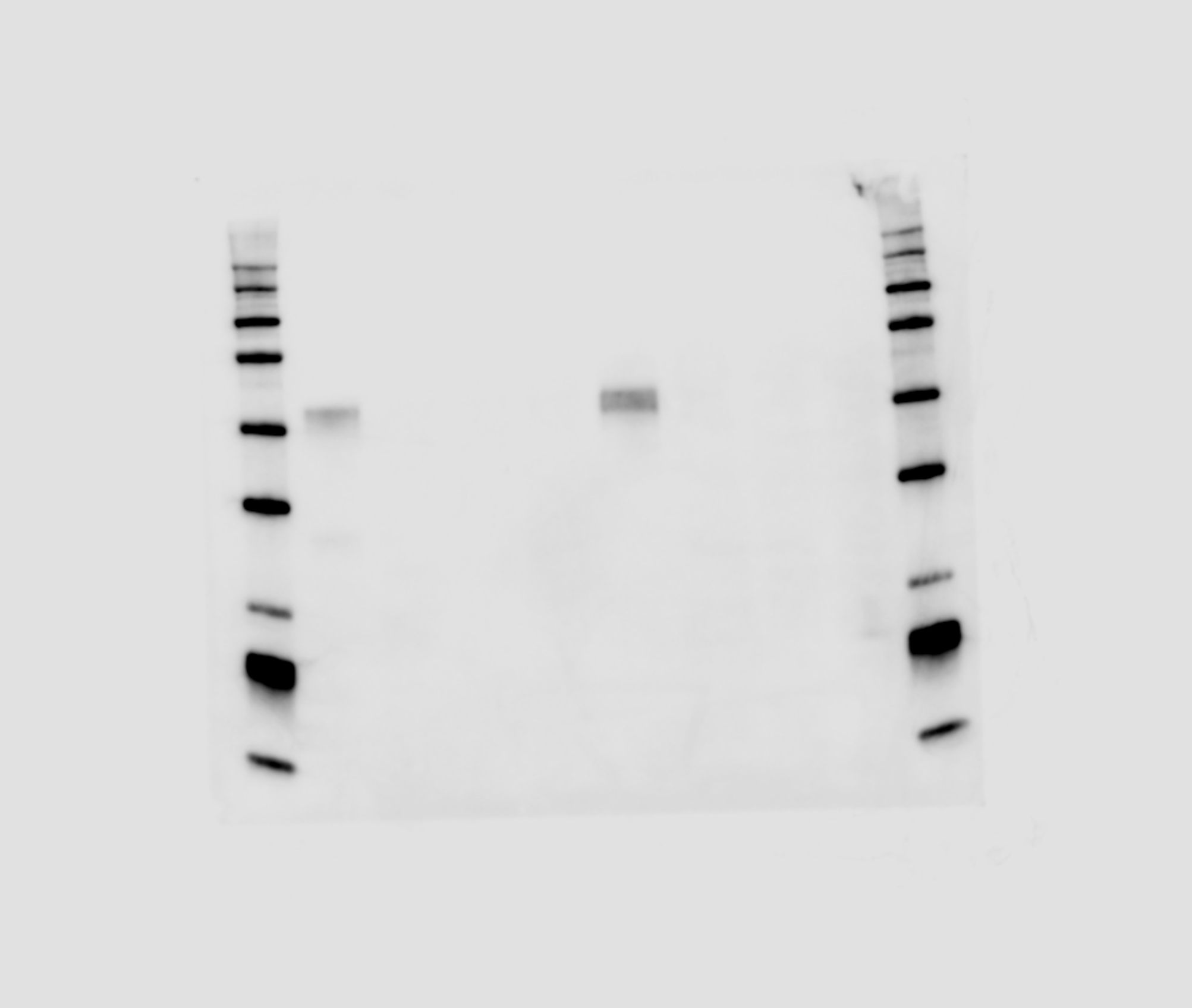

Supplement: Figure 5—source data 1. [file elife-73330-fig5-data1.zip › Figure 5 - Source data 1/Hpylori/HumanQ44L.png]

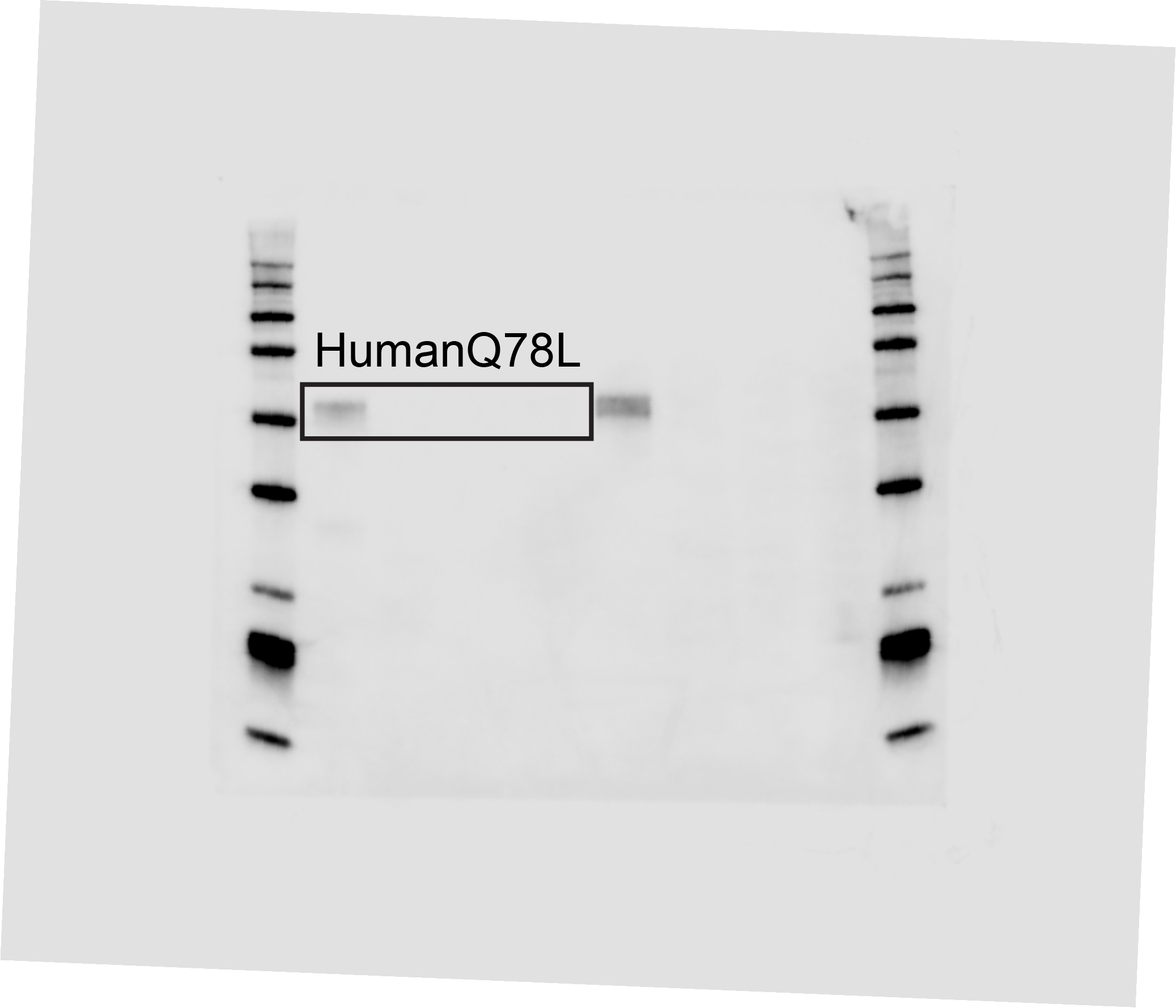

Supplement: Figure 5—source data 1. [file elife-73330-fig5-data1.zip › Figure 5 - Source data 1/Hpylori/HumanQ44L_label.png]

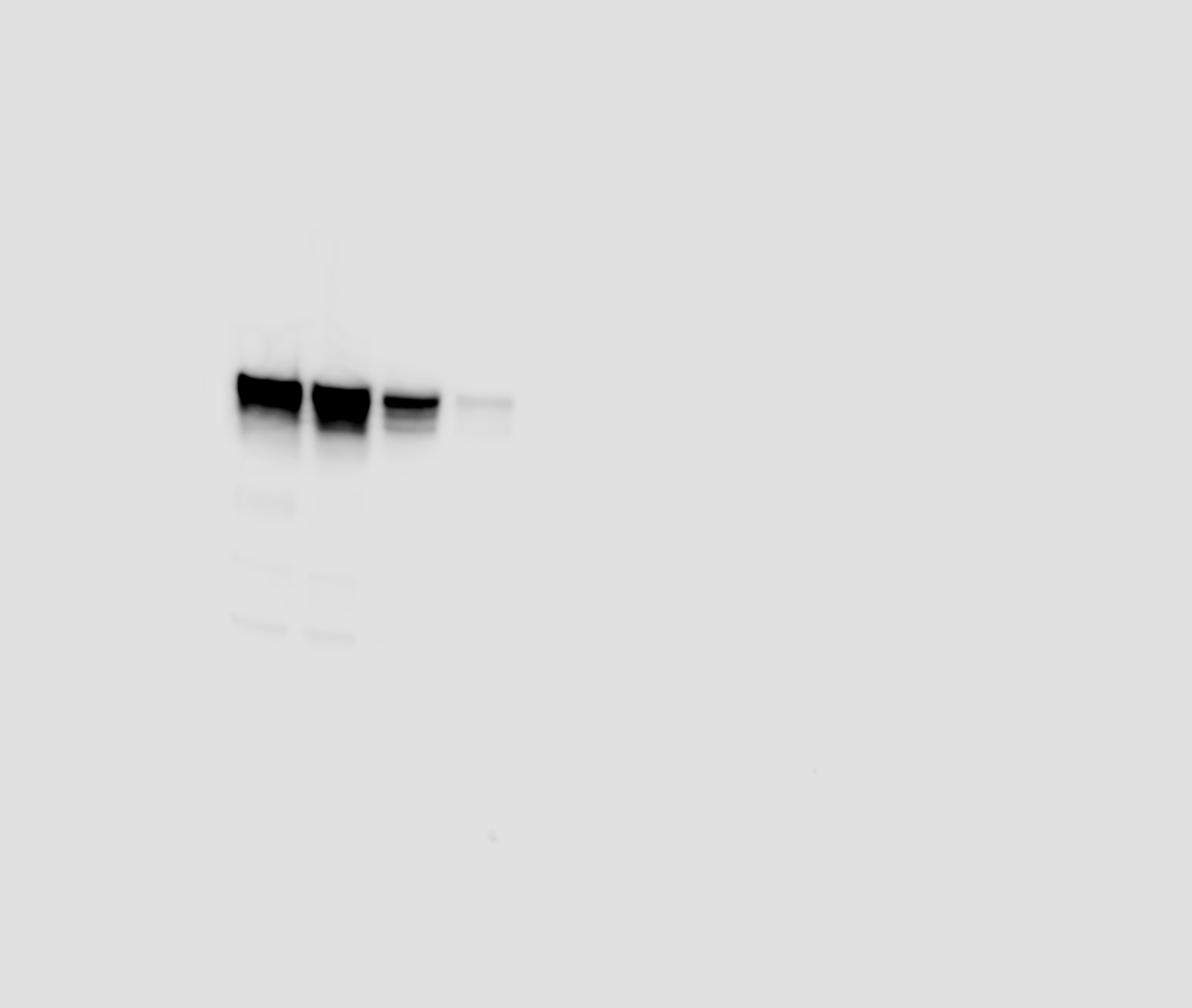

Supplement: Figure 5—source data 1. [file elife-73330-fig5-data1.zip › Figure 5 - Source data 1/Hpylori/HumanREF.png]

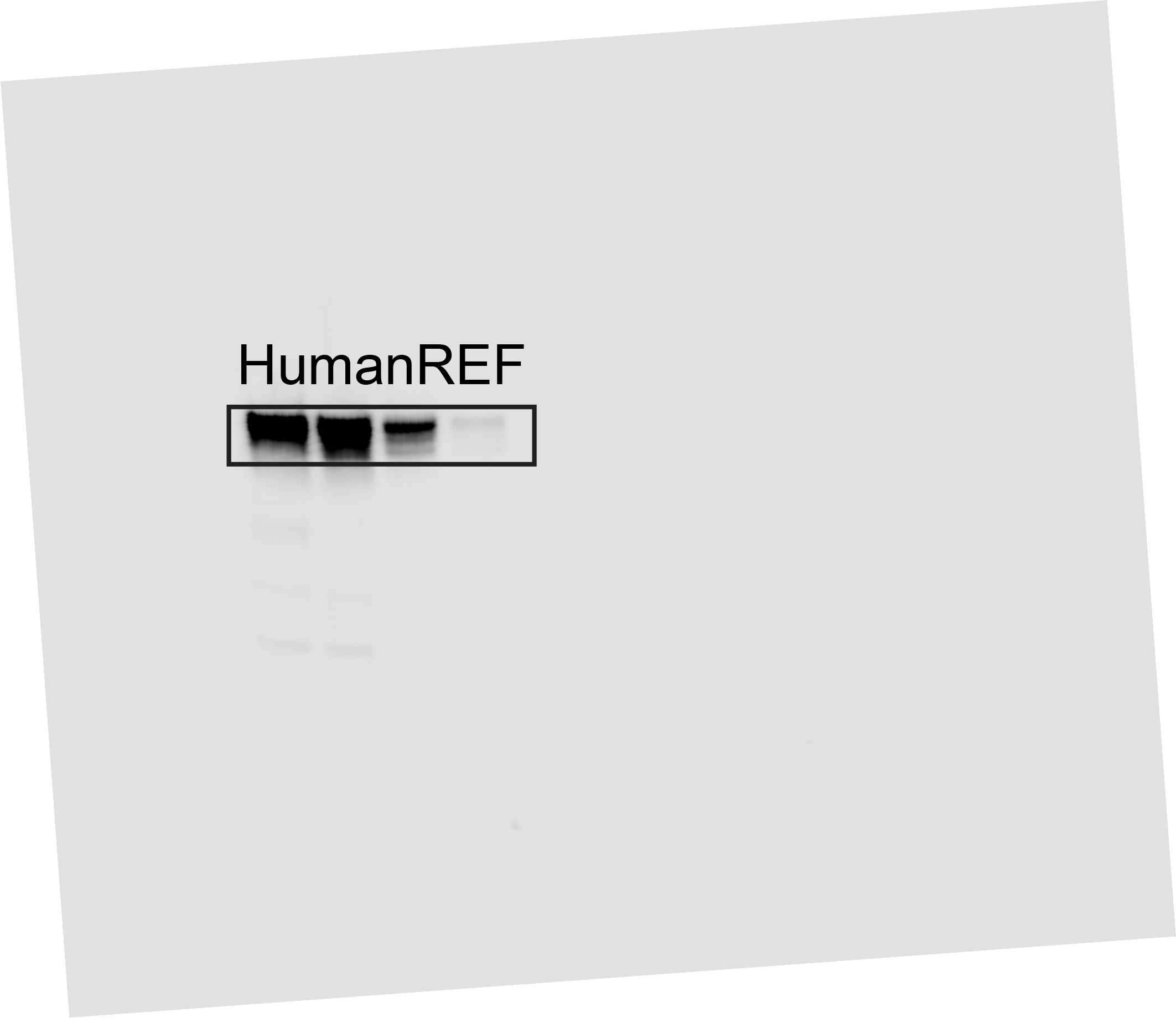

Supplement: Figure 5—source data 1. [file elife-73330-fig5-data1.zip › Figure 5 - Source data 1/Hpylori/HumanREF_label.png]

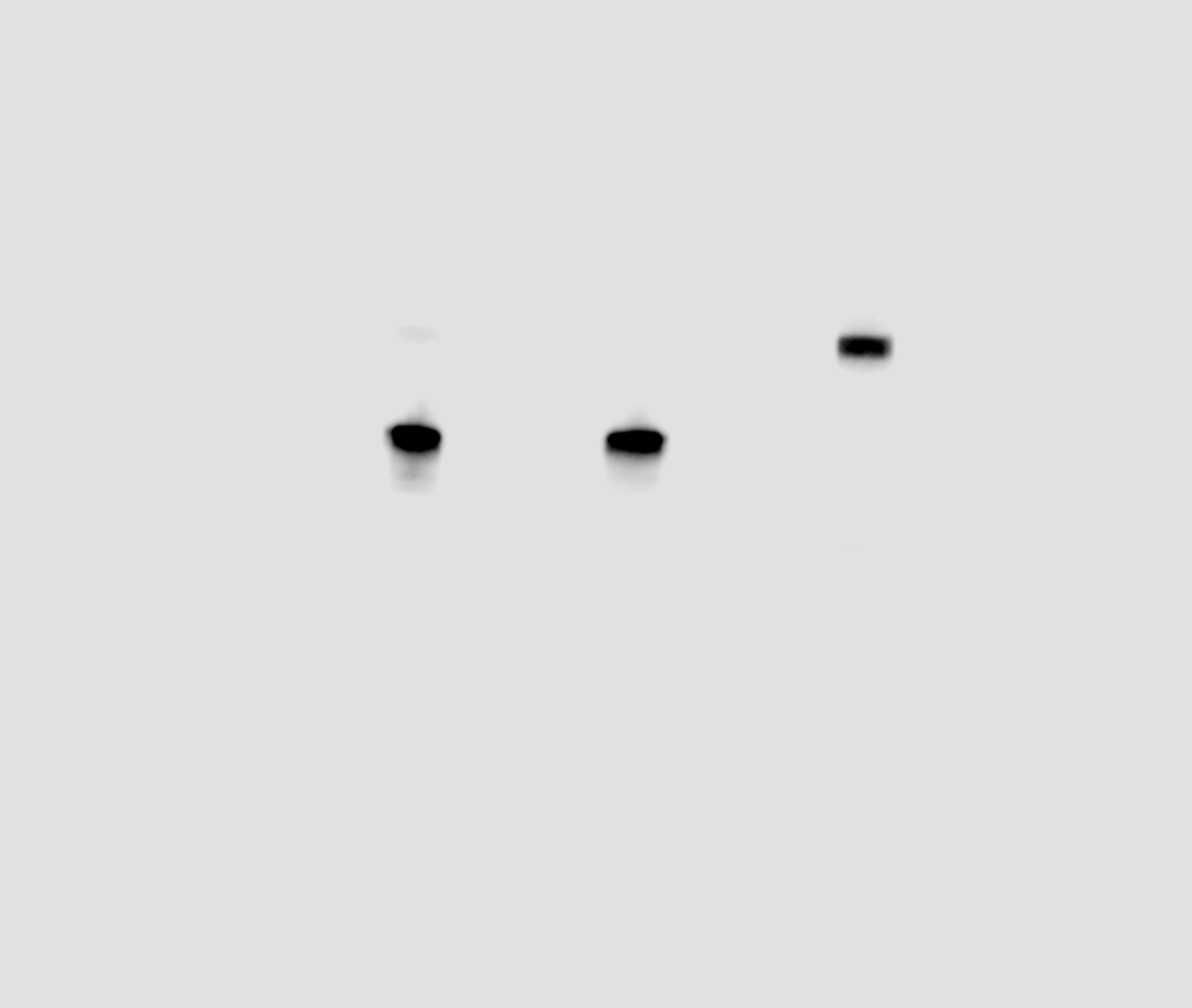

Supplement: Figure 5—source data 1. [file elife-73330-fig5-data1.zip › Figure 5 - Source data 1/Opa/BonoboL44Q.png]

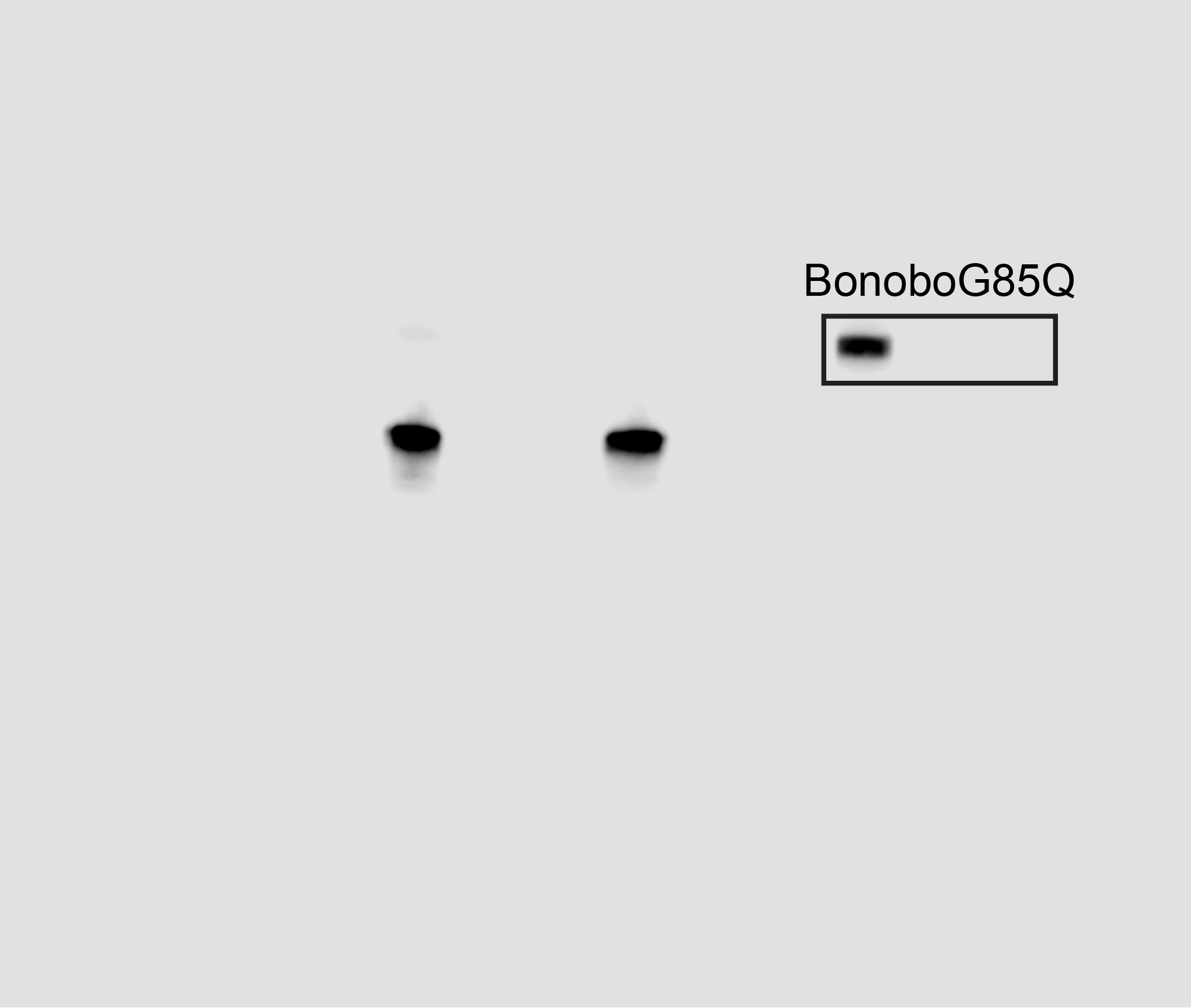

Supplement: Figure 5—source data 1. [file elife-73330-fig5-data1.zip › Figure 5 - Source data 1/Opa/BonoboL44Q_label.png]

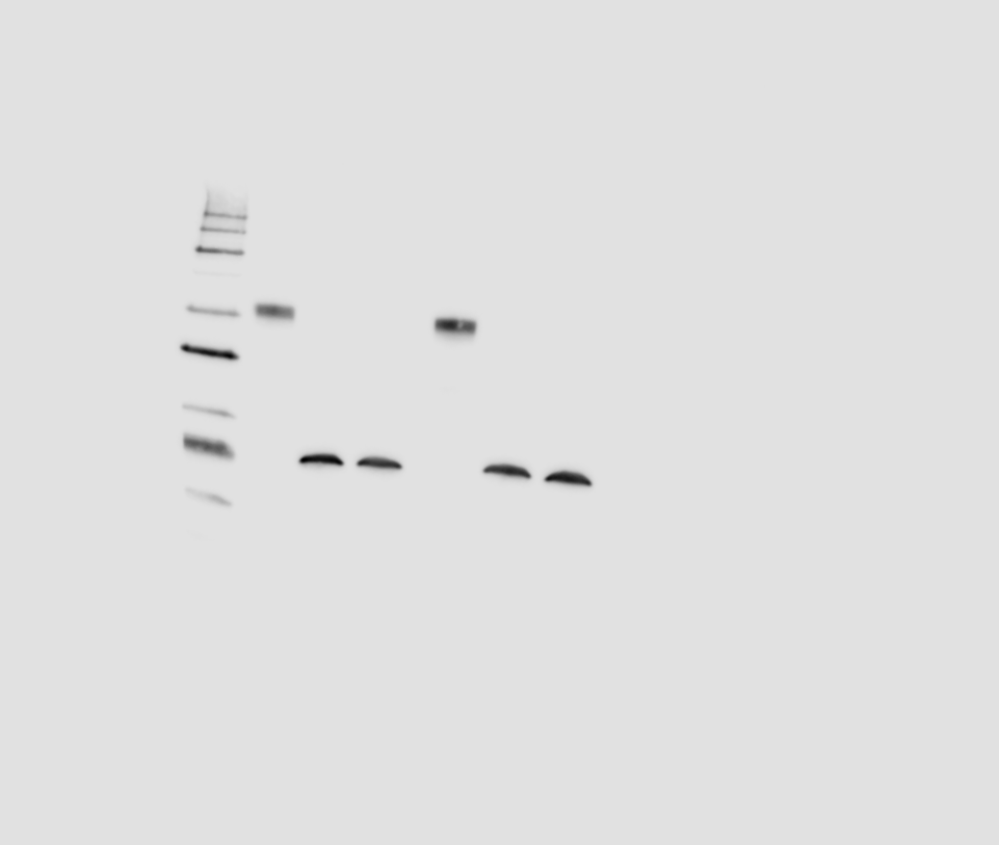

Supplement: Figure 5—source data 1. [file elife-73330-fig5-data1.zip › Figure 5 - Source data 1/Opa/BonoboQ51G&QLF.png]

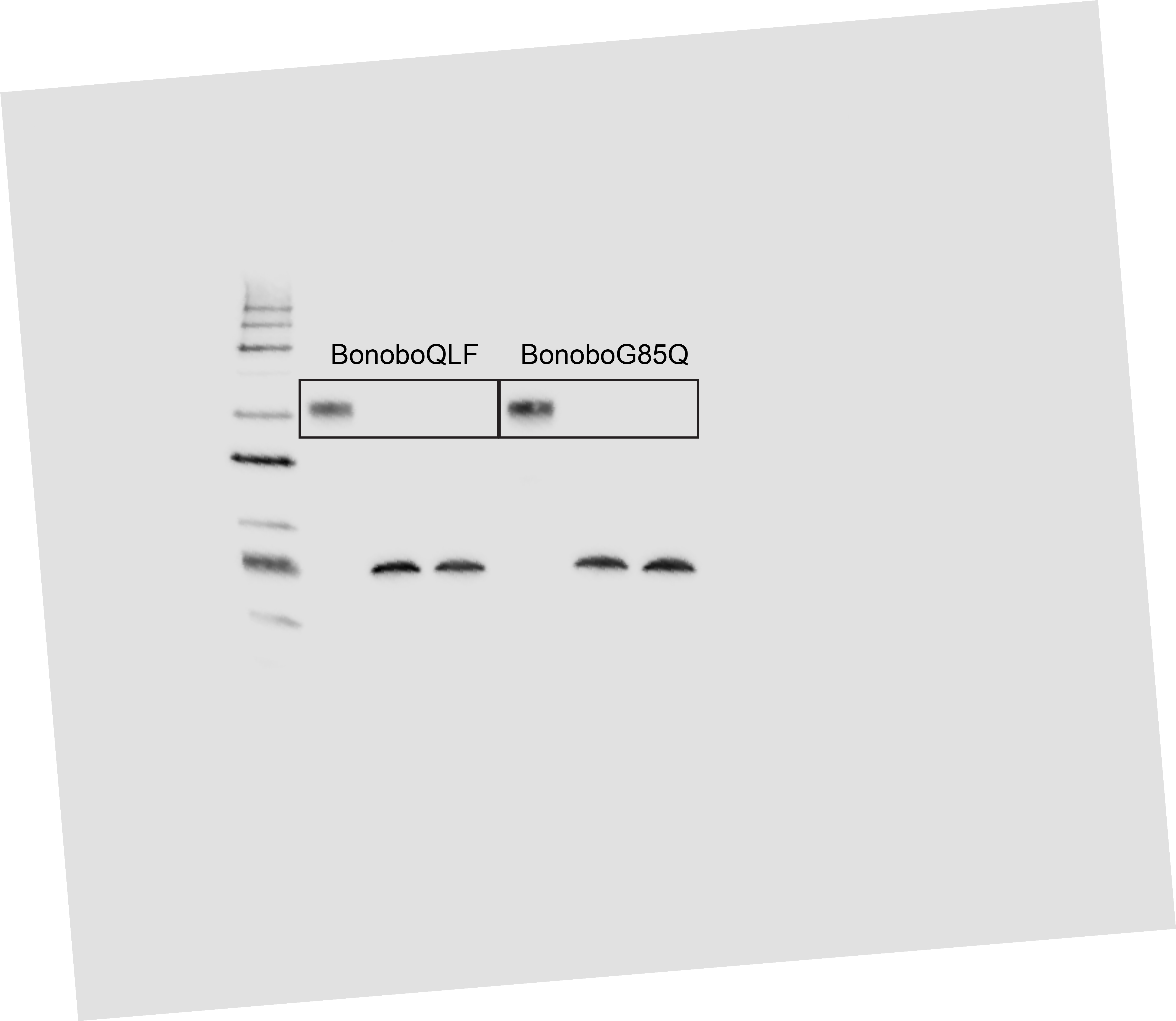

Supplement: Figure 5—source data 1. [file elife-73330-fig5-data1.zip › Figure 5 - Source data 1/Opa/BonoboQ51G&QLF_label.png]

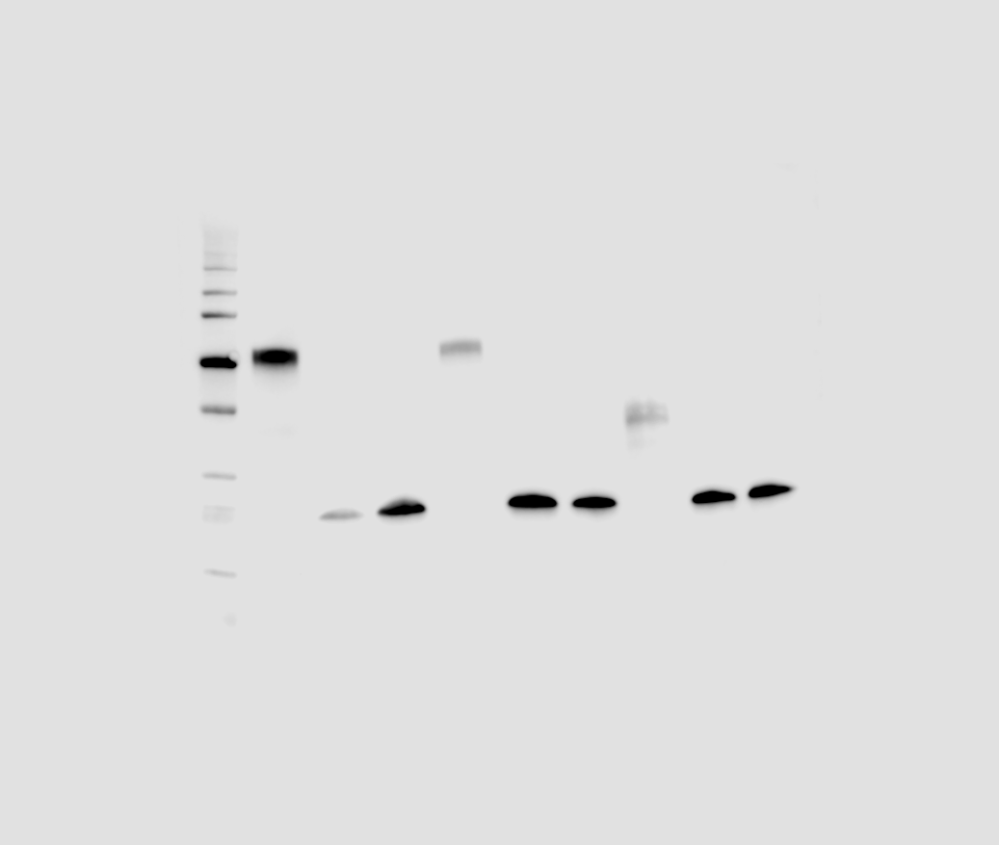

Supplement: Figure 5—source data 1. [file elife-73330-fig5-data1.zip › Figure 5 - Source data 1/Opa/BonoboREF.png]

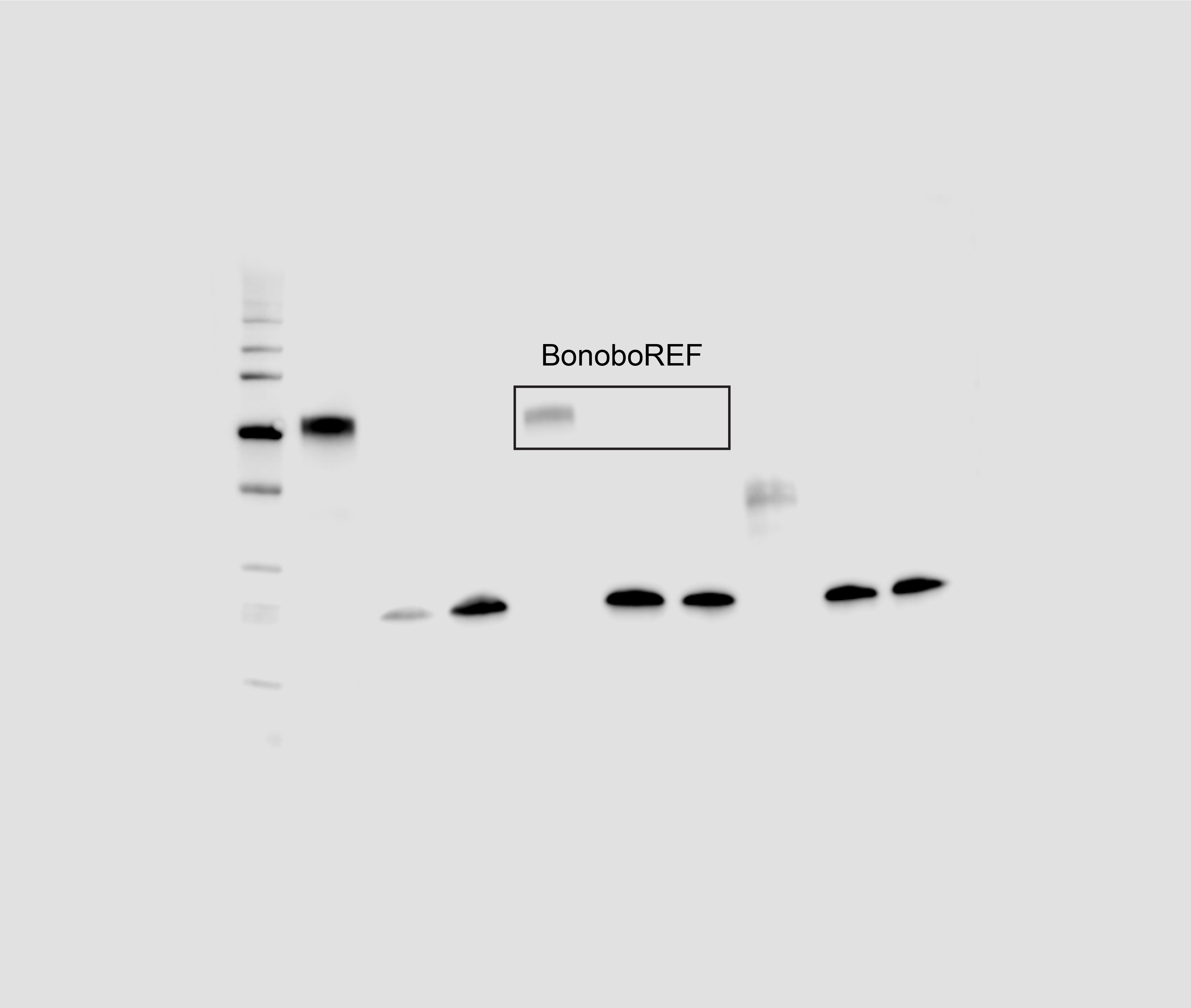

Supplement: Figure 5—source data 1. [file elife-73330-fig5-data1.zip › Figure 5 - Source data 1/Opa/BonoboREF_label.png]

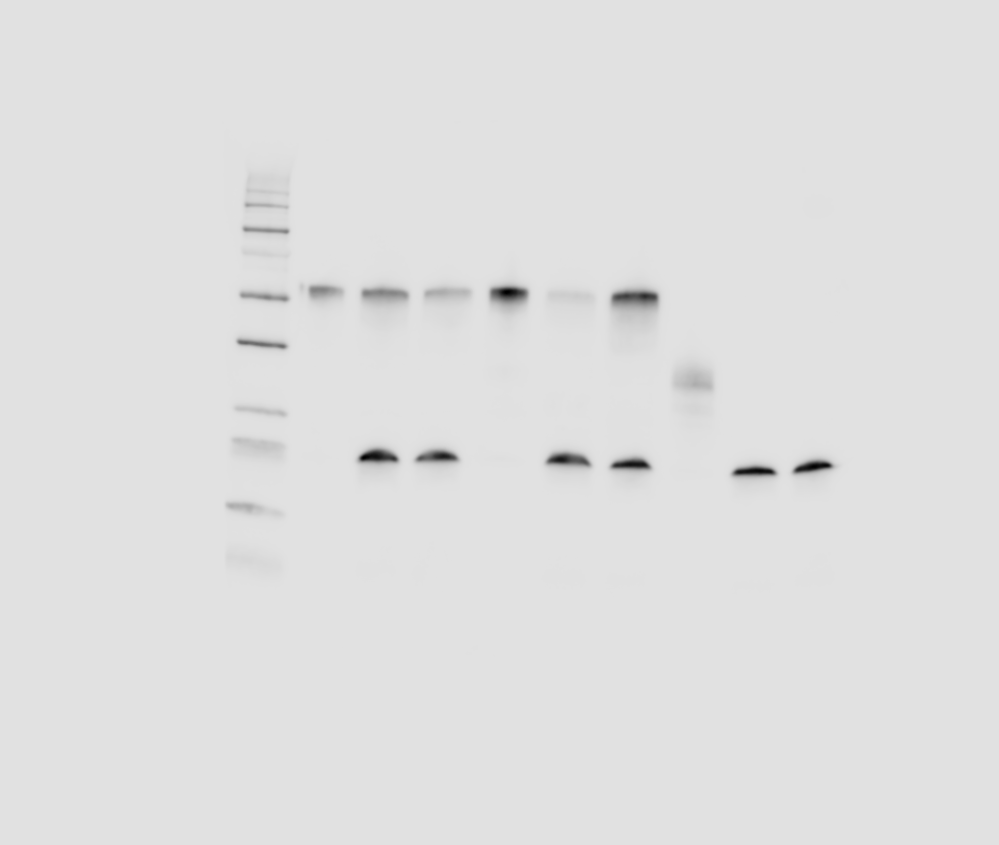

Supplement: Figure 5—source data 1. [file elife-73330-fig5-data1.zip › Figure 5 - Source data 1/Opa/HumanG51Q.png]

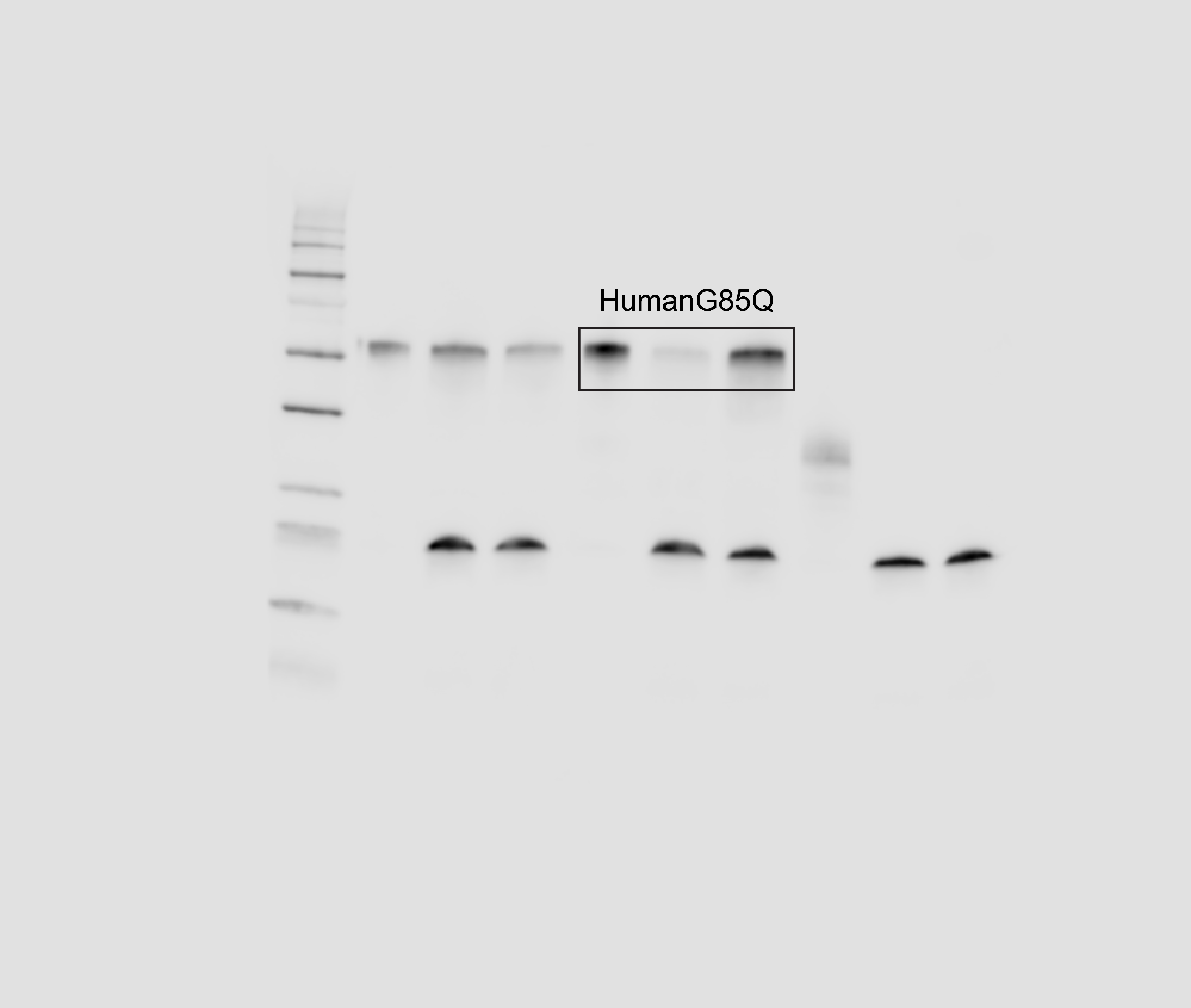

Supplement: Figure 5—source data 1. [file elife-73330-fig5-data1.zip › Figure 5 - Source data 1/Opa/HumanG51Q_label.png]

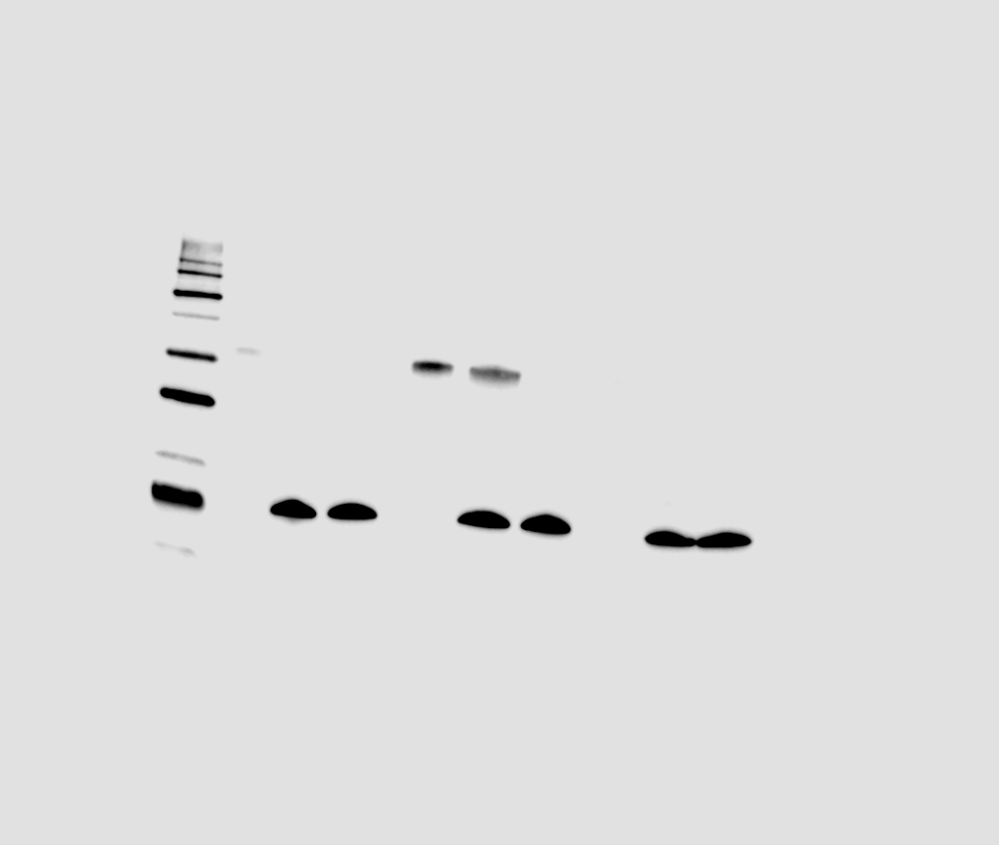

Supplement: Figure 5—source data 1. [file elife-73330-fig5-data1.zip › Figure 5 - Source data 1/Opa/HumanNHI.png]

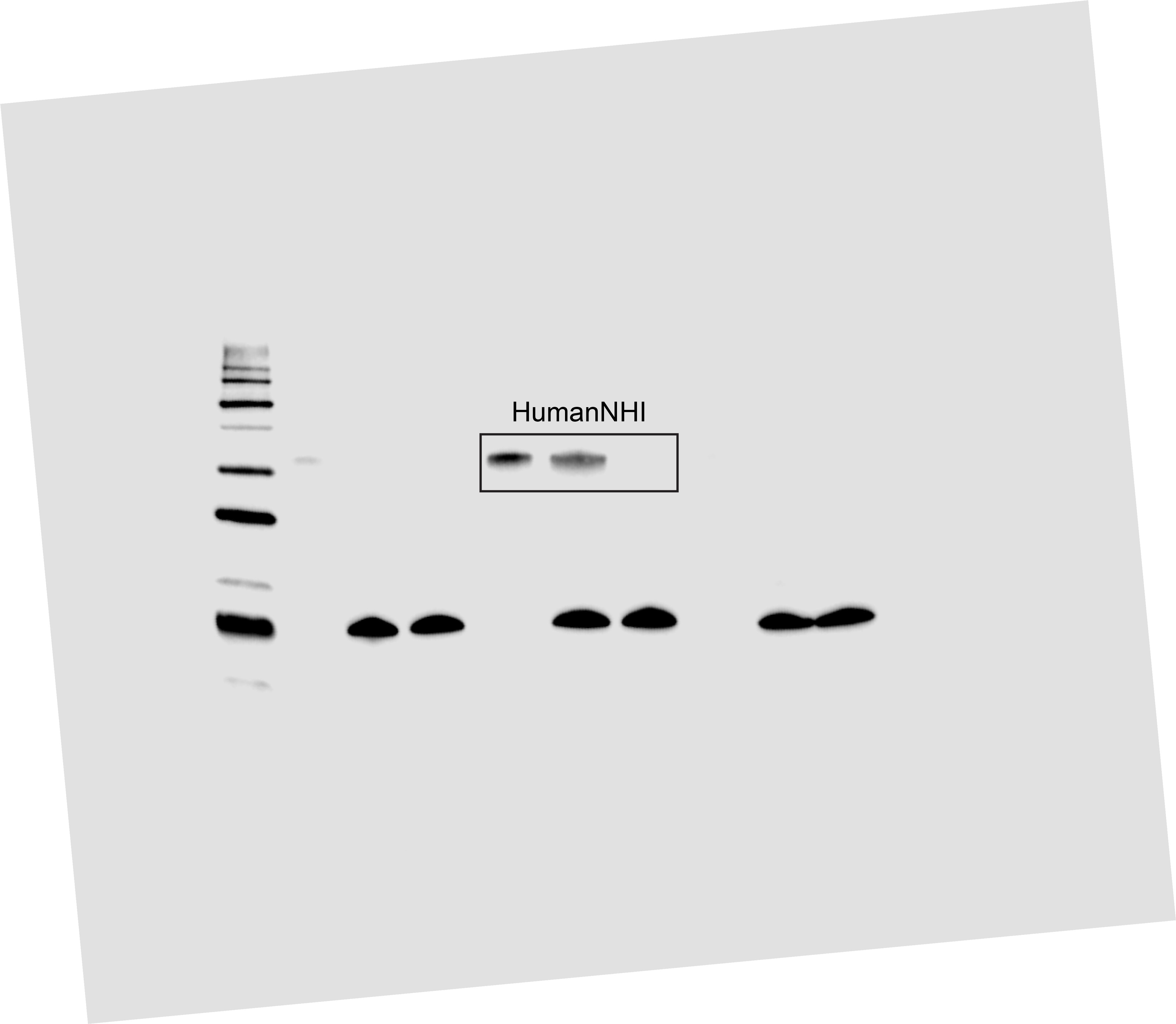

Supplement: Figure 5—source data 1. [file elife-73330-fig5-data1.zip › Figure 5 - Source data 1/Opa/HumanNHI_label.png]

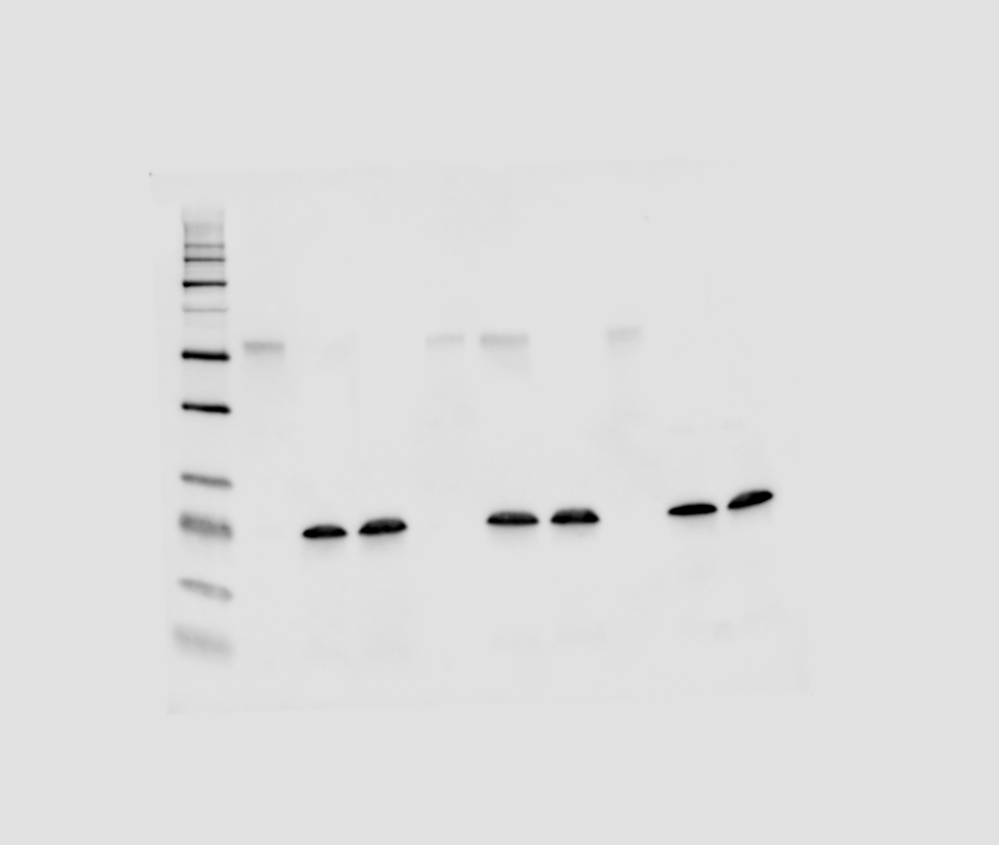

Supplement: Figure 5—source data 1. [file elife-73330-fig5-data1.zip › Figure 5 - Source data 1/Opa/HumanQ44L.png]

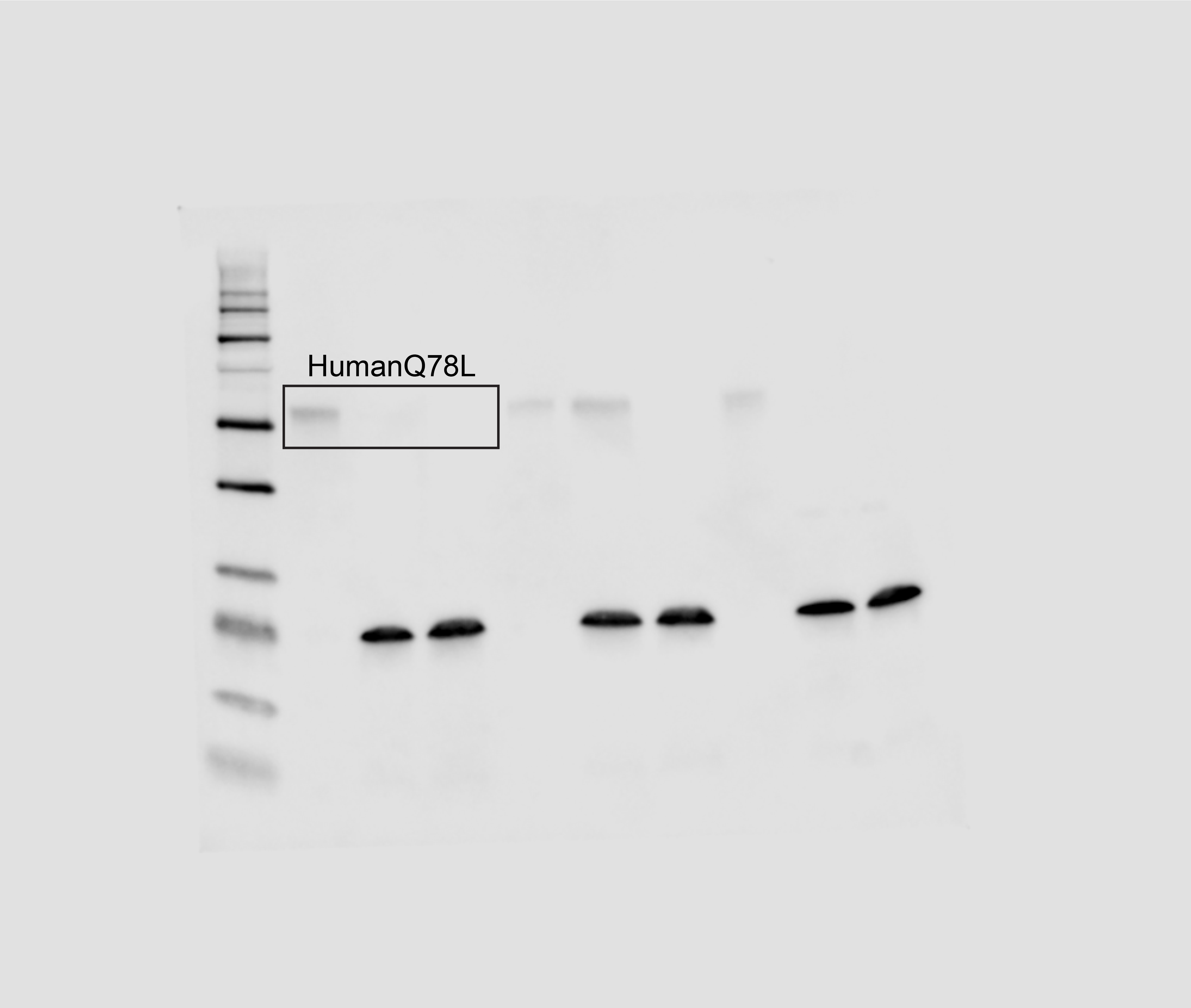

Supplement: Figure 5—source data 1. [file elife-73330-fig5-data1.zip › Figure 5 - Source data 1/Opa/HumanQ44L_label.png]

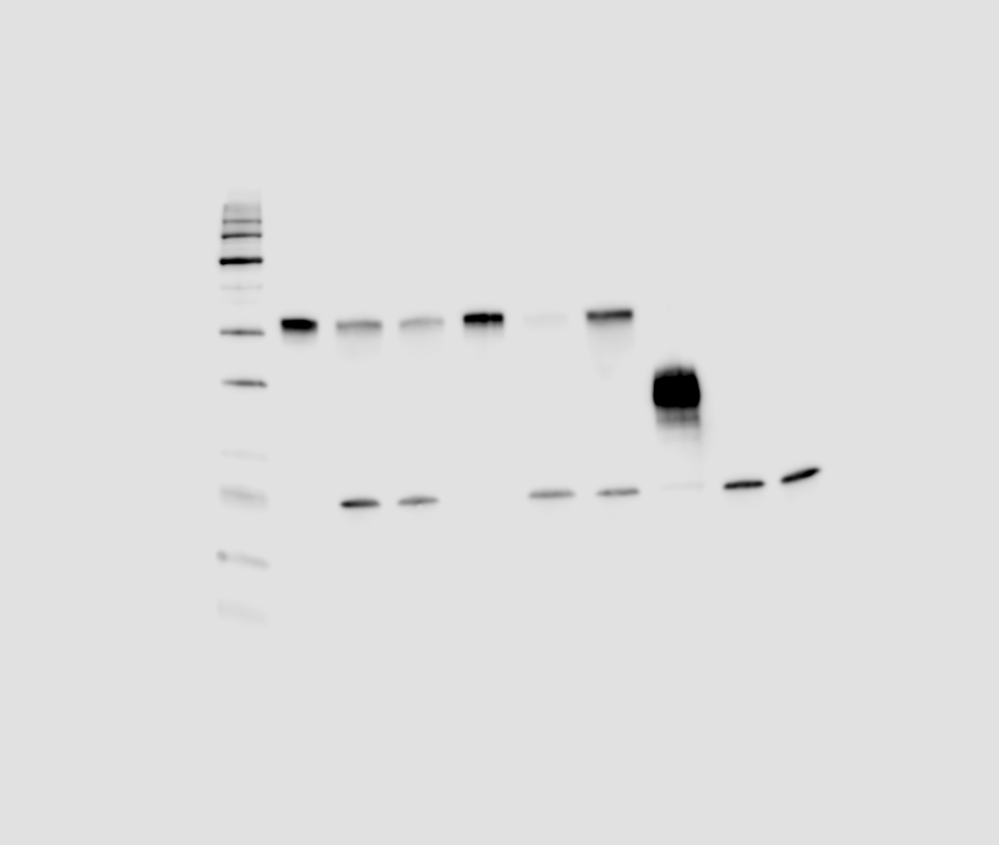

Supplement: Figure 5—source data 1. [file elife-73330-fig5-data1.zip › Figure 5 - Source data 1/Opa/HumanREF.png]

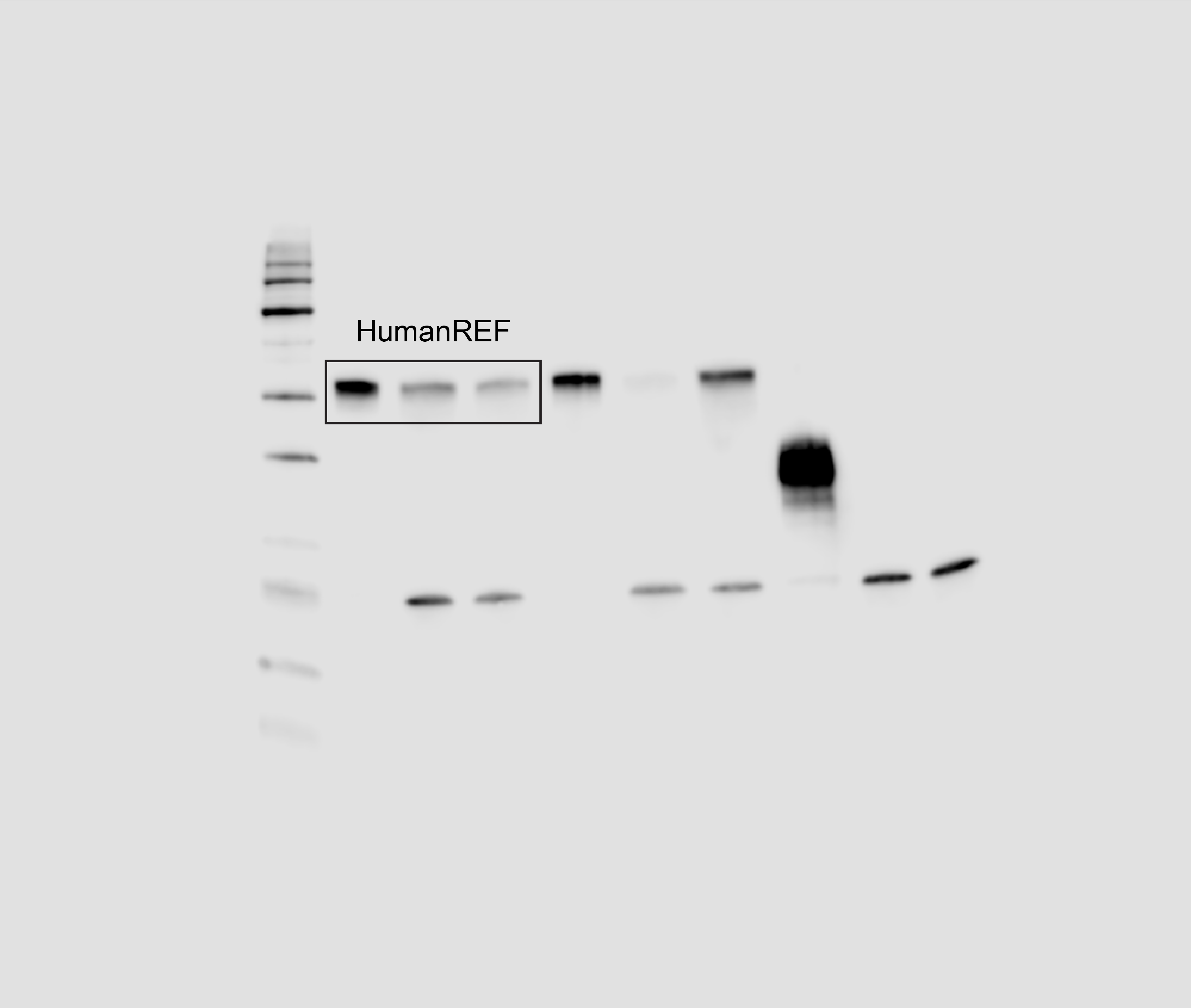

Supplement: Figure 5—source data 1. [file elife-73330-fig5-data1.zip › Figure 5 - Source data 1/Opa/HumanREF_label.png]

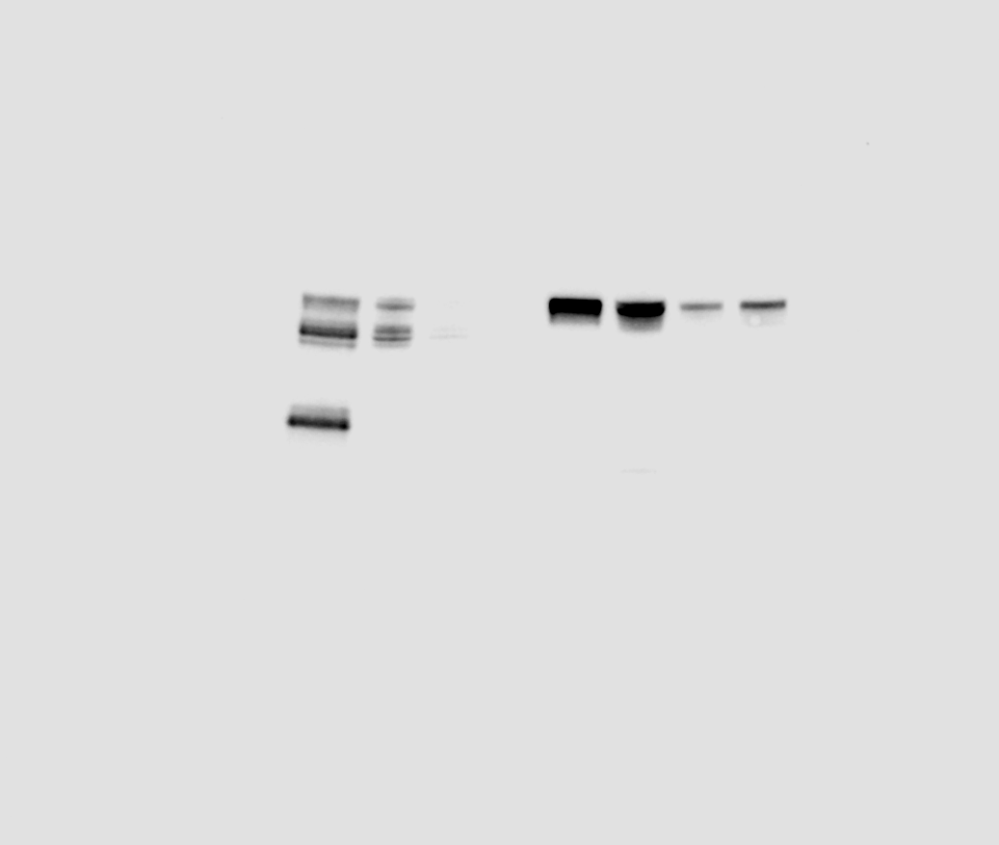

Supplement: Figure 6—source data 2. [file elife-73330-fig6-data2.zip › Figure 6 - Source data 2/Hpylori/HumanA49V,Q89H.png]

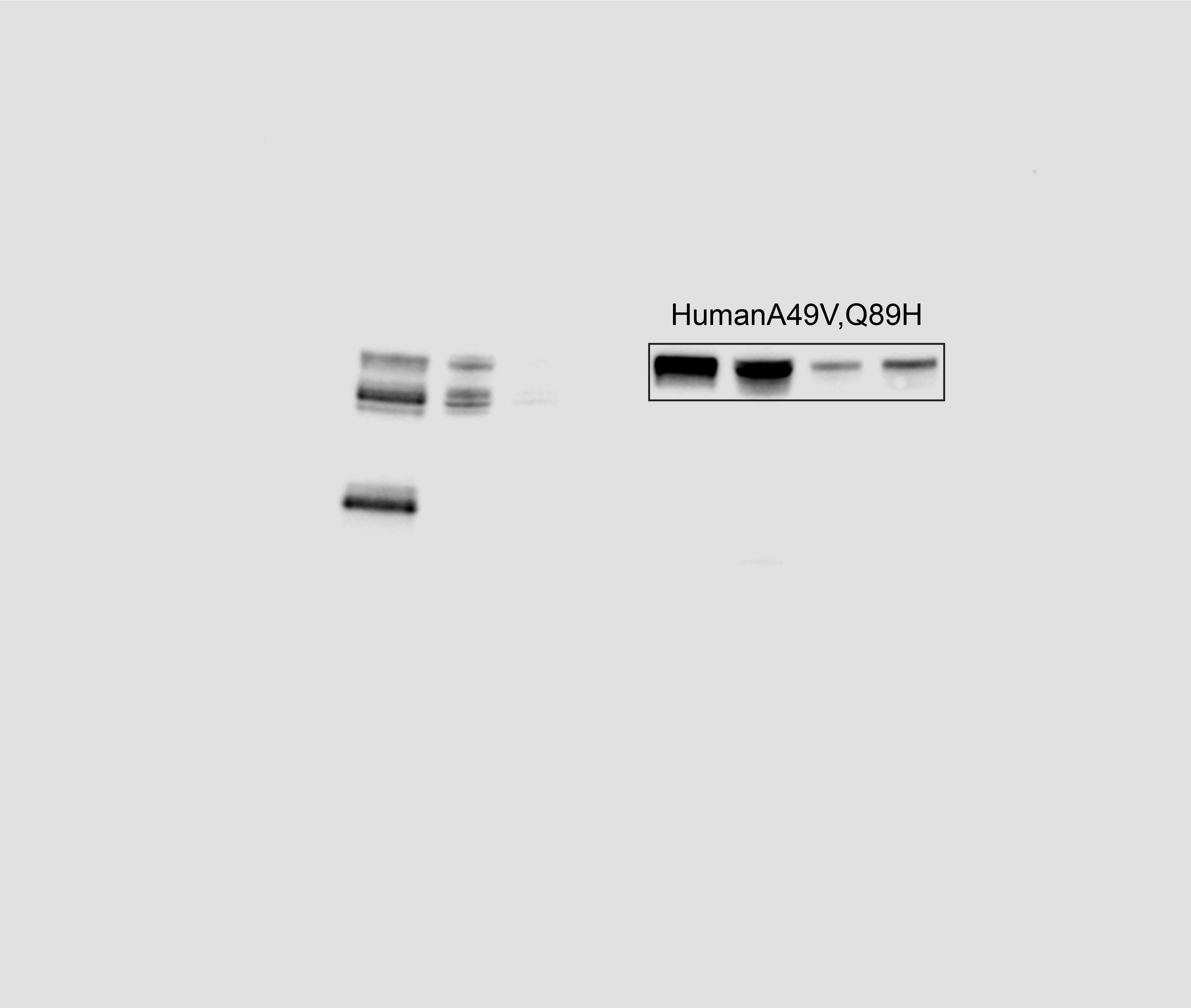

Supplement: Figure 6—source data 2. [file elife-73330-fig6-data2.zip › Figure 6 - Source data 2/Hpylori/HumanA49V,Q89H_label.png]

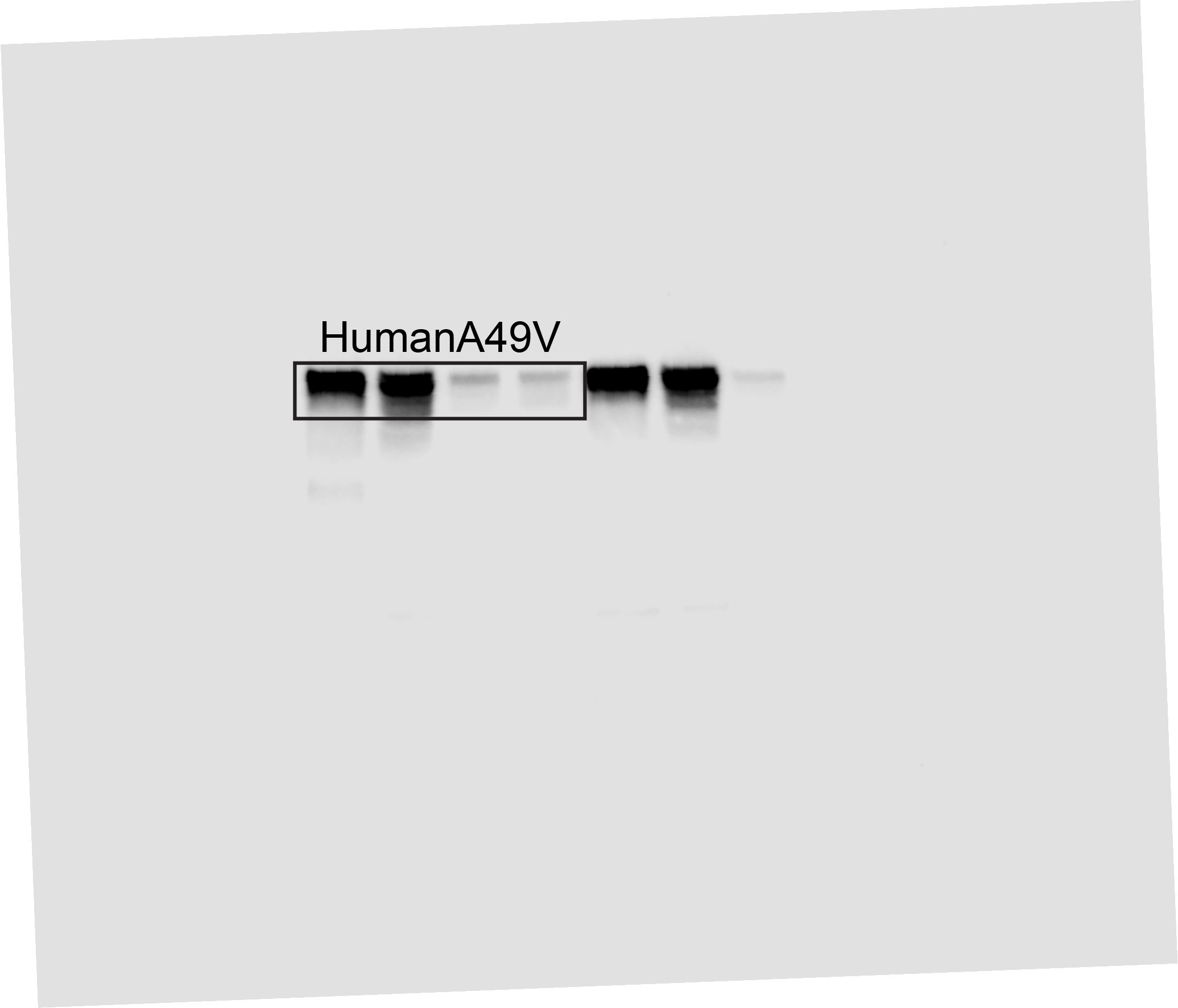

Supplement: Figure 6—source data 2. [file elife-73330-fig6-data2.zip › Figure 6 - Source data 2/Hpylori/HumanA49V_label.png]

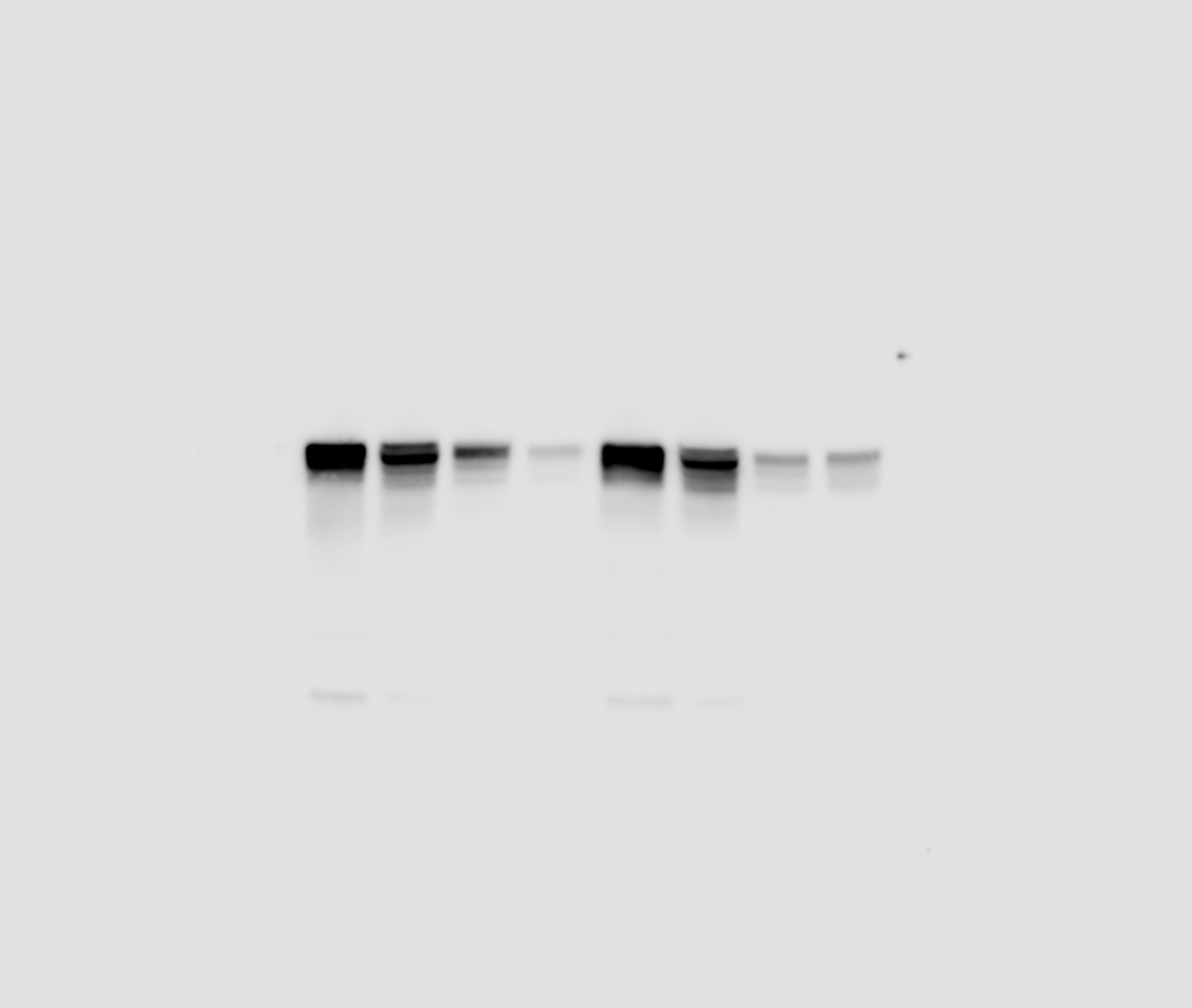

Supplement: Figure 6—source data 2. [file elife-73330-fig6-data2.zip › Figure 6 - Source data 2/Hpylori/HumanQ1K&HumanQ1K,A49V.png]

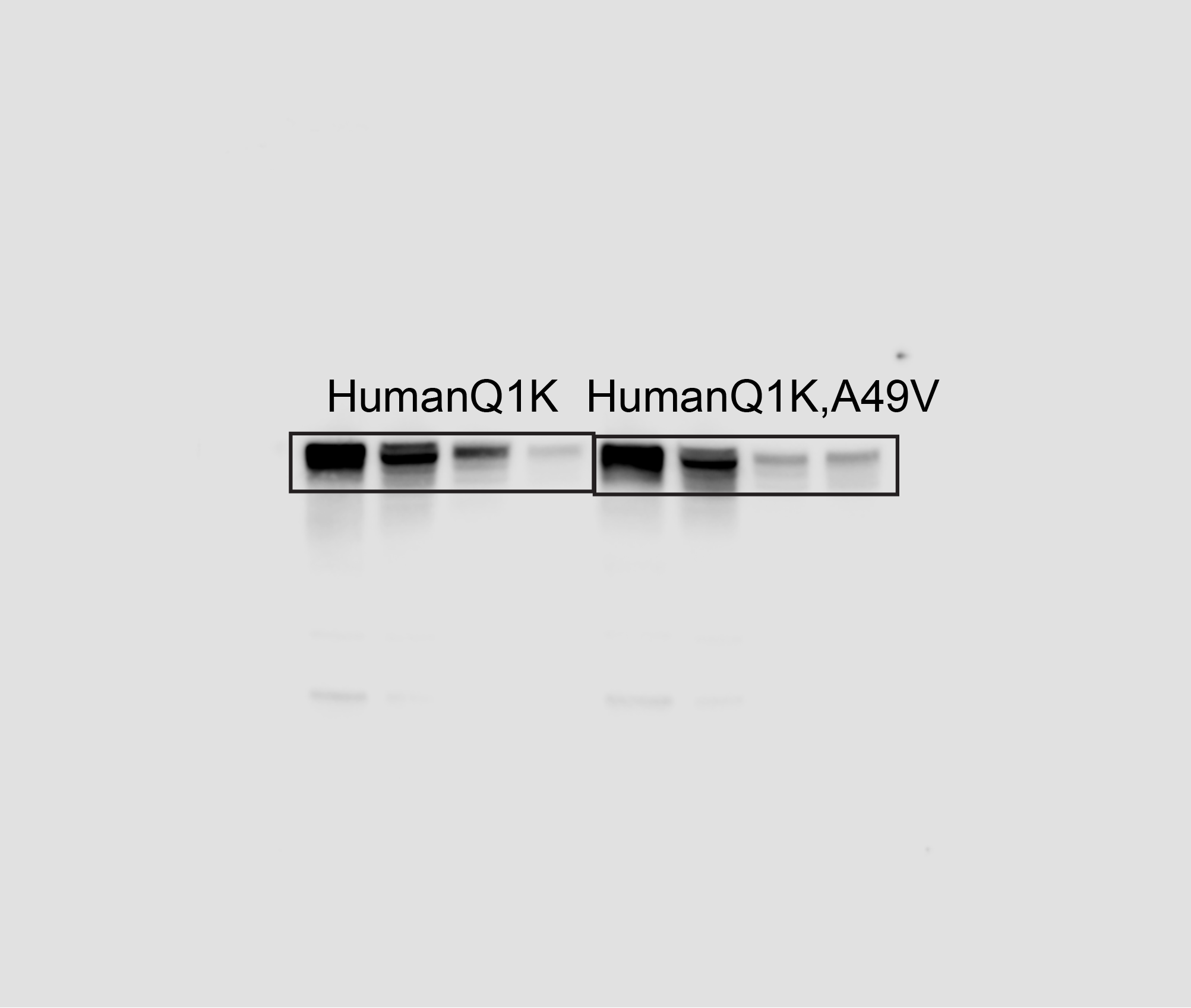

Supplement: Figure 6—source data 2. [file elife-73330-fig6-data2.zip › Figure 6 - Source data 2/Hpylori/HumanQ1K&HumanQ1K,A49V_label.png]

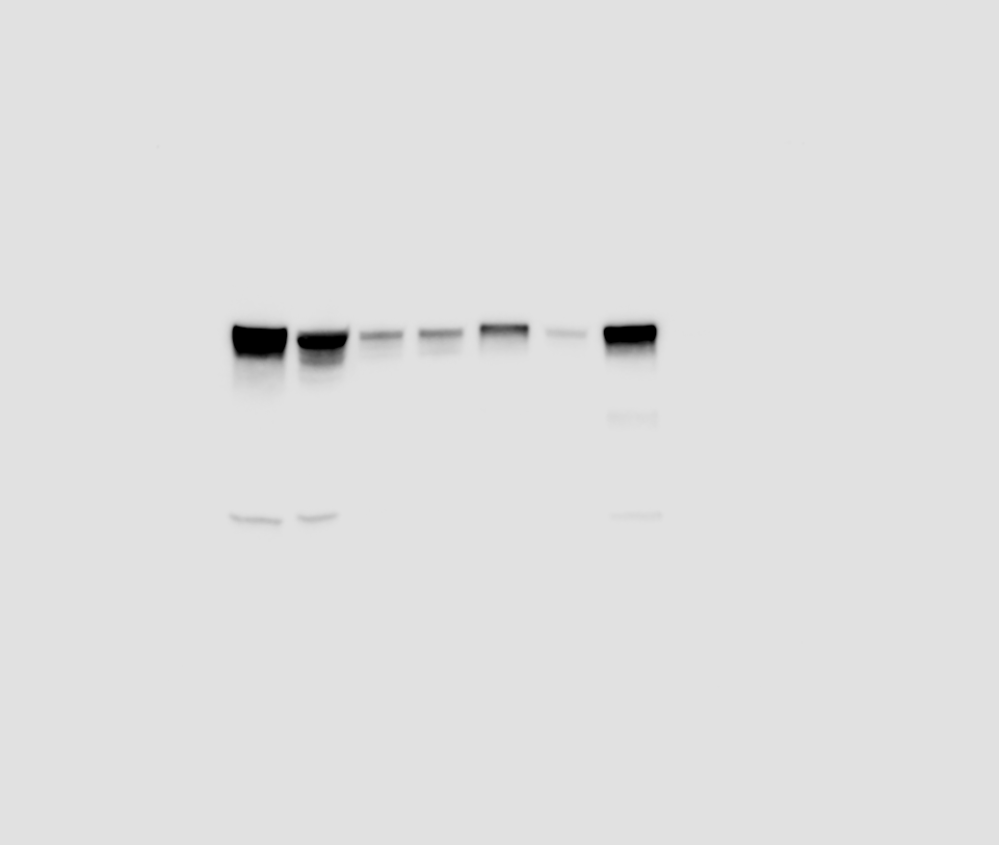

Supplement: Figure 6—source data 2. [file elife-73330-fig6-data2.zip › Figure 6 - Source data 2/Hpylori/HumanQ1K,A49V,Q89H.png]

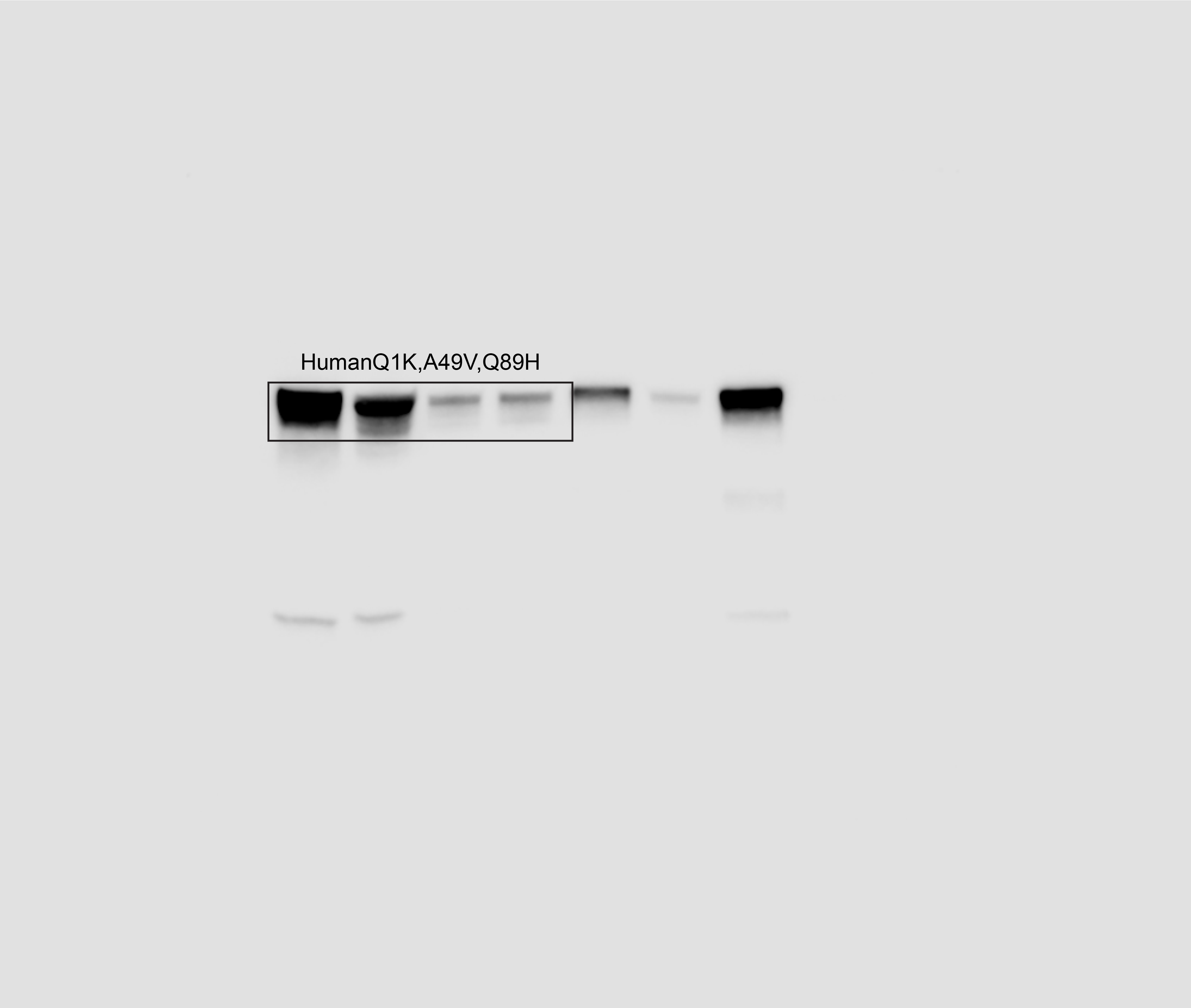

Supplement: Figure 6—source data 2. [file elife-73330-fig6-data2.zip › Figure 6 - Source data 2/Hpylori/HumanQ1K,A49V,Q89H_label.png]

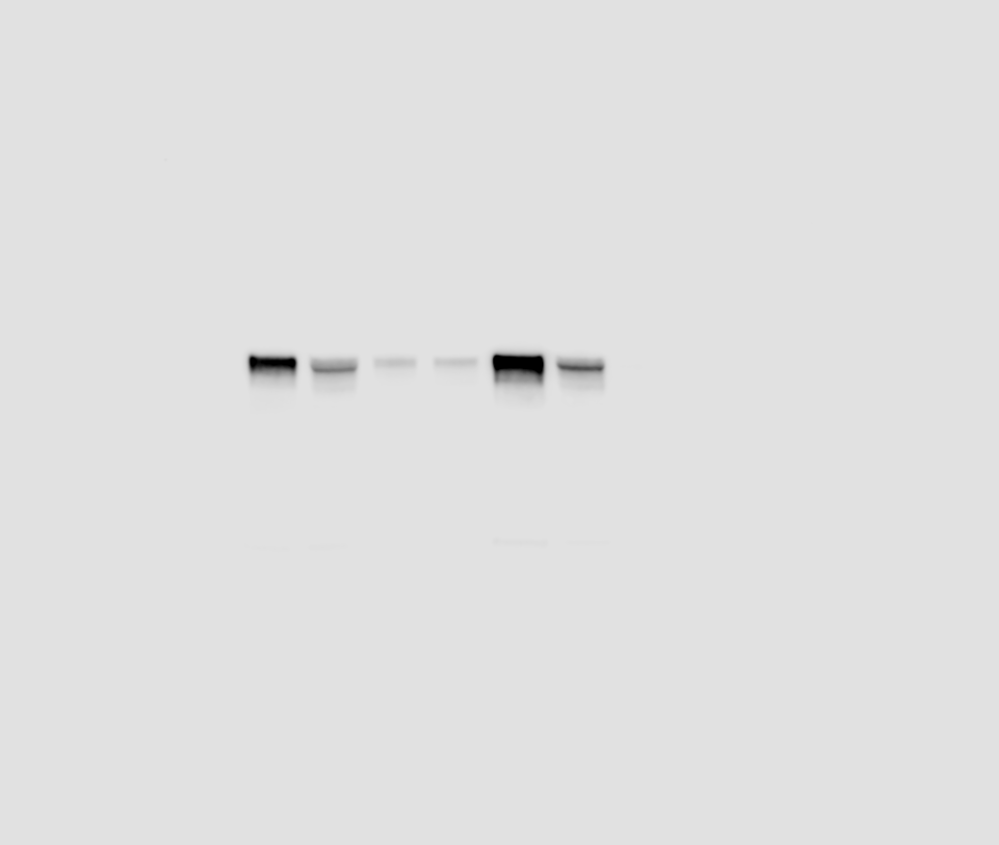

Supplement: Figure 6—source data 2. [file elife-73330-fig6-data2.zip › Figure 6 - Source data 2/Hpylori/HumanQ1K,Q89H.png]

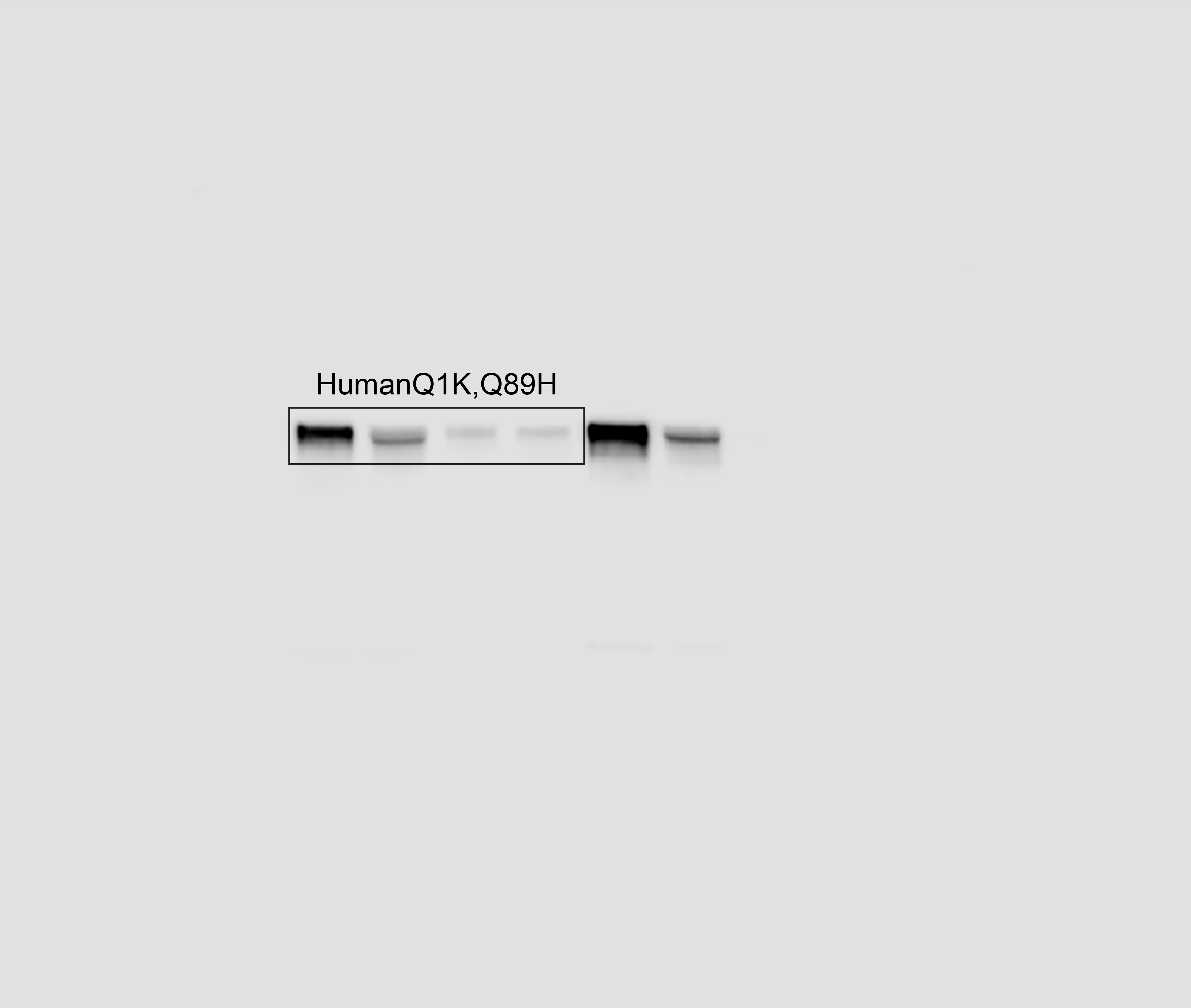

Supplement: Figure 6—source data 2. [file elife-73330-fig6-data2.zip › Figure 6 - Source data 2/Hpylori/HumanQ1K,Q89H_label.png]

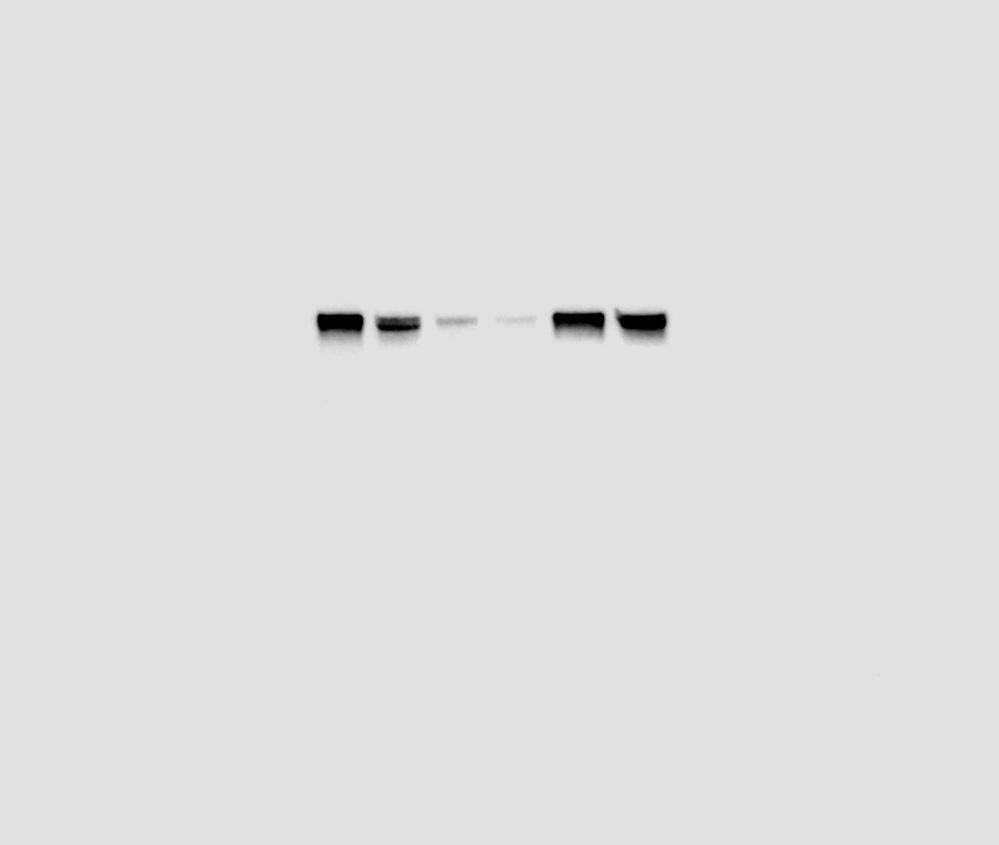

Supplement: Figure 6—source data 2. [file elife-73330-fig6-data2.zip › Figure 6 - Source data 2/Hpylori/HumanQ89H.png]

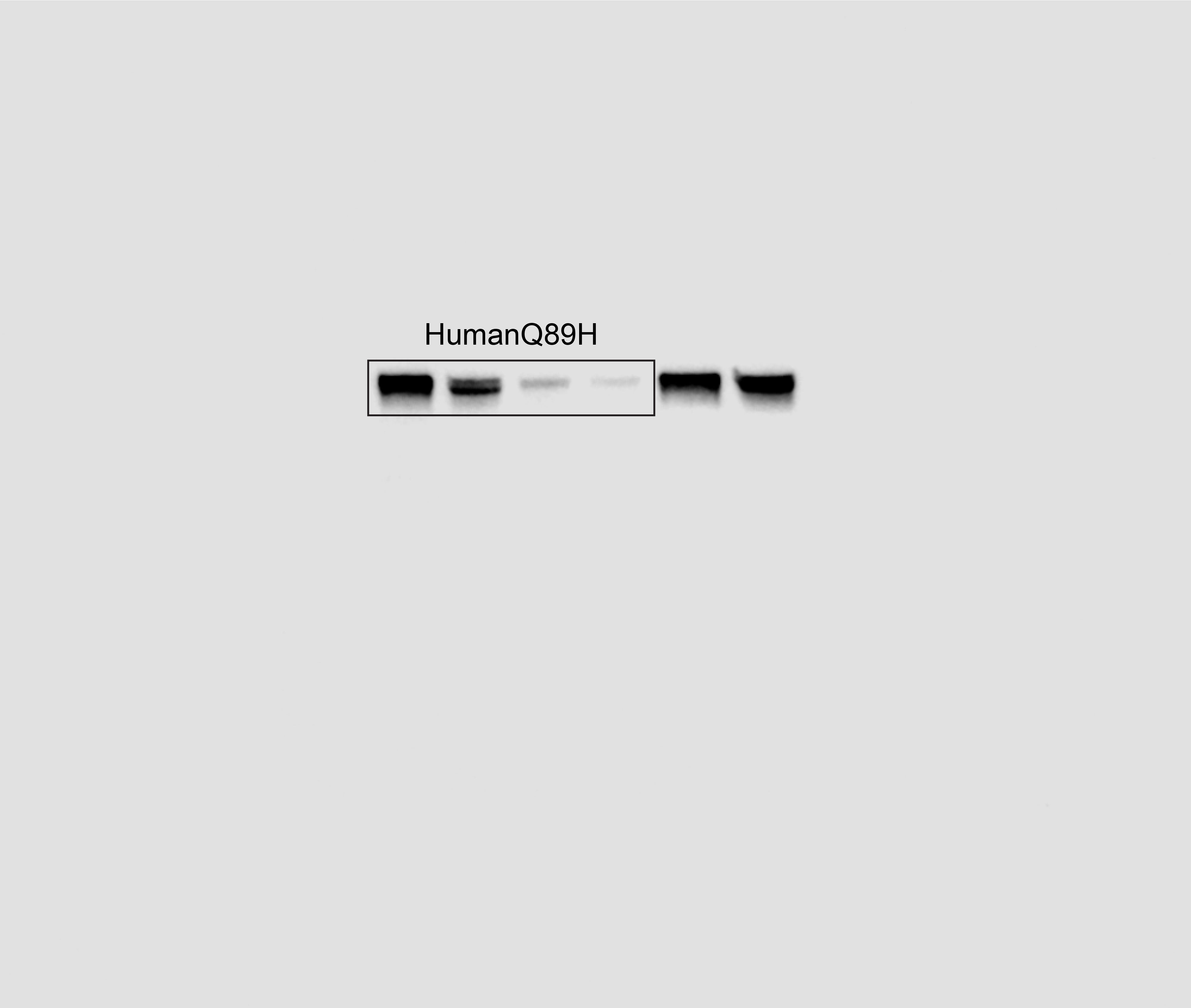

Supplement: Figure 6—source data 2. [file elife-73330-fig6-data2.zip › Figure 6 - Source data 2/Hpylori/HumanQ89H_label.png]

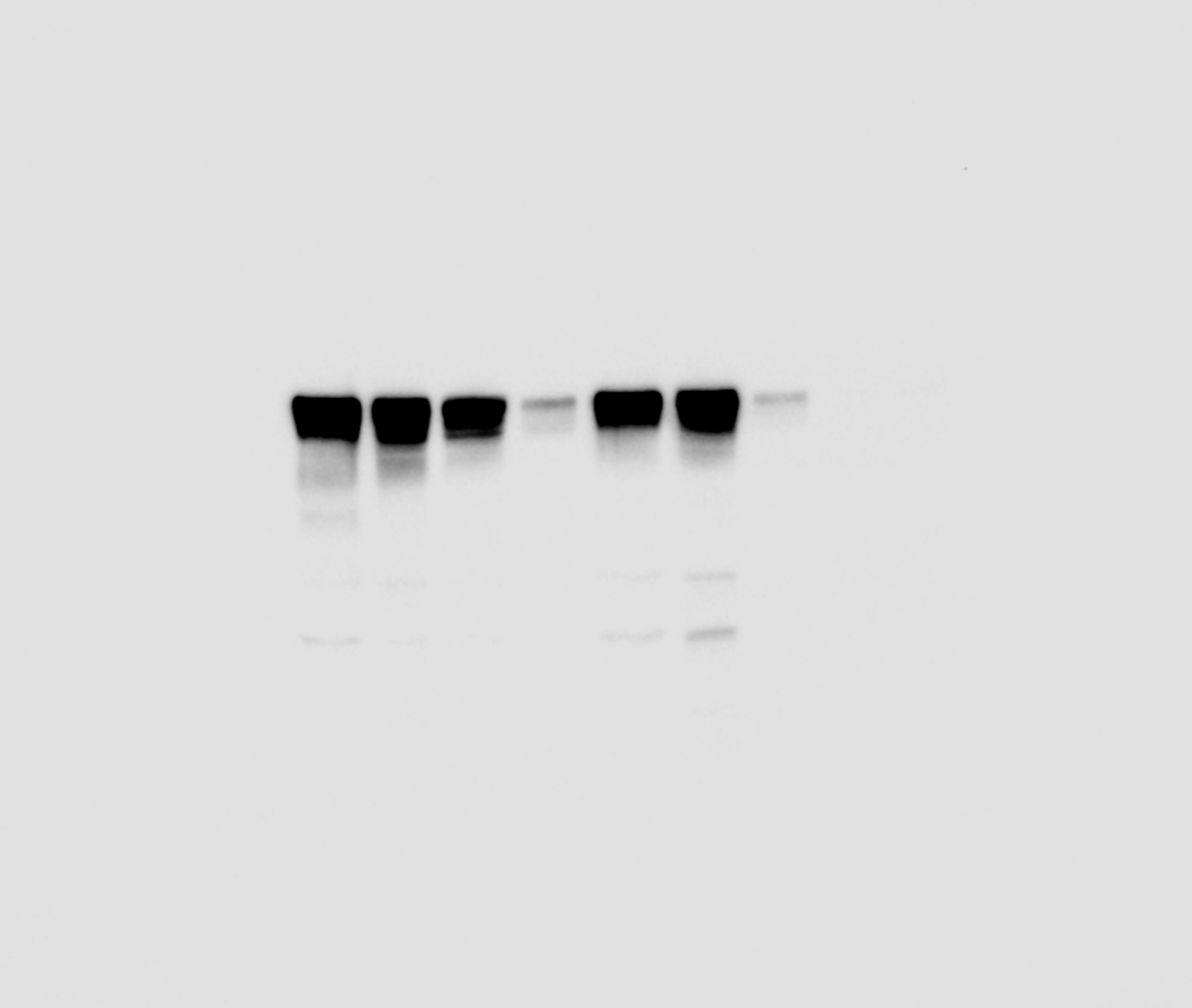

Supplement: Figure 6—source data 2. [file elife-73330-fig6-data2.zip › Figure 6 - Source data 2/Hpylori/HumanREF.png]

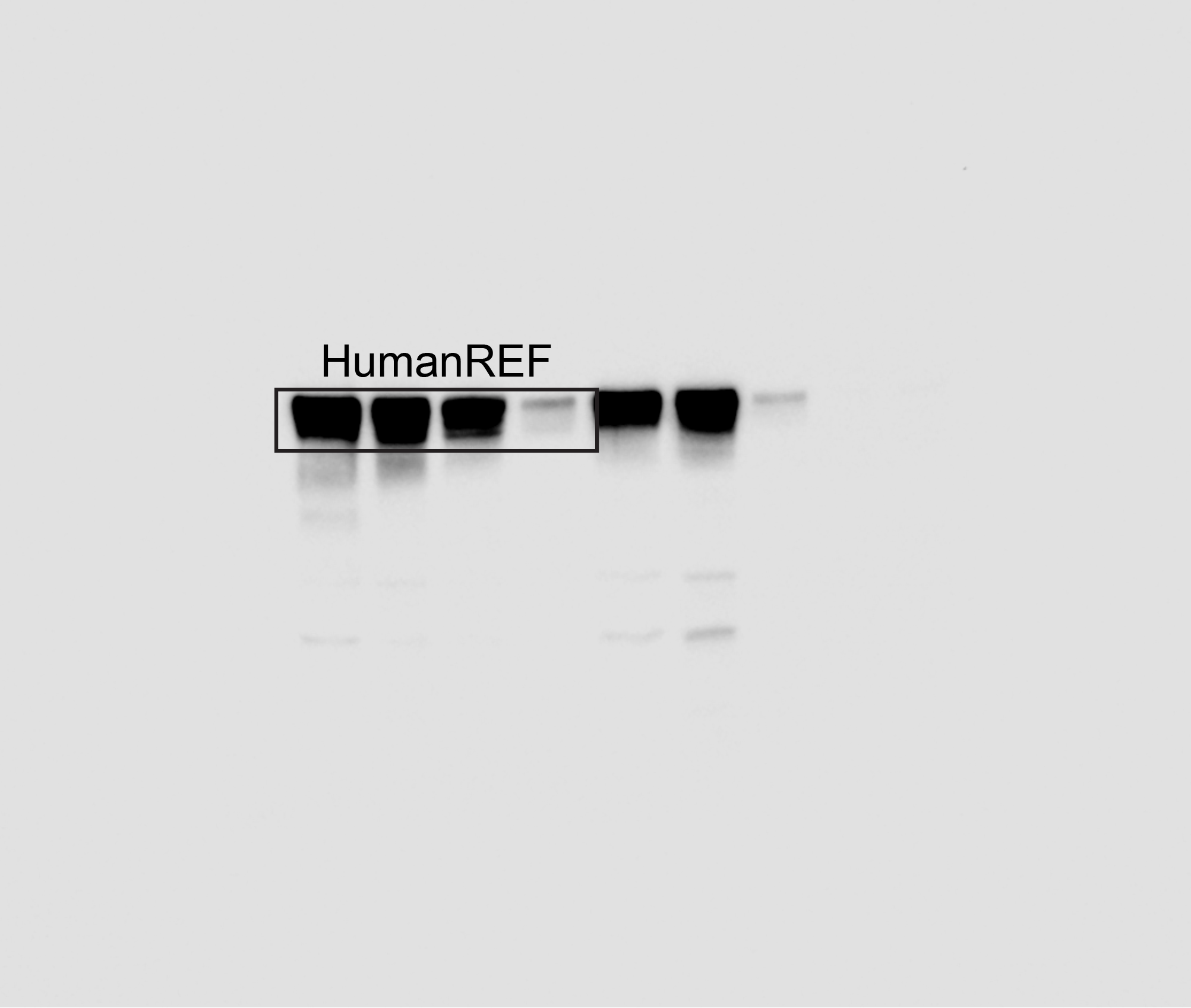

Supplement: Figure 6—source data 2. [file elife-73330-fig6-data2.zip › Figure 6 - Source data 2/Hpylori/HumanREF_label.png]

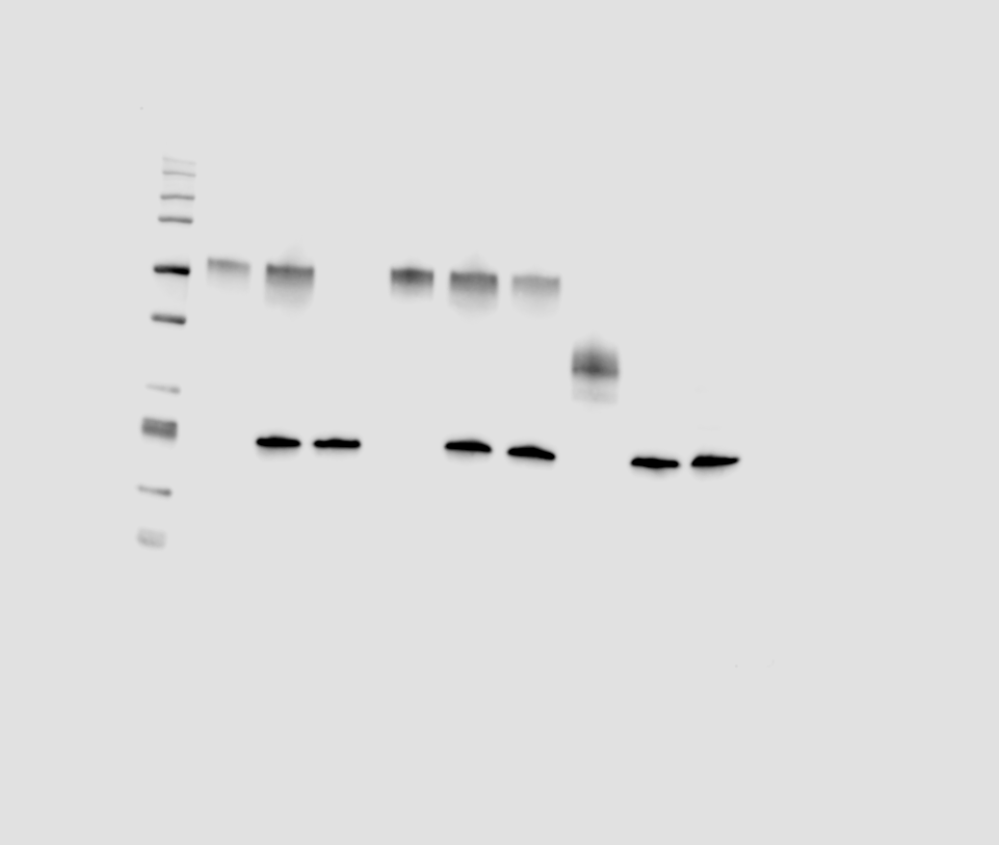

Supplement: Figure 6—source data 2. [file elife-73330-fig6-data2.zip › Figure 6 - Source data 2/Opa/HumanA49V&HumanQ1K.png]

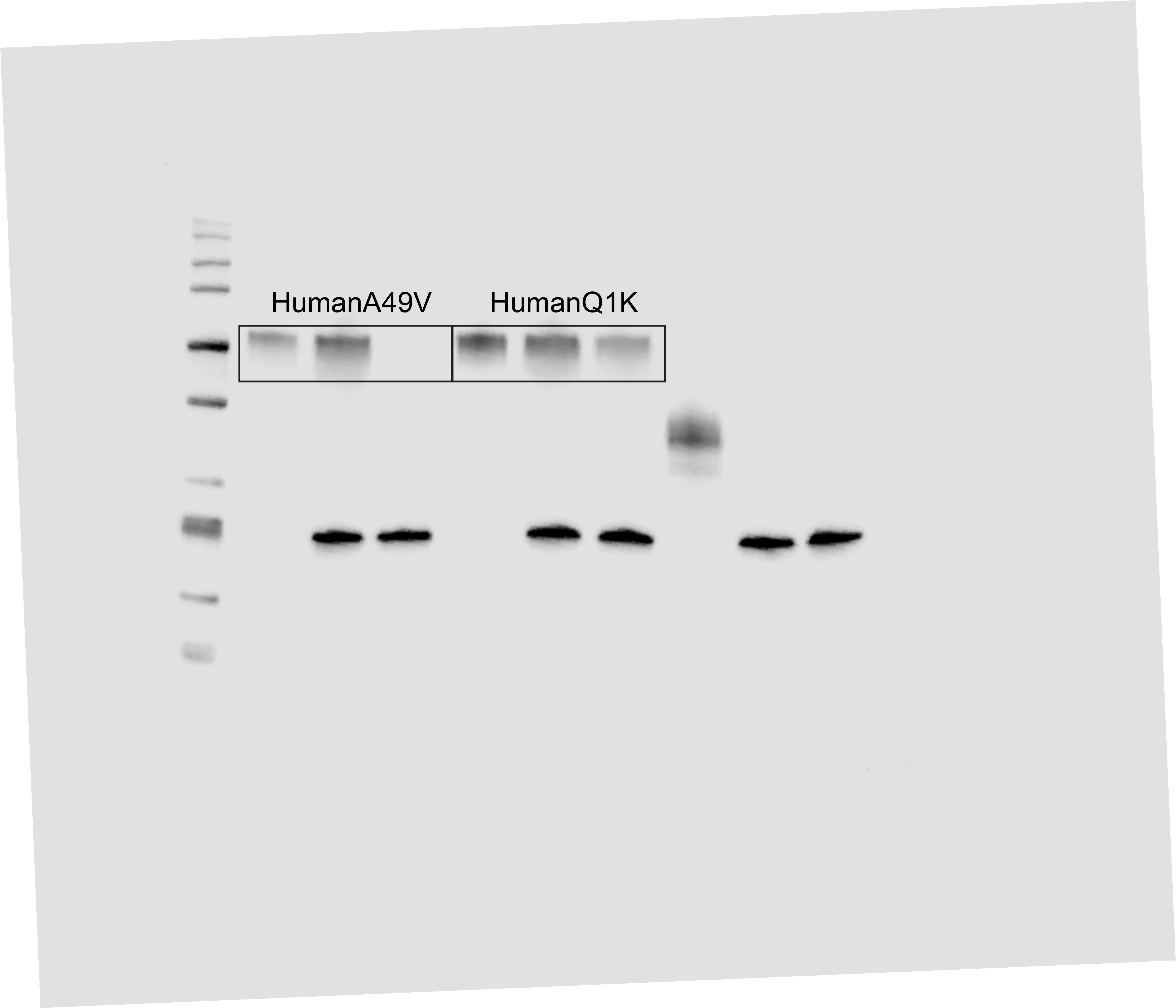

Supplement: Figure 6—source data 2. [file elife-73330-fig6-data2.zip › Figure 6 - Source data 2/Opa/HumanA49V&HumanQ1K_label.png]

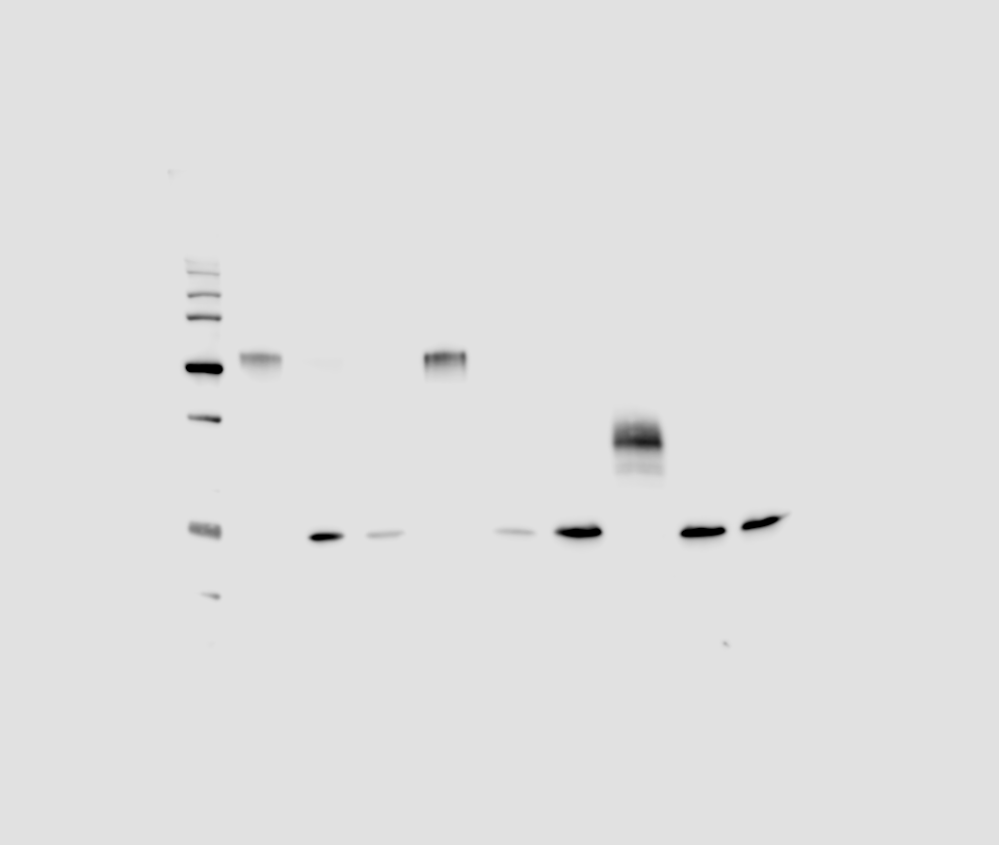

Supplement: Figure 6—source data 2. [file elife-73330-fig6-data2.zip › Figure 6 - Source data 2/Opa/HumanA49V,Q89H&HumanQ1K,Q89H.png]

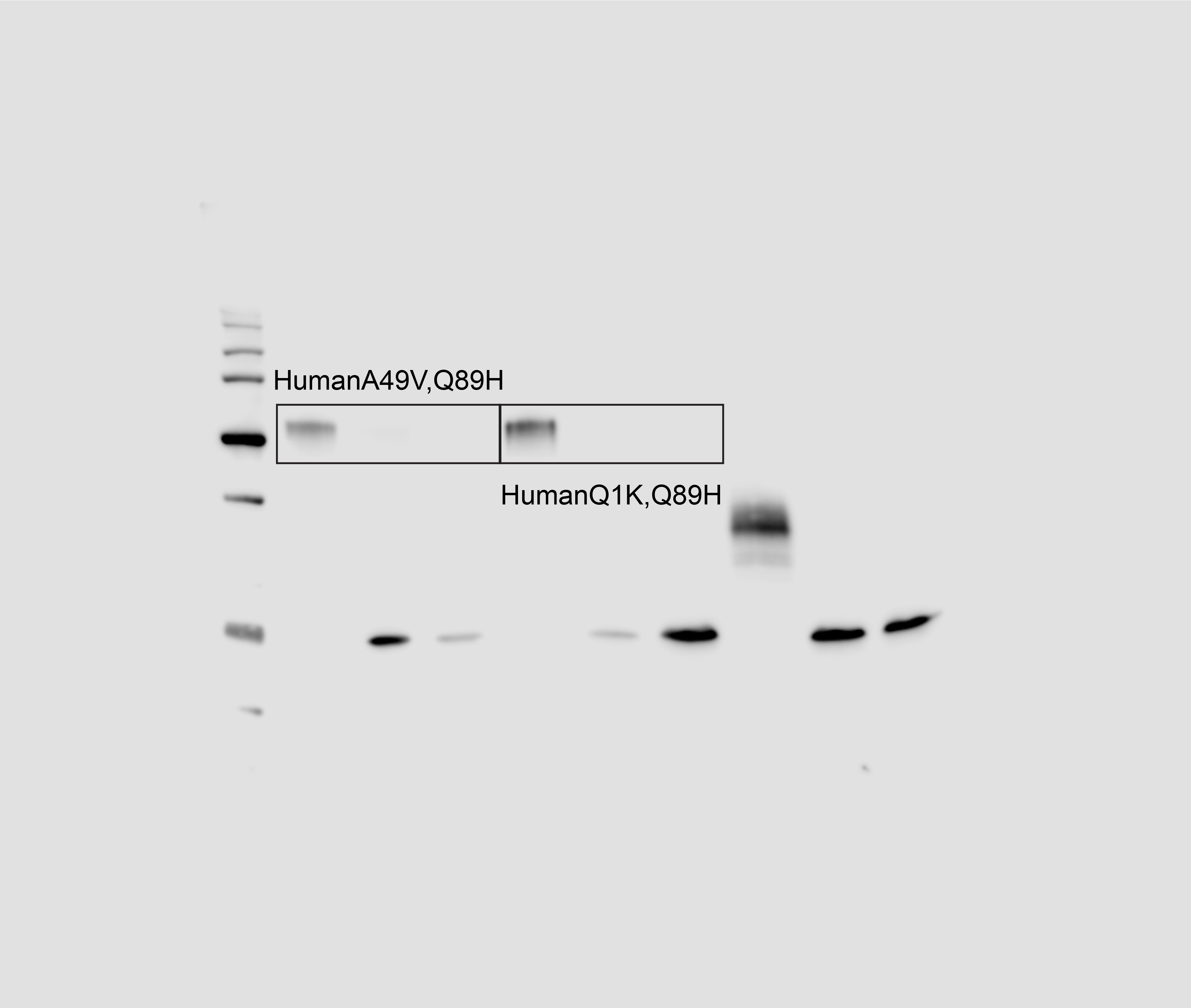

Supplement: Figure 6—source data 2. [file elife-73330-fig6-data2.zip › Figure 6 - Source data 2/Opa/HumanA49V,Q89H&HumanQ1K,Q89H_label.png]

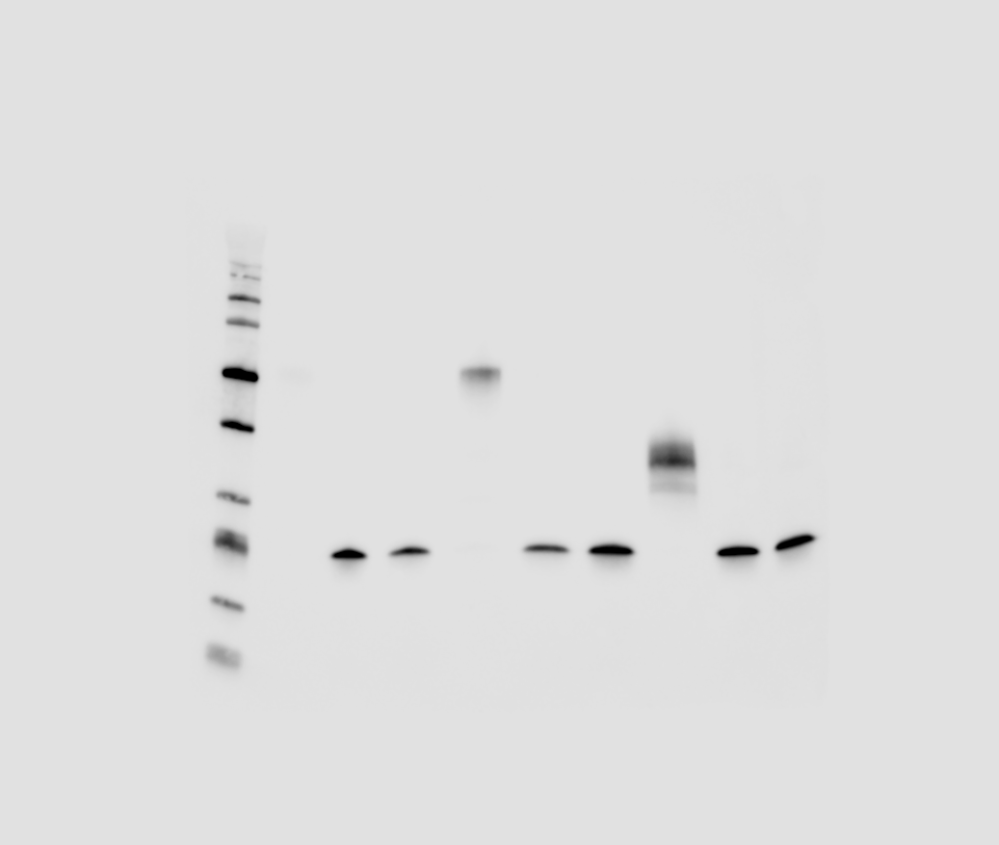

Supplement: Figure 6—source data 2. [file elife-73330-fig6-data2.zip › Figure 6 - Source data 2/Opa/HumanQ1K,A49V,Q89H.png]

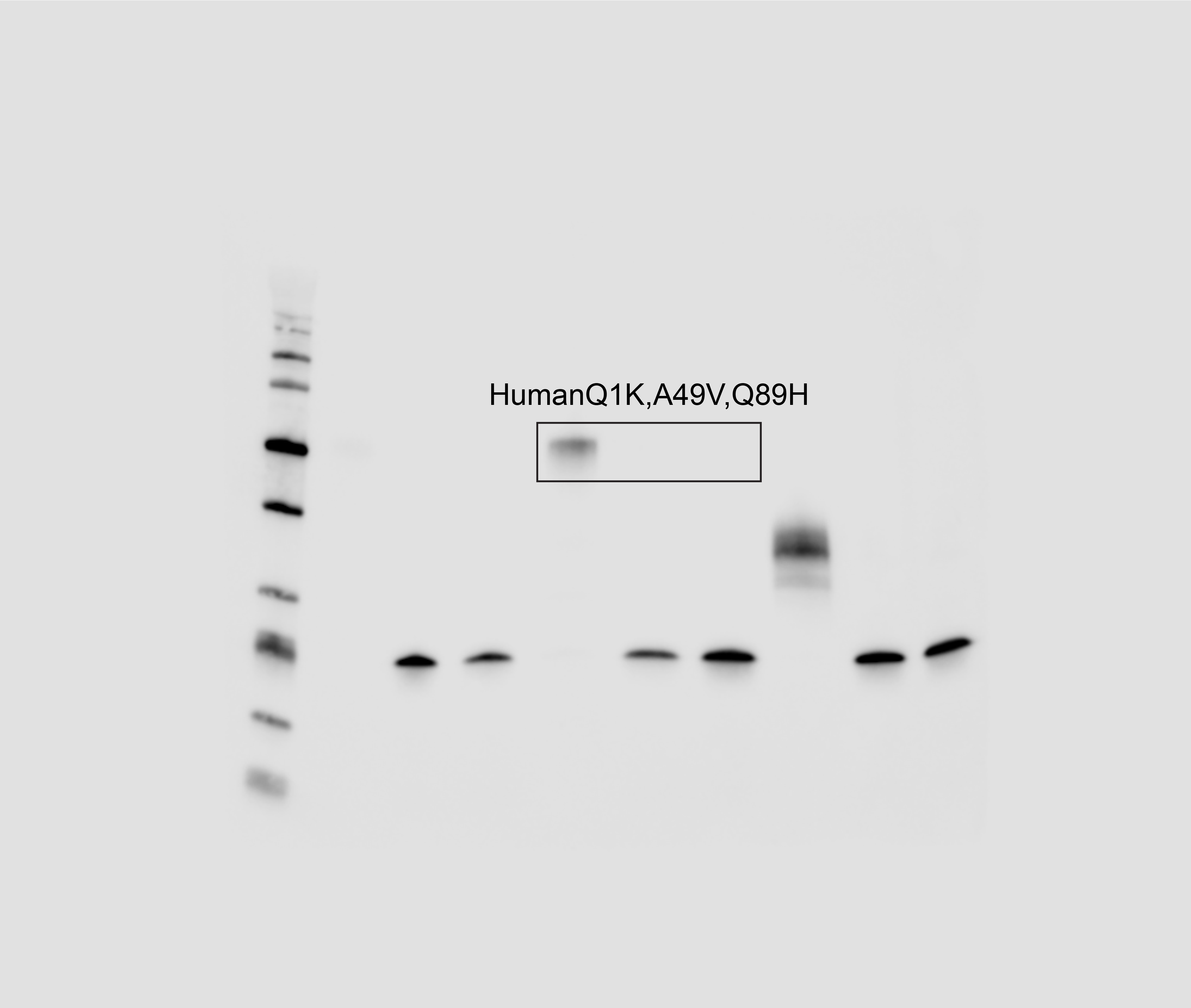

Supplement: Figure 6—source data 2. [file elife-73330-fig6-data2.zip › Figure 6 - Source data 2/Opa/HumanQ1K,A49V,Q89H_label.png]

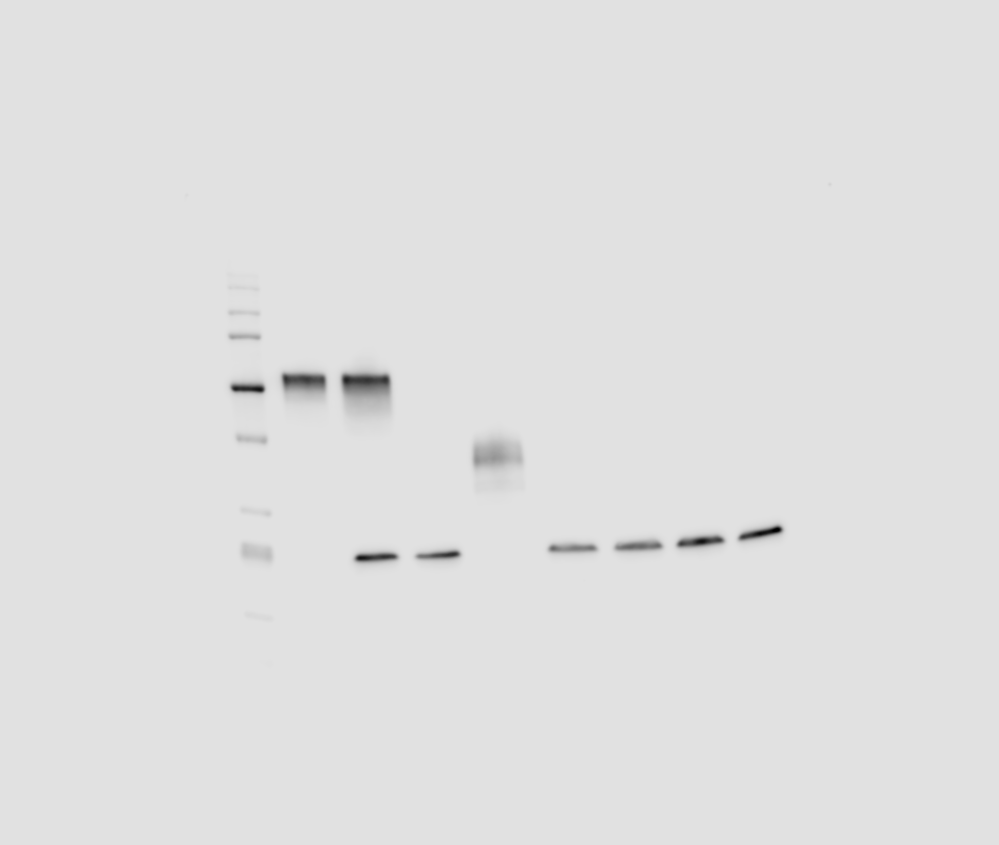

Supplement: Figure 6—source data 2. [file elife-73330-fig6-data2.zip › Figure 6 - Source data 2/Opa/HumanQ1K,A49V.png]

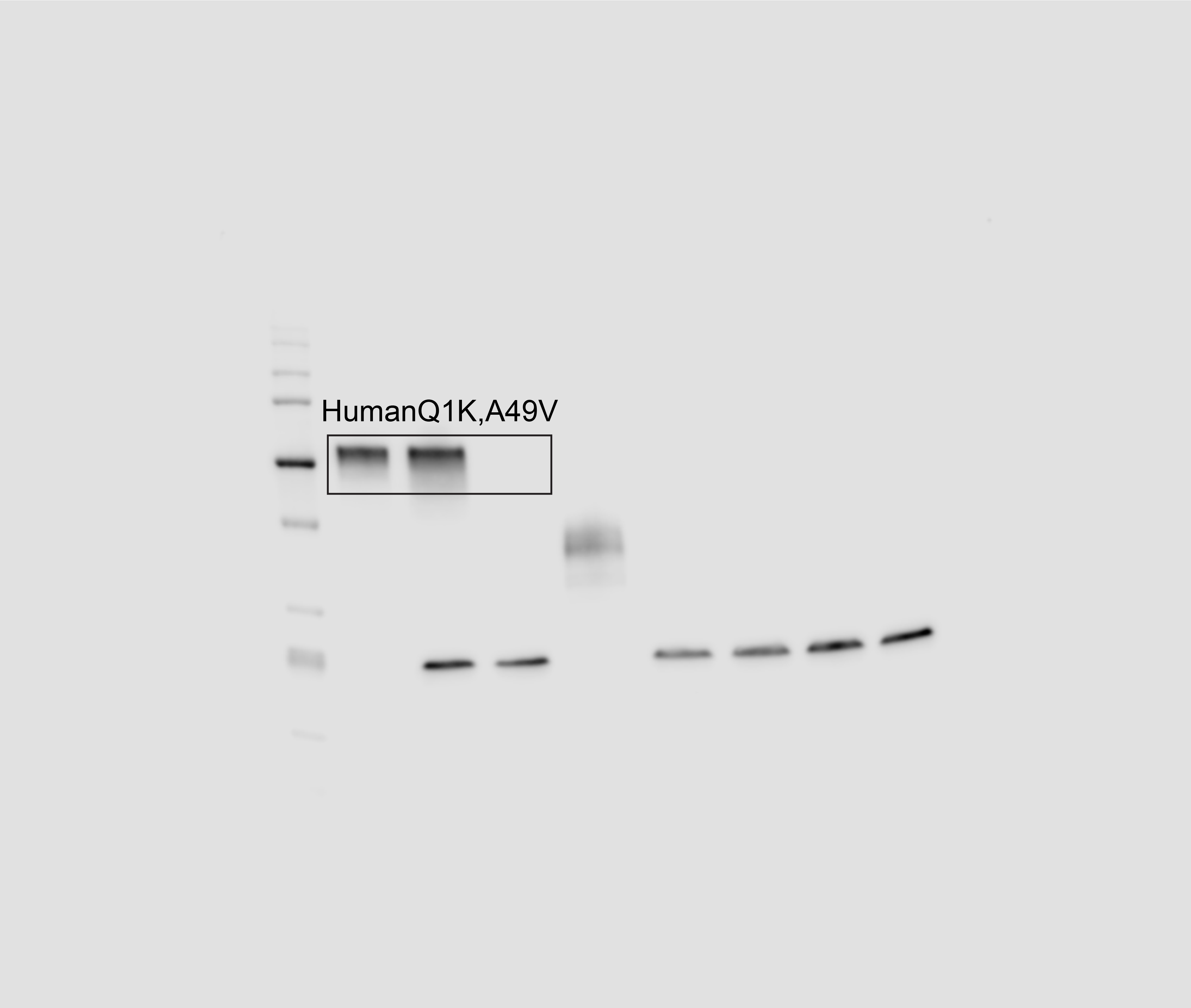

Supplement: Figure 6—source data 2. [file elife-73330-fig6-data2.zip › Figure 6 - Source data 2/Opa/HumanQ1K,A49V_label.png]

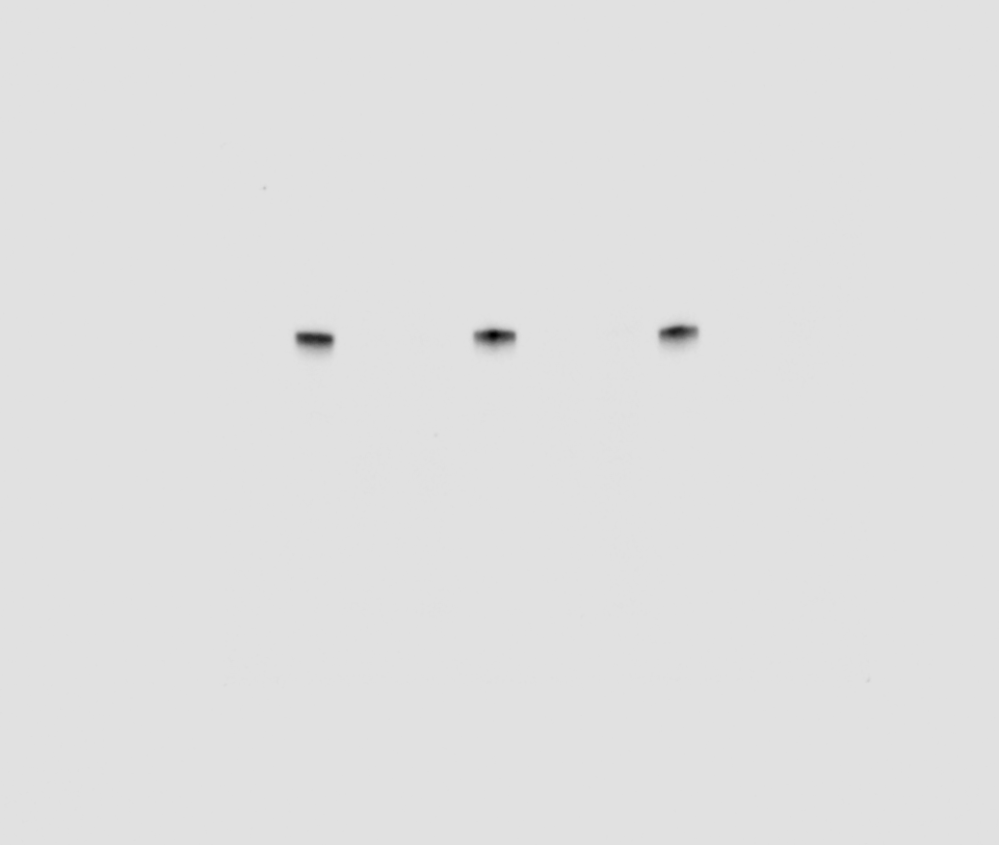

Supplement: Figure 6—source data 2. [file elife-73330-fig6-data2.zip › Figure 6 - Source data 2/Opa/HumanQ89H.png]

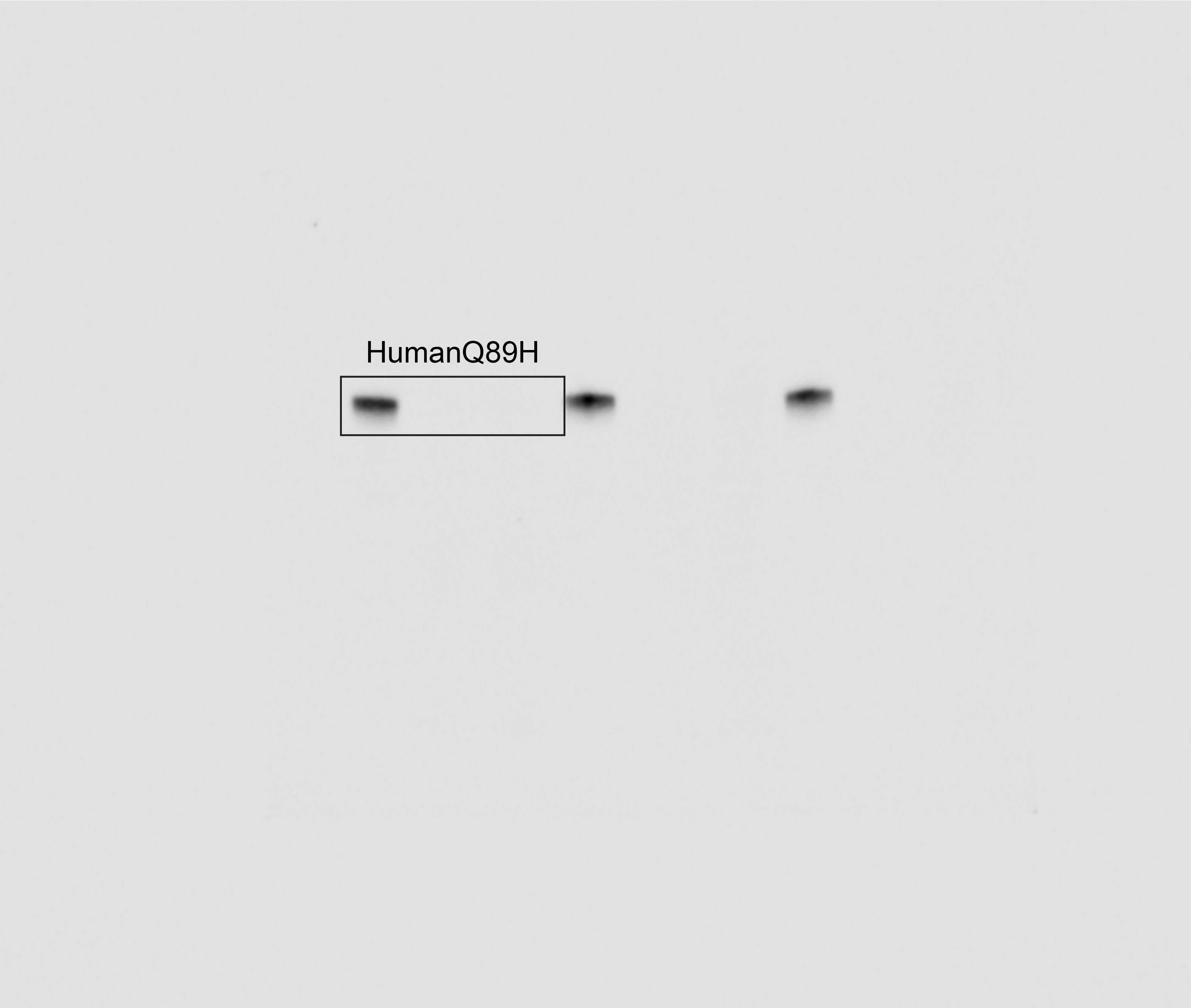

Supplement: Figure 6—source data 2. [file elife-73330-fig6-data2.zip › Figure 6 - Source data 2/Opa/HumanQ89H_label.png]

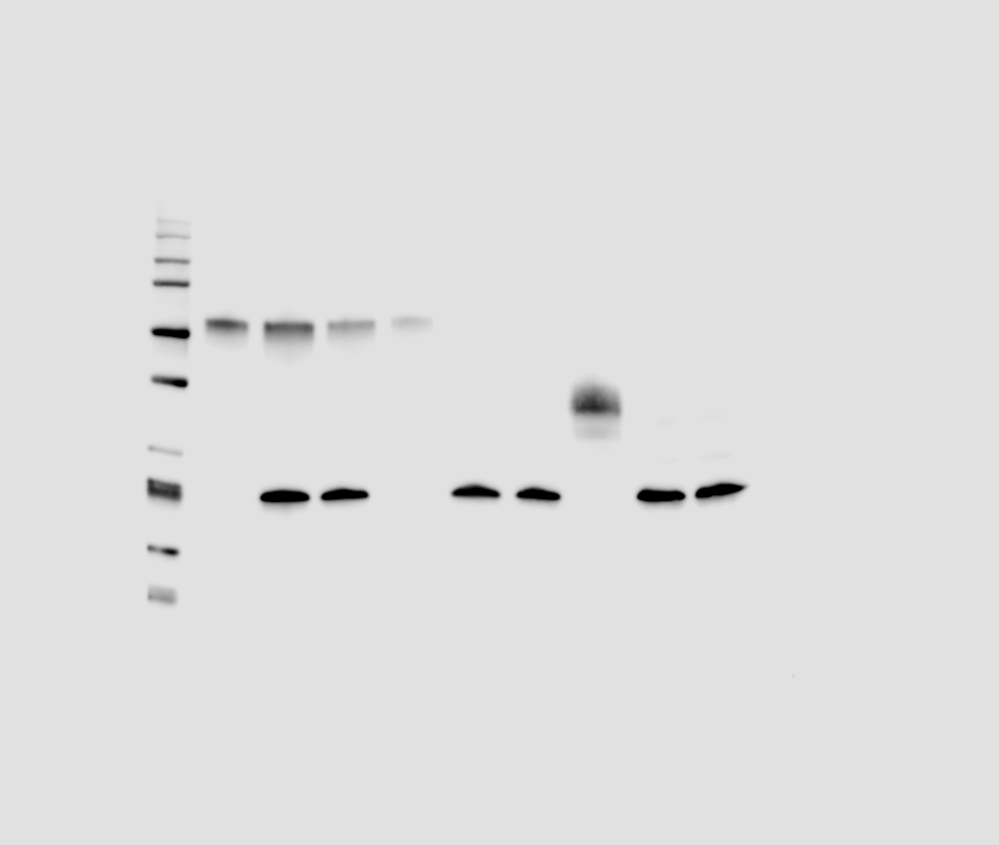

Supplement: Figure 6—source data 2. [file elife-73330-fig6-data2.zip › Figure 6 - Source data 2/Opa/HumanREF.png]

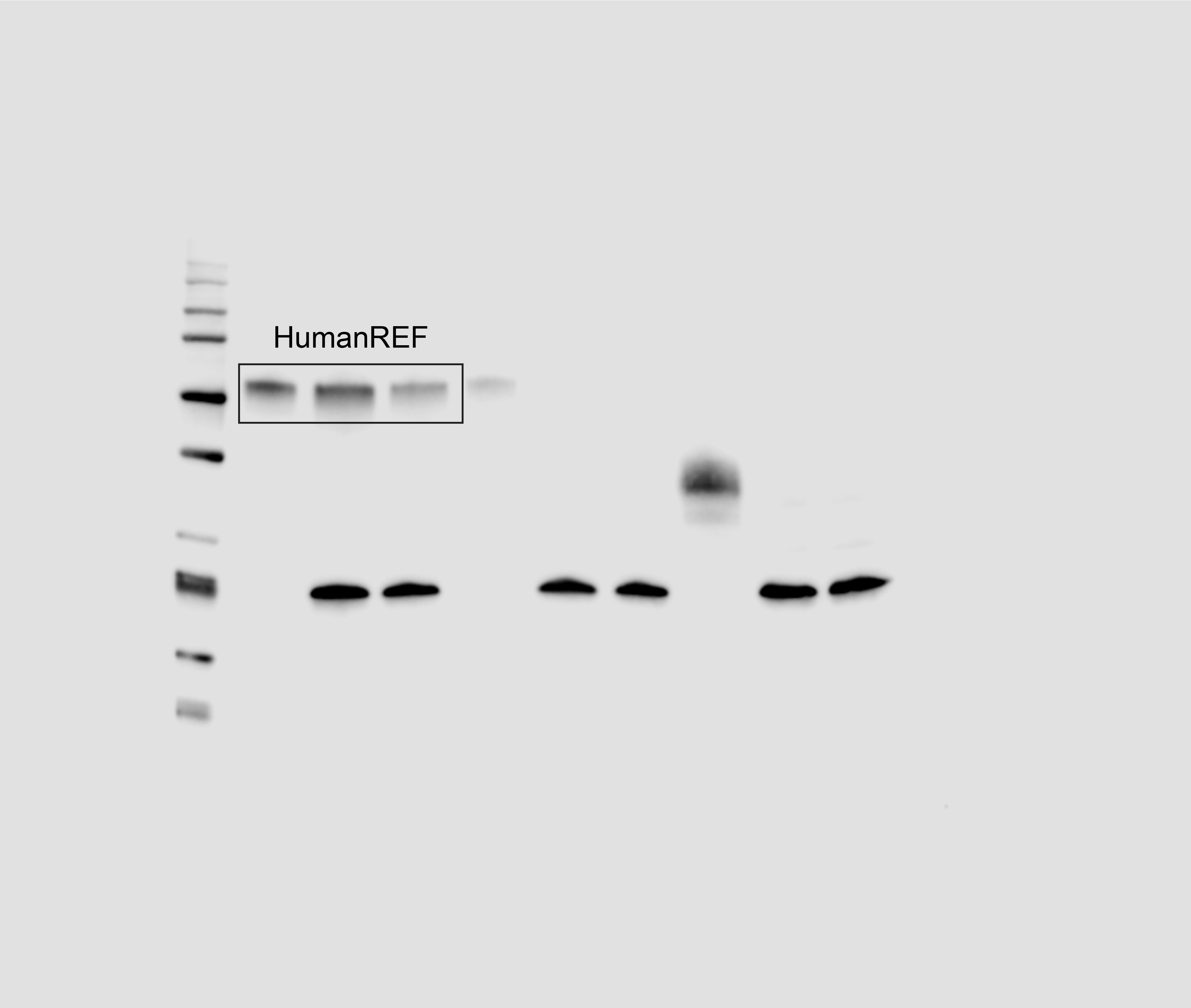

Supplement: Figure 6—source data 2. [file elife-73330-fig6-data2.zip › Figure 6 - Source data 2/Opa/HumanREF_label.png]
